# Global Burden of Schizophrenia Among Women of Reproductive Age from 1990 to 2021: Trends, Inequalities, and Projections to 2040

**DOI:** 10.1177/26884844251379002

**Published:** 2025-09-16

**Authors:** Lei Tang, Xinyue Chen, Hangyu Li, Shiji Peng, Ying Li, Mengqin Dai, Lu Liu, Nian Liu

**Affiliations:** ^1^ Mental Health Center, Affiliated Hospital of North Sichuan Medical College, Nanchong, China.; ^2^ School of Psychiatry, North Sichuan Medical College, Nanchong, China.; ^3^ Key Laboratory of Digital-Intelligent Disease Surveillance and Health Governance, North Sichuan Medical College, Nanchong, China.; ^4^ Department of Radiology, Affiliated Hospital of North Sichuan Medical College, Nanchong, China.

**Keywords:** schizophrenia, women of reproductive age, global burden of disease, health inequality, projected future burden

## Abstract

**Background:**

Schizophrenia poses a significant health challenge for women of reproductive age globally. This study aimed to analyze the global burden of schizophrenia among this population from 1990 to 2021 and project trends to 2040.

**Methods:**

We utilized data from the Global Burden of Disease Study 2021 to examine the incidence, prevalence, and disability-adjusted life years (DALYs) of schizophrenia among women aged 15–49 years. We analyzed trends across Sociodemographic Index (SDI) regions, performed decomposition analysis, assessed health inequalities, and projected future burdens.

**Results:**

From 1990 to 2021, global incidence cases increased by 35.68% to 513,255, while age-standardized incidence rates slightly decreased by 1.00%. Prevalence cases rose by 55.82% to 7,541,989. Low SDI regions experienced the highest percentage increases in burden. Decomposition analysis revealed population growth as the primary driver of increased burden globally (85.4% for DALYs), with varying patterns across SDI regions. Health inequality analysis showed a growing concentration of prevalence and DALYs in higher SDI regions. Projections indicate continued global increases in absolute burden through 2040, with decreasing age-standardized rates.

**Conclusions:**

The burden of schizophrenia among women of reproductive age has increased substantially, driven primarily by population growth, with significant disparities across SDI regions. Tailored interventions addressing region-specific drivers are needed to mitigate the growing impact of schizophrenia on this population.

## Introduction

Schizophrenia is a disabling mental illness. Its characteristic symptoms include positive and negative symptoms and cognitive deficit. The positive symptoms include hallucination, delusion, speech disorder, and tension behavior. Negative symptoms include decreased motivation and performance. In addition, individuals with schizophrenia had impairments in executive function, cognitive function, memory ability, and mental processing speed.^1^ Schizophrenia affects about 1% of the world’s population and is one of the top 10 causes of disability worldwide.^2^ Previous studies have reported that people with schizophrenia have a 5% to 10% lifetime risk of death by suicide,^3^ and the overall weighted average of potential years of life lost due to schizophrenia is 14.5 (95% confidence interval [CI]: 11.2–17.8),^4^ placing a significant burden on the socioeconomic and health care systems. Since 1990, the number of cases of schizophrenia and disability-adjusted life years (DALY) have increased globally, with an increase of 36.69% and 62.46%, respectively, compared to 1990.^5^ Findings from a study on the global epidemiology and burden of schizophrenia show that, the global age-standardized prevalence of schizophrenia was estimated at 0.28% (95% Uncertainty Interval (UI): 0.24–0.31), with cases rising from 131,000 (95%UI: 11.6–14.8 million) cases in 1990 to 20.9 (95%UI: 18.5–23.4) million cases, of which schizophrenia contributed 13.4 (95%UI: 9.9–16.7) million Years Lived with Disability (YLD) to the global disease burden, equivalent to 1.7% of the total global YLD in 2016.^6^ In addition, studies have shown that estradiol production in women of childbearing age may have protective effects against the onset of schizophrenia.^7^ A randomized controlled trial demonstrated that adjunctive transdermal estradiol treatment significantly improved positive symptoms in women with schizophrenia, with a mean positive PANSS score reduction of 1.0 point compared to placebo.^8^ Another large-scale randomized trial found that both 100 and 200 µg estradiol patches resulted in greater decreases in PANSS positive, general and total symptoms compared with placebo, with the largest effect size of 0.44 for positive symptoms in the 200 µg group.^9^ However, the complex hormonal fluctuations during reproductive years, including cyclic variations in estradiol levels, may influence the clinical presentation and severity of schizophrenia symptoms in women of reproductive age (WRA).^10^ Among new diagnoses of schizophrenia, epidemiological data suggest varying incidence patterns between women and men, with some studies reporting different age-group-specific patterns rather than a uniform higher incidence in women.^2^

There are differences in the clinical presentation of schizophrenia in women compared to men, and women with schizophrenia may have more pronounced affective and depressive symptoms and may experience more emotional fluctuations and relapses as they age.^11,12^ A study examining menstrual cycle-related changes found that estradiol levels negatively correlated with negative symptom severity in women with schizophrenia during the mid-luteal phase, highlighting the impact of hormonal fluctuations on symptom presentation.^10^ WRA face unique vulnerabilities during critical life stages including pregnancy, postpartum period, and family formation. Population-based studies have demonstrated that women with schizophrenia experience significantly higher rates of pregnancy complications (adjusted OR = 1.41, 95% CI 1.31–1.51), delivery complications (OR = 1.18, 95% CI 1.09–1.29), and adverse neonatal outcomes compared to controls.^13^ Additionally, a qualitative study revealed that reproductive concerns create substantial psychological stress for women with schizophrenia, as they face conflicts between the significance of reproduction and multiple obstacles including potential risks to offspring and difficulties in child-rearing.^14^ Furthermore, adverse childhood experiences are significantly more prevalent in women with schizophrenia (mean 4.57 ACEs) compared to women without the disorder (mean 2.51 ACEs), and these experiences are associated with increased symptom burden of depression and anxiety in adulthood.^15^ Moreover, schizophrenia may affect their fertility plans. Existing research suggests that,^16–18^ schizophrenia during pregnancy is associated with adverse pregnancy outcomes for both mother and fetus, including gestational diabetes mellitus, hypertensive disorders of pregnancy, preterm labor, and low birth weight.

In addition, hormonal factors and reproductive health considerations significantly affect the clinical management and burden of schizophrenia in women of childbearing age. Studies have shown that antipsychotic medications can interact with reproductive health through prolactin elevation, with long-term use of prolactin-increasing antipsychotics (≥5 years) associated with increased breast cancer risk (adjusted OR 1.56, 95% CI 1.27–1.92) compared to minimal exposure.^19^ Furthermore, menopausal hormone therapy has demonstrated protective effects against psychosis relapse in women with schizophrenia, with a 16% lower relapse risk (adjusted HR = 0.84, 95% CI 0.78–0.90) during hormone therapy use, particularly effective in women aged 40–55 years.^20^ Additionally, the complex reproductive concerns faced by women with schizophrenia, including fears about medication effects on pregnancy and offspring, create substantial psychological burden that requires specialized clinical attention.^14^ The hormonal fluctuations during menstrual cycles also influence symptom severity, with estradiol levels showing negative correlations with negative symptom severity.^10,21^ Therefore, assessing the burden caused by schizophrenia in WRA, including these reproductive health considerations, is essential for the implementation of precise intervention activities on a global scale.

It is worth highlighting that relatively few studies have centered on the incidence and trends of schizophrenia among WRA,^22,23^ and a comprehensive assessment of the burden of schizophrenia within this population remains largely underexplored. Although global attention to schizophrenia has been gradually rising, there remain numerous deficiencies in the allocation of specific medical resources and the implementation of intervention strategies. Ideally, an optimal mental health service system should be established to provide precise and personalized medical services for women of reproductive age with schizophrenia. However, timely and effective treatment for schizophrenia is frequently inaccessible in most parts of the world, especially in regions with limited resources. For the specific group of WRA, they are confronted not only with the problems caused by the disease itself but also with the pressures from family, fertility, and other aspects, thereby further exacerbating the disease burden. This situation requires urgent attention to address the multifaceted challenges posed by schizophrenia among WRA. Therefore, based on the available research data, we conducted an analysis and prediction of the incidence, prevalence, and DALYs of schizophrenia among WRA at the global, regional, and national levels from 1990 to 2021, comparing the distribution and variations of the schizophrenia burden across different age groups.

## Materials and Methods

### Data source

Data for this study were obtained from the Global Burden of Disease Study 2021 database, which is publicly accessible through the Institute for Health Metrics and Evaluation website (https://ghdx.healthdata.org/gbd-results-tool). This comprehensive database provides estimates for 204 countries and territories, 371 diseases and injuries, and 88 risk factors using standardized methods for data collection, harmonization, and validation. The Global Burden of Disease (GBD) methodology employs systematic literature reviews, vital registration systems, surveillance data, and survey data, which are then processed through advanced statistical modeling techniques including spatiotemporal Gaussian process regression and cause of death ensemble modeling to ensure data comparability and reliability across different regions and time periods. We selected “Cause of death or injury” under GBD Estimate, “Incidence,” “Prevalence,” and “DALYs” under Measure, and both “Number” and “Rate” under Metric. “Number” represents the absolute count of the corresponding measure in the population, while “Rate” denotes the measure per 100,000 population. Schizophrenia was chosen as the Cause, and the population was limited to women of reproductive age (15–49 years), consistent with our study’s focus on analyzing the global burden among this specific demographic group.^24–26^

### Disease definition and classification

According to the GBD 2021 classification, diseases and injuries are typically categorized into four main levels. Schizophrenia is classified under the first level of noncommunicable diseases, the second level of mental disorders, and the third level of specific mental health conditions. It is coded as F20 in the International Classification of Diseases (ICD)−10 and 6A20 in ICD-11. In the Global Burden of Disease Study 2021, schizophrenia is defined using ICD-10 codes F20-F20.9 and F25-F25.9, which encompass schizophrenia and schizoaffective disorders respectively. The GBD methodology for schizophrenia data collection and ascertainment utilizes multiple data sources including vital registration systems, surveillance data, hospital discharge records, and epidemiological studies, which are then harmonized using both ICD and DSM diagnostic criteria to ensure global comparability. The systematic data collection process involves comprehensive literature reviews of population-based studies, clinical registries, and administrative health databases, followed by rigorous statistical modeling to account for variations in diagnostic practices and health care systems across different countries and regions.^24,26^

### Estimated annual percentage change model construction

The estimated annual percentage change (EAPC) was calculated to describe long-term trends in the age-standardized rates (ASR) of disease burden. This was done by fitting a simple linear regression model of the natural logarithm of ASR against year. The slope of the regression line represents the EAPC, indicating the annual percentage change. The EAPC and its 95% CI were derived from the regression coefficient and its standard error.

### Health inequality analysis

To investigate health inequalities in the global disease burden, we employed the Slope Index of Inequality (SII) and the Concentration Index. The SII measures absolute inequality in health variables across socioeconomic groups, representing the health difference between the lowest and highest socioeconomic status groups. Specifically, the SII is calculated using weighted least squares regression where the health outcome (prevalence or DALYs) is regressed against the relative rank of each SDI region, weighted by the population size. The formula is: SII = β_1_, where β_1_ is the regression coefficient representing the absolute difference in health outcomes between the hypothetical lowest-ranked (relative rank = 0) and highest-ranked (relative rank = 1) populations. The Concentration Index quantifies the degree of inequality in health variables across socioeconomic groups, ranging from −1 to 1, with 0 indicating perfect equality. It is calculated as: CI = (2/µ) × Cov(h,r), where µ is the mean of the health variable, h represents the health outcome for each SDI region, and r is the fractional rank of each region based on SDI. The Concentration Index can also be computed using the convenient regression method: CI = (2σ^2^_r_/µ) × β, where σ^2^_r_ is the variance of the fractional rank and β is the coefficient from regressing the health outcome on the fractional rank. Positive values indicate pro-rich inequality (higher burden in higher SDI regions), while negative values indicate pro-poor inequality (higher burden in lower SDI regions).

### Decomposition analysis

To further explore factors contributing to differences in global disease burden, we utilized decomposition analysis. This method allows for the disaggregation of overall health disparities into contributions from various factors, such as population growth, population aging, and epidemiological changes. The statistical approach follows the methodology described by Cheng et al., where changes in disease burden (number of cases or DALYs) between 1990 and each subsequent year are decomposed into three components using the following formula: ΔB = B_1_−B_0_ = (P_1_−P_0_) × R_0_ + P_0_ × (A_1_−A_0_) × R_0_ + P_0_ × A_0_ × (R_1_-R_0_) + interaction terms, where B represents disease burden, P represents total population size, A represents population age structure, R represents age-group-specific disease rates, and subscripts 0 and 1 denote the reference year (1990) and comparison year, respectively. The first component (P_1_−P_0_) × R_0_ quantifies the contribution of population growth, the second component P_0_ × (A_1_−A_0_) × R_0_ represents the contribution of population aging, and the third component P_0_ × A_0_ × (R_1_−R_0_) captures the contribution of epidemiological changes in disease rates. The relative contribution of each factor is calculated as the percentage of the total change attributable to that specific factor. This decomposition method has been extensively validated and applied in previous Global Burden of Disease studies to analyze mortality and disease burden trends across different populations and time periods.^27^

### BAPC model construction

The Bayesian Age-Period-Cohort (BAPC) prediction model is a statistical method based on a Bayesian framework that combines age, period, and birth cohort effects to forecast future trends in disease incidence, mortality, or other health indicators. We used the INLA framework within the BAPC package to predict disease burden from 2022 to 2040.

### Statistical analysis

All data processing, statistical analyses, and visualizations were performed using R version 4.4.1. The following packages were utilized: dplyr, tidyr, stringr, and arrow for data manipulation and analysis; ggplot2, ggmap, rgdal, RColorBrewer, patchwork, and ggrepel for data visualization; rgdal for geospatial analysis; and stats for statistical analysis.

## Results

### Global burden of schizophrenia among women of reproductive age: a 32-year analysis

**FIG. 1.**
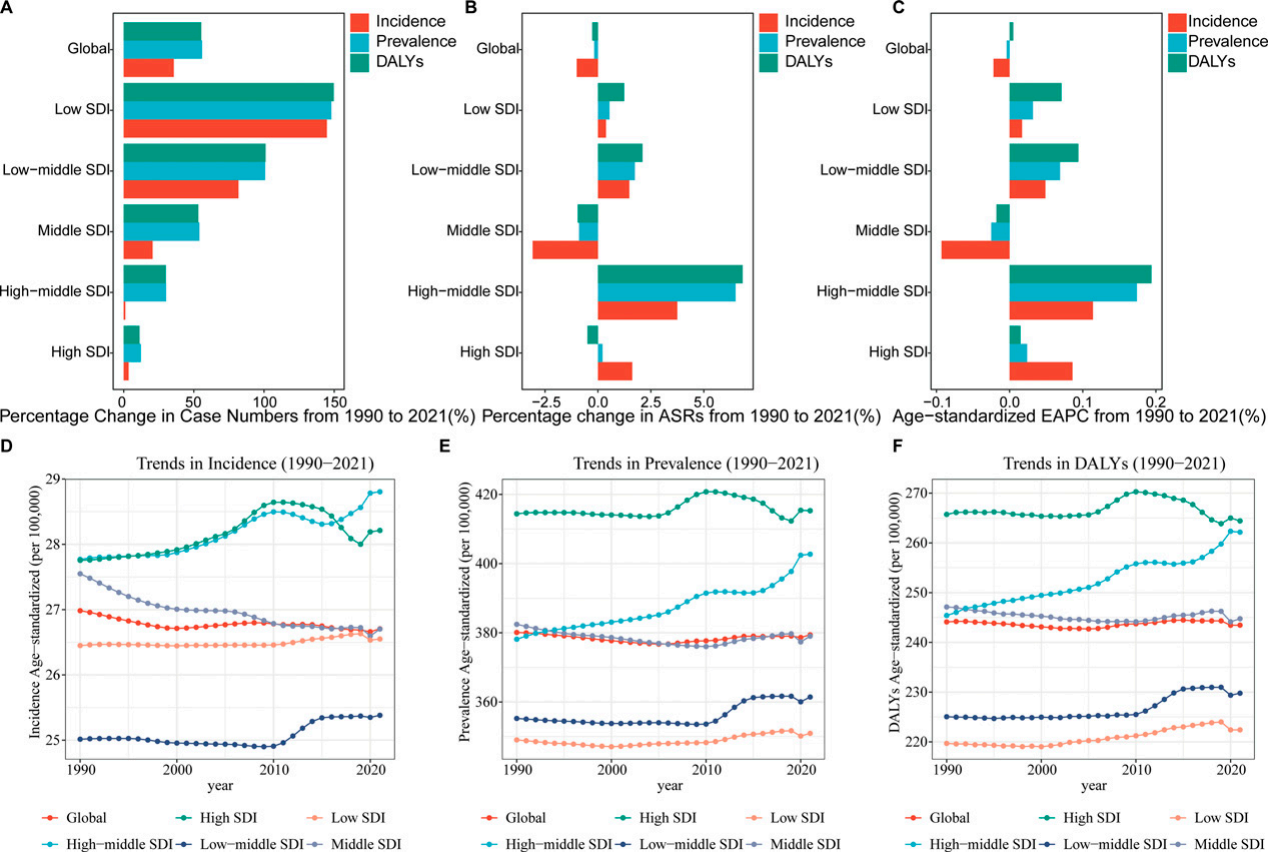
Global and sociodemographic Index (SDI)-specific trends in schizophrenia among women of reproductive age, 1990–2021. **(A)** Percentage change in case numbers of prevalence, incidence and disability-adjusted life years (DALYs) for Schizophrenia among women of reproductive age globally and in 5 SDI regions, 1990–2021. **(B)** Percentage change in age-standardized rates (ASR) of prevalence, incidence and DALYs for Schizophrenia among women of reproductive age globally and in 5 SDI regions, 1990–2021. **(C)** Estimated Annual Percentage Change (EAPC) in prevalence, incidence and DALYs for Schizophrenia among women of reproductive age globally and in 5 SDI regions, 1990–2021. **(D)** Trends in incidence rates of Schizophrenia among women of reproductive age globally and in 5 SDI regions, 1990–2021. **(E)** Trends in prevalence rates of Schizophrenia among women of reproductive age globally and in 5 SDI regions, 1990–2021. **(F)** Trends in DALY rates of Schizophrenia among women of reproductive age globally and in 5 SDI regions, 1990–2021.

**FIG. 2.**
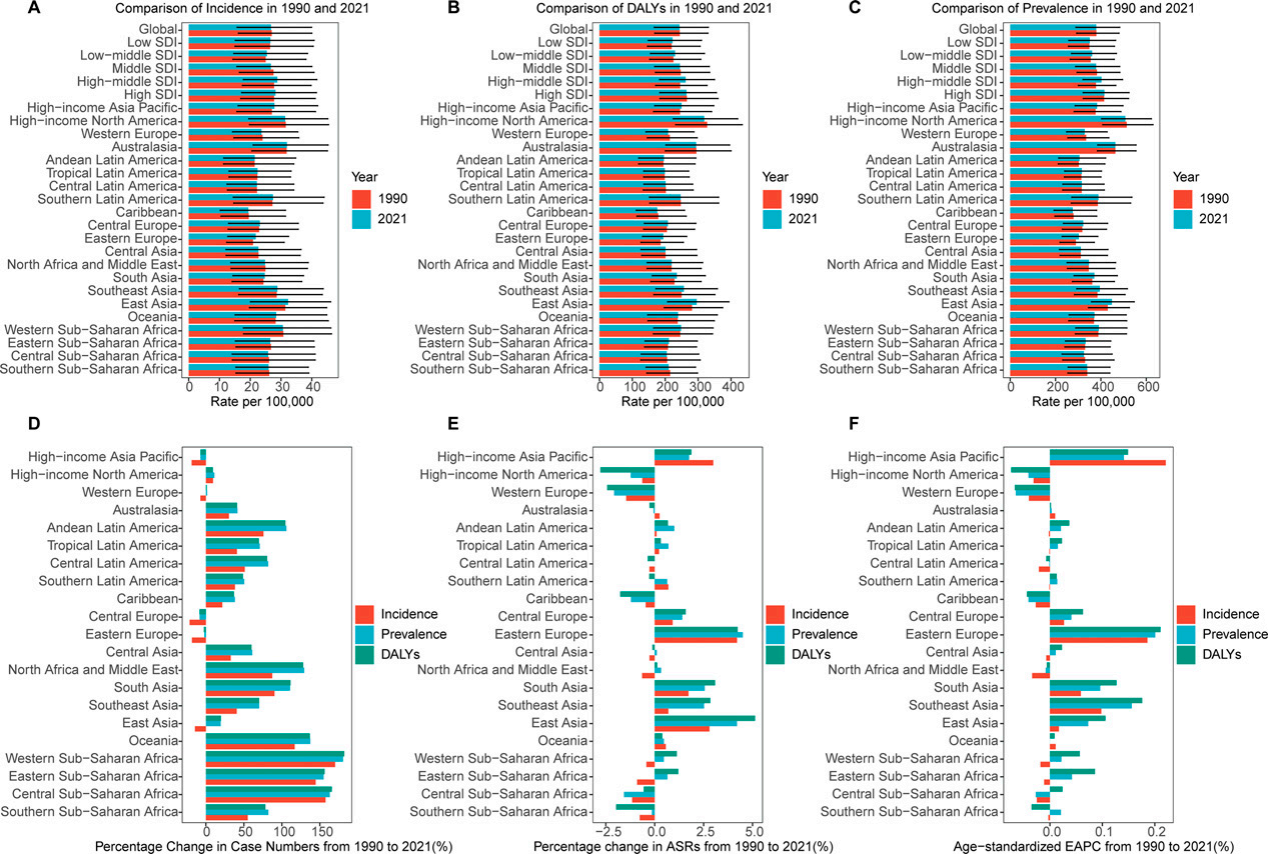
Regional comparisons of Schizophrenia burden among women of reproductive age, 1990 vs. 2021. **(A)** Comparison of incidence rates for Schizophrenia among women of reproductive age globally, in 5 SDI regions, and 21 GBD regions in 1990 and 2021. **(B)** Comparison of prevalence rates for Schizophrenia among women of reproductive age globally, in 5 SDI regions, and 21 GBD regions in 1990 and 2021. **(C)** Comparison of DALY rates for Schizophrenia among women of reproductive age globally, in 5 SDI regions, and 21 GBD regions in 1990 and 2021. **(D)** Percentage change in case numbers of prevalence, incidence and DALYs for Schizophrenia among women of reproductive age in 21 GBD regions, 1990–2021. **(E)** Percentage change in age-standardized rates (ASR) of prevalence, incidence and DALYs for Schizophrenia among women of reproductive age in 21 GBD regions, 1990–2021. **(F)** Estimated Annual Percentage Change (EAPC) in prevalence, incidence and DALYs for Schizophrenia among women of reproductive age in 21 GBD regions, 1990–2021.

Schizophrenia continues to pose a significant health challenge for women of reproductive age globally, with a notable increase in absolute numbers despite relatively stable ASR (Figs. 1A–F and 2A–F). From 1990 to 2021, the global incidence of schizophrenia in this population rose by 35.68%, from 378,287 to 513,255 cases (Table 1). However, the age-standardized incidence rate slightly decreased by 1.00%, from 26.98 to 26.71 per 100,000, with an estimated EAPC of −0.022 (95% CI: −0.029 to −0.014). Prevalence cases increased more substantially by 55.82%, reaching 7,541,989 in 2021, while the age-standardized prevalence rate remained nearly constant with a minimal decrease of 0.18% (Supplementary Table S1). The burden of disease, measured in DALYs, showed a similar trend, with a 55.27% increase in absolute numbers but a marginal decrease of 0.27% in age-standardized DALY rates (Supplementary Table S2). The EAPC for DALYs was slightly positive at 0.005 (95% CI: −0.004 to 0.013), indicating a small but non-significant upward trend.

**Table 1.** Global Incidence of Schizophrenia among Women of Reproductive Age in 1990 and 2021, with Trends from 1990 to 2021

Location	Incidence cases			Incidence rates			
	1990_numbers (95% UI)	2021_numbers (95% UI)	Percentage change in case (100%)	1990_per 100 000 (95% UI)	2021_per 100 000 (95% UI)	Percentage change in ASRs (100%)	EAPC (95% CI)
Global	378287 (227553, 559592)	513255 (302798, 767916)	35.68	26.98 (16.19, 40.01)	26.71 (15.76, 39.94)	−1.00	−0.022 (−0.029 to −0.014)
Low SDI	31214 (17710, 47752)	76403 (43471, 117297)	144.77	26.45 (15.05, 40.45)	26.55 (15.15, 40.74)	0.38	0.017 (0.012 to 0.022)
Low-middle SDI	72609 (41822, 110172)	132003 (76447, 200514)	81.80	25.01 (14.39, 38.08)	25.38 (14.69, 38.6)	1.48	0.049 (0.03 to 0.068)
Middle SDI	132302 (79545, 194526)	159547 (93310, 239328)	20.59	27.55 (16.46, 40.73)	26.7 (15.65, 39.99)	−3.09	−0.093 (−0.104 to −0.083)
High-middle SDI	79777 (50348, 114017)	80697 (49608, 117006)	1.15	27.77 (17.48, 39.78)	28.81 (17.78, 41.62)	3.75	0.114 (0.099 to 0.128)
High SDI	62087 (37803, 92044)	64248 (39289, 94639)	3.48	27.76 (16.9, 41.13)	28.21 (17.26, 41.5)	1.62	0.086 (0.057 to 0.115)
High-income Asia Pacific	12039 (6972, 18348)	9786 (5679, 14672)	−18.71	27.05 (15.64, 41.21)	27.86 (16.08, 41.91)	2.99	0.221 (0.148 to 0.294)
High-income North America	22705 (14153, 32884)	24839 (15409, 35946)	9.40	31.54 (19.77, 45.46)	31.34 (19.52, 45.19)	−0.63	−0.031 (−0.046 to −0.015)
Western Europe	22937 (14067, 34147)	21280 (12952, 31716)	−7.22	24.01 (14.7, 35.79)	23.66 (14.26, 35.47)	−1.46	−0.04 (−0.058 to −0.021)
Australasia	1692 (1087, 2404)	2205 (1433, 3157)	30.32	31.83 (20.54, 45.08)	31.91 (20.96, 45.3)	0.25	0.01 (0.004 to 0.017)
Andean Latin America	2157 (1140, 3438)	3791 (2011, 6134)	75.75	21.44 (11.36, 34.14)	21.46 (11.38, 34.77)	0.09	−0.002 (−0.009 to 0.005)
Tropical Latin America	9377 (5515, 13870)	13202 (7745, 19618)	40.79	22.33 (13.11, 33.09)	22.38 (13.12, 33.26)	0.22	−0.001 (−0.01 to 0.009)
Central Latin America	9963 (5654, 15312)	15055 (8472, 23176)	51.11	22.22 (12.6, 34.14)	22.16 (12.46, 34.13)	−0.27	−0.021 (−0.027 to −0.014)
Southern Latin America	3406 (1829, 5470)	4710 (2502, 7544)	38.29	27.2 (14.64, 43.63)	27.39 (14.5, 43.97)	0.70	−0.001 (−0.018 to 0.016)
Caribbean	1912 (1025, 3088)	2325 (1226, 3765)	21.60	19.51 (10.5, 31.48)	19.42 (10.25, 31.45)	−0.46	−0.027 (−0.042 to −0.012)
Central Europe	6823 (3820, 10607)	5361 (3019, 8235)	−21.43	22.89 (12.81, 35.61)	23.1 (12.98, 35.59)	0.92	0.027 (0.019 to 0.036)
Eastern Europe	11466 (6746, 16992)	9358 (5426, 14023)	−18.38	20.91 (12.33, 31.02)	21.79 (12.71, 32.45)	4.21	0.186 (0.149 to 0.222)
Central Asia	4120 (2199, 6572)	5467 (2917, 8794)	32.69	22.71 (12.17, 36.21)	22.65 (12.1, 36.51)	−0.26	−0.007 (−0.014 to 0)
North Africa and Middle East	41878 (23100, 64924)	78422 (43428, 122862)	87.26	24.93 (13.78, 38.58)	24.77 (13.71, 38.85)	−0.64	−0.034 (−0.039 to −0.03)
South Asia	131592 (76987, 197678)	250381 (146200, 377004)	90.27	24.3 (14.14, 36.73)	24.72 (14.4, 37.31)	1.73	0.059 (0.029 to 0.089)
Southeast Asia	36922 (20997, 55906)	51865 (29701, 78697)	40.47	28.61 (16.28, 43.42)	28.81 (16.5, 43.71)	0.70	0.098 (0.063 to 0.133)
East Asia	112065 (71228, 158701)	95909 (58924, 138302)	−14.42	31.41 (19.75, 44.84)	32.29 (20.09, 46.12)	2.80	0.017 (−0.011 to 0.045)
Oceania	469 (247, 755)	1017 (538, 1605)	116.84	28.25 (14.99, 45.37)	28.41 (15.09, 44.84)	0.57	0.011 (−0.01 to 0.031)
Western Sub-Saharan Africa	14244 (8203, 21537)	38471 (22228, 58100)	170.09	30.76 (17.85, 46.4)	30.63 (17.78, 46.2)	−0.42	−0.018 (−0.024 to −0.012)
Eastern Sub-Saharan Africa	12167 (6952, 18630)	29744 (16919, 45778)	144.46	26.75 (15.39, 40.85)	26.51 (15.17, 40.69)	−0.90	−0.011 (−0.019 to −0.003)
Central Sub-Saharan Africa	3400 (1838, 5380)	8751 (4748, 14001)	157.38	26.1 (14.2, 41.1)	25.8 (14.12, 41.09)	−1.15	−0.025 (−0.034 to −0.015)
Southern Sub-Saharan Africa	3688 (2162, 5493)	5717 (3374, 8542)	55.02	26.18 (15.38, 39.01)	25.98 (15.34, 38.86)	−0.76	−0.003 (−0.014 to 0.007)

95% CI, 95% confidence interval; ASR, age-standardized rates; SDI, sociodemographic Index; UI, Uncertainty Interval.

### Disparities in schizophrenia burden among women of reproductive age across sociodemographic index regions

The burden of schizophrenia among women of reproductive age exhibits significant disparities across different SDI regions, with lower SDI areas experiencing the most substantial increases over the past three decades (Figs. 1A–F and 2A–F). From 1990 to 2021, low SDI regions saw the highest percentage increase in incidence cases (144.77%), prevalence cases (147.92%), and DALYs (149.63%) (Table 1 and Supplementary Tables S1 and S2). Conversely, high SDI regions showed more modest increases of 3.48%, 12.21%, and 11.27% in incidence, prevalence, and DALYs, respectively. The age-standardized incidence rates remained relatively stable across all SDI regions, with slight increases in high-middle SDI (3.75%) and high SDI (1.62%) areas, and a decrease in middle SDI regions (−3.09%). Notably, the EAPC incidence rates varied considerably, ranging from −0.093 (95% CI: −0.104 to −0.083) in middle SDI regions to 0.114 (95% CI: 0.099 to 0.128) in high-middle SDI areas. Age-standardized prevalence rates showed the most significant increase in high-middle SDI regions (6.49%), with an EAPC of 0.174 (95% CI: 0.157 to 0.191). Similarly, age-standardized DALY rates increased most in high-middle SDI regions (6.83%), with an EAPC of 0.194 (95% CI: 0.180 to 0.208).

### Regional disparities in schizophrenia burden among women of reproductive age: a global perspective

The burden of schizophrenia among women of reproductive age varies significantly across global regions, with notable disparities in ASRs, prevalence rates, and DALY rates (Figs. 1A–F and 2A–F). Sub-Saharan African regions experienced notable changes in schizophrenia burden from 1990 to 2021. Western Sub-Saharan Africa showed a slight decrease in the ASR of 0.42% (Table 1). Conversely, High-income Asia Pacific experienced increases in ASRs despite decreases in absolute numbers. Notably, East Asia showed increases in ASRs across all measures, with the highest EAPC in prevalence (0.073, 95% CI: 0.045 to 0.101) and DALYs (0.106, 95% CI: 0.081 to 0.131). South Asia demonstrated positive EAPCs, indicating a growing age-adjusted burden. Eastern Europe exhibited the highest positive EAPC in prevalence (0.201, 95% CI: 0.159 to 0.243) and DALYs (0.211, 95% CI: 0.168 to 0.255). (Crude values for all regions are provided in Supplementary Tables S1, S2 and S3.).

### Heterogeneous trends in schizophrenia burden among women of reproductive age across 204 countries

**FIG. 3.**
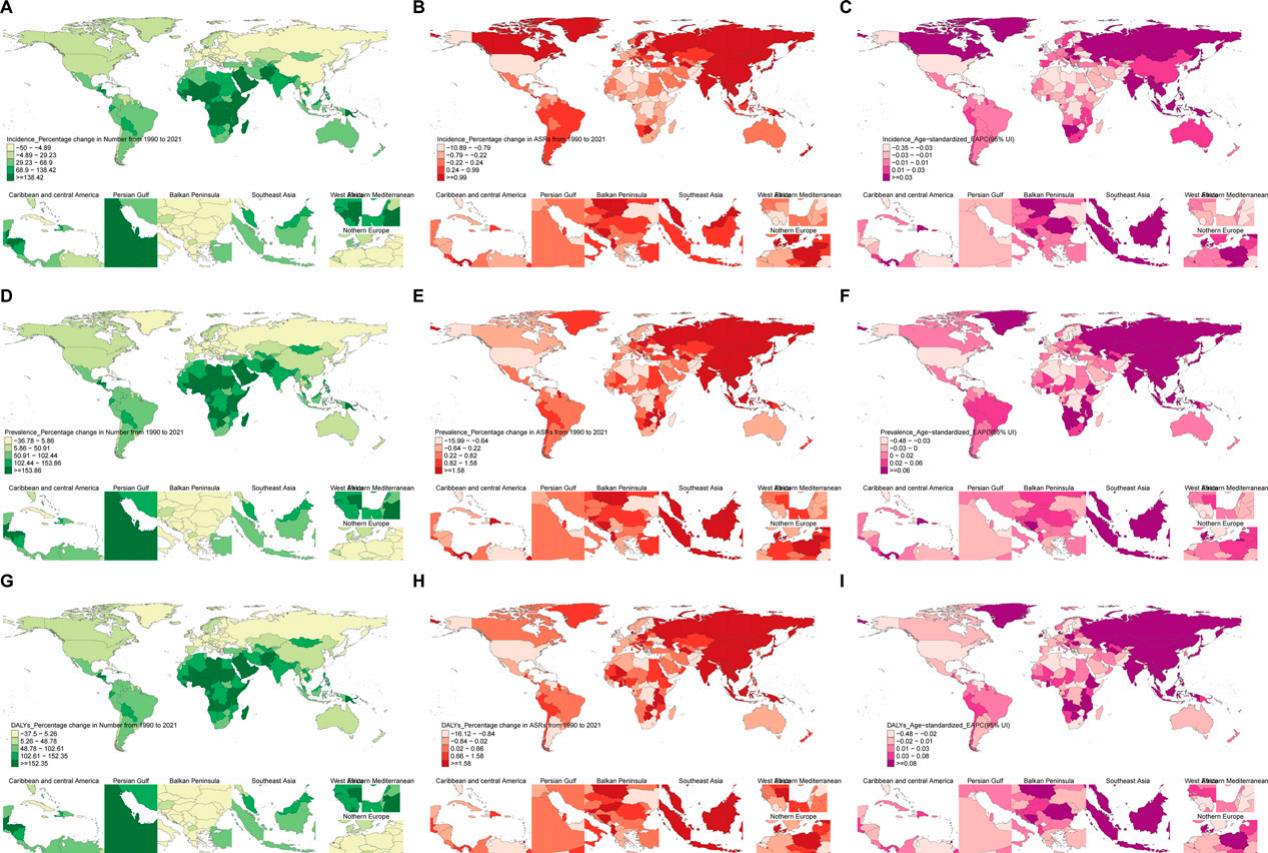
Global geographical distribution of changes in Schizophrenia burden among women of reproductive age, 1990–2021. **(A)** Percentage change in incidence numbers of Schizophrenia among women of reproductive age in 204 countries/territories, 1990–2021. **(B)** Percentage change in age-standardized incidence rates of Schizophrenia among women of reproductive age in 204 countries/territories, 1990–2021. **(C)** EAPC in incidence of Schizophrenia among women of reproductive age in 204 countries/territories, 1990–2021. **(D)** Percentage change in prevalence numbers of Schizophrenia among women of reproductive age in 204 countries/territories, 1990–2021. **(E)** Percentage change in age-standardized prevalence rates of Schizophrenia among women of reproductive age in 204 countries/territories, 1990–2021. **(F)** EAPC in prevalence of Schizophrenia among women of reproductive age in 204 countries/territories, 1990–2021. **(G)** Percentage change in DALY numbers of Schizophrenia among women of reproductive age in 204 countries/territories, 1990–2021. **(H)** Percentage change in age-standardized DALY rates of Schizophrenia among women of reproductive age in 204 countries/territories, 1990–2021. **(I)** EAPC in DALYs of Schizophrenia among women of reproductive age in 204 countries/territories, 1990–2021.

The burden of schizophrenia among women of reproductive age exhibits substantial heterogeneity across countries, reflecting diverse epidemiological patterns and health care contexts globally (Fig. 3A–1). While acknowledging the inherent variability in data sources across countries, this country-level analysis provides valuable insights into the global diversity of schizophrenia burden trends. Several countries demonstrated particularly notable patterns: Georgia showed consistent increases across all age-standardized measures, with incidence rates rising by 115.61% (from 32.47 to 70.01 per 100,000) and an EAPC of 2.5 (95% CI: 2.42 to 2.57) (Supplementary Table S3). Denmark also exhibited increases across all metrics, with prevalence cases rising by 34.62% and an EAPC of 1.51 (95% CI: 1.23 to 1.79). Conversely, the United Kingdom experienced declines across all measures, with prevalence rates decreasing by 5.51% and an EAPC of −0.48 (95% CI: −0.64 to −0.32). These country-specific variations complement the regional analyses and highlight the importance of tailored national strategies for schizophrenia prevention and management among women of reproductive age.

### Age-group-specific trends in schizophrenia burden among women of reproductive age: A global analysis from 1990 to 2021

**FIG. 4.**
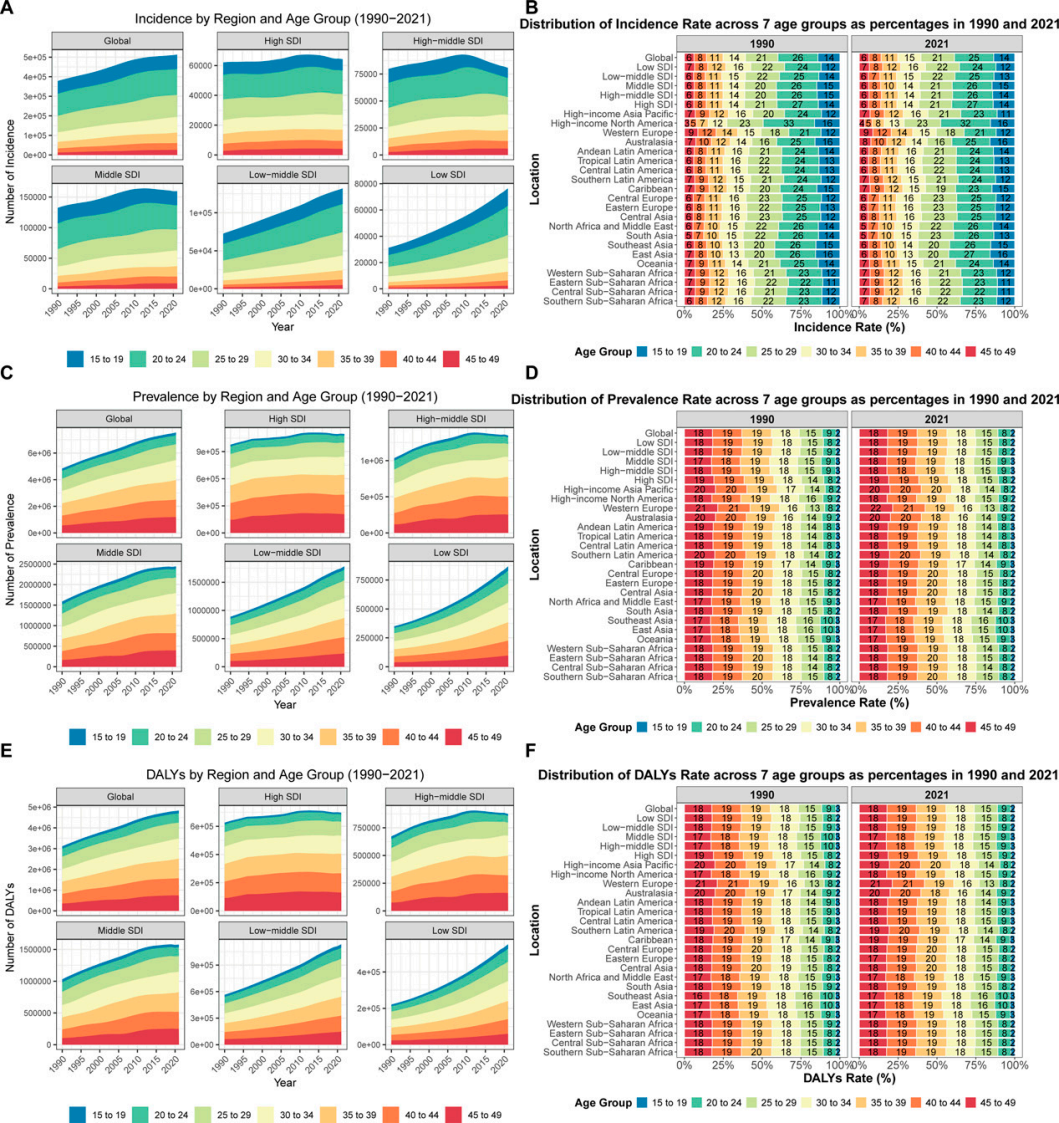
Age-specific trends and distributions of Schizophrenia burden among women of reproductive age, 1990–2021. **(A)** Trends in incidence across 7 age groups (15–49 years, 5-year intervals) for schizophrenia among women of reproductive age globally and in 5 SDI regions, 1990–2021. **(B)** Percentage distribution of incident cases across 7 age groups for schizophrenia among women of reproductive age globally, in 5 SDI regions, and 21 GBD regions in 1990 and 2021. **(C)** Trends in prevalence across 7 age groups (15–49 years, 5-year intervals) for schizophrenia among women of reproductive age globally and in 5 SDI regions, 1990–2021. **(D)** Percentage distribution of prevalent cases across 7 age groups for schizophrenia among women of reproductive age globally, in 5 SDI regions, and 21 GBD regions in 1990 and 2021. **(E)** Trends in DALYs across 7 age groups (15–49 years, 5-year intervals) for schizophrenia among women of reproductive age globally and in 5 SDI regions, 1990–2021. **(F)** Percentage distribution of DALYs across 7 age groups for schizophrenia among women of reproductive age globally, in 5 SDI regions, and 21 GBD regions in 1990 and 2021.

**FIG. 5.**
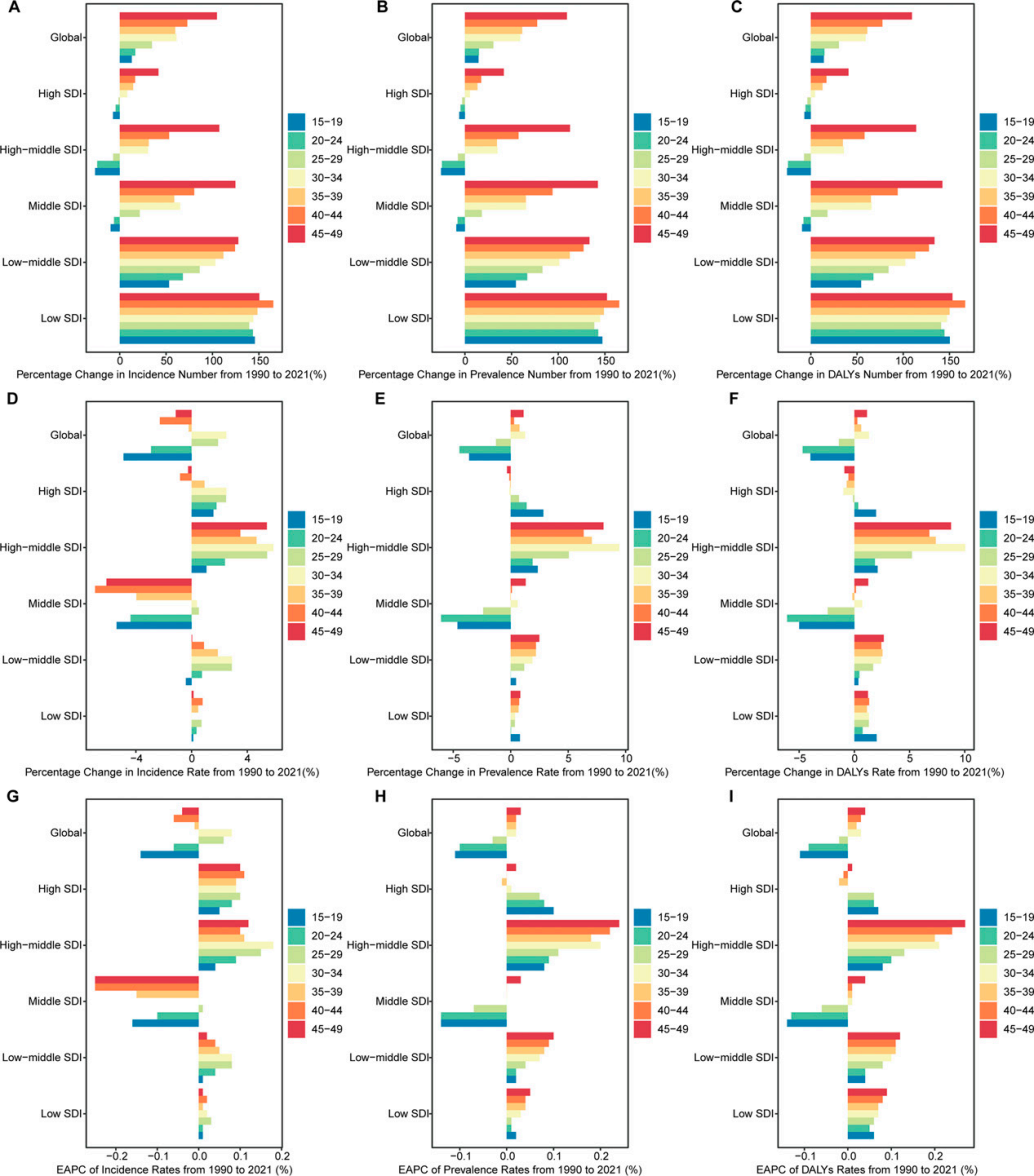
Age-specific changes in schizophrenia burden among women of reproductive age, 1990–2021. **(A)** Percentage change in incidence numbers across 7 age groups for schizophrenia among women of reproductive age globally and in 5 SDI regions, 1990–2021. **(B)** Percentage change in prevalence numbers across 7 age groups for schizophrenia among women of reproductive age globally and in 5 SDI regions, 1990–2021. **(C)** Percentage change in DALY numbers across 7 age groups for schizophrenia among women of reproductive age globally and in 5 SDI regions, 1990–2021. **(D)** Percentage change in incidence rates across 7 age groups for schizophrenia among women of reproductive age globally and in 5 SDI regions, 1990–2021. **(E)** Percentage change in prevalence rates across 7 age groups for schizophrenia among women of reproductive age globally and in 5 SDI regions, 1990–2021. **(F)** Percentage change in DALY rates across 7 age groups for schizophrenia among women of reproductive age globally and in 5 SDI regions, 1990–2021. **(G)** EAPC in incidence across 7 age groups for schizophrenia among women of reproductive age globally and in 5 SDI regions, 1990–2021. **(H)** EAPC in prevalence across 7 age groups for schizophrenia among women of reproductive age globally and in 5 SDI regions, 1990–2021. **(I)** EAPC in DALYs across 7 age groups for schizophrenia among women of reproductive age globally and in 5 SDI regions, 1990–2021.

The global burden of schizophrenia among women of reproductive age demonstrated distinct age-group-specific patterns across five-year age groups from 1990 to 2021 (Figs. 4A–F and 5A–I). A clear age gradient was observed, with older reproductive age groups experiencing more substantial increases in burden compared to younger cohorts. The 45–49 age group exhibited the most pronounced increase in incidence, with cases rising from 356.88 thousand (95% UI: 253.07–457.25) to 747.43 thousand (95% UI: 532.29–962.45), representing a 109.44% increase (Table 2). The age-standardized incidence rate for this group also increased modestly from 313.6 (95% UI: 222.38–401.8) to 317.19 (95% UI: 225.89–408.43) per 100,000, with an EAPC of 0.04 (95% CI: 0.03 to 0.06). In contrast, younger reproductive age groups showed more modest changes or declining trends. The 15–19 age group demonstrated a 14.11% increase in incidence cases, from 116.58 thousand (95% UI: 70.66–178.83) to 133.03 thousand (95% UI: 80.38–206.01), while the age-standardized incidence rate decreased by 3.97% from 45.62 (95% UI: 27.65–69.98) to 43.81 (95% UI: 26.47–67.85) per 100,000, with a negative EAPC of −0.11 (95% CI: −0.14 to −0.08). This age-group-specific gradient was consistently observed across prevalence and DALY measures, indicating that the burden of schizophrenia has disproportionately increased among older women of reproductive age globally.

**Table 2.** Age-Specific Incidence of Schizophrenia Among Women of Reproductive Age in 1990 and 2021, with Trends in Age Patterns from 1990 to 2021

Location	Age (years)	Incidence cases			Incidence rates			
		1990_thousands (95% UI)	2021_thousands (95% UI)	Percentage change in case (100%)	1990_per 100 000 (95% UI)	2021_per 100 000 (95% UI)	Percentage change in ASRs (100%)	EAPC (95% CI)
Global	45 to 49	356.88 (253.07–457.25)	747.43 (532.29–962.45)	109.44	313.6 (222.38–401.8)	317.19 (225.89–408.43)	1.14	0.04 (0.03 to 0.06)
Global	40 to 44	465.3 (331.84–598.5)	825.47 (585.85–1066.97)	77.41	331.82 (236.64–426.81)	332.73 (236.14–430.07)	0.27	0.03 (0.01 to 0.04)
Global	35 to 39	585.45 (408.26–764.75)	943.41 (656.07–1246.16)	61.14	337.53 (235.37–440.9)	339.6 (236.16–448.58)	0.61	0.02 (0.02 to 0.03)
Global	30 to 34	606.24 (422.02–813.32)	965.84 (668.39–1300.43)	59.32	318.89 (221.99–427.82)	323.1 (223.59–435.03)	1.32	0.03 (0.01 to 0.05)
Global	25 to 29	589.72 (404.75–830.59)	768.76 (511.63–1085.9)	30.36	267.93 (183.9–377.37)	264.19 (175.83–373.18)	−1.40	−0.02 (−0.05 to 0)
Global	20 to 24	394.81 (248.07–595.04)	452.76 (283.85–699.36)	14.68	161.72 (101.61–243.73)	154.13 (96.63–238.08)	−4.69	−0.09 (−0.13 to −0.06)
Global	15 to 19	116.58 (70.66–178.83)	133.03 (80.38–206.01)	14.11	45.62 (27.65–69.98)	43.81 (26.47–67.85)	−3.97	−0.11 (−0.14 to −0.08)
High SDI	45 to 49	91.75 (65.01–118.58)	129.21 (91.86–165.31)	40.83	363.13 (257.28–469.32)	359.86 (255.83–460.4)	−0.90	0.01 (−0.02 to 0.04)
High SDI	40 to 44	116.75 (83.73–149.27)	136.54 (97.43–173.96)	16.95	373.93 (268.17–478.09)	371.93 (265.39–473.85)	−0.54	−0.01 (−0.03 to 0.02)
High SDI	35 to 39	123.96 (86.43–162.79)	139.64 (98–182.8)	12.65	370.7 (258.46–486.81)	368.13 (258.37–481.91)	−0.70	−0.02 (−0.04 to 0)
High SDI	30 to 34	122.96 (86.58–165.16)	128.43 (90.55–170.93)	4.45	346.52 (244.01–465.43)	343.09 (241.89–456.62)	−0.99	0 (−0.02 to 0.03)
High SDI	25 to 29	101.86 (70.8–145.72)	98.08 (67.82–137.06)	−3.71	284.15 (197.5–406.51)	283.79 (196.25–396.6)	−0.13	0.06 (0.03 to 0.09)
High SDI	20 to 24	55.36 (34.14–84.75)	52.15 (32.55–79.19)	−5.79	164.85 (101.68–252.39)	165.43 (103.24–251.21)	0.35	0.06 (0.01 to 0.11)
High SDI	15 to 19	13.05 (7.75–20.73)	12.15 (7.23–18.76)	−6.92	40.95 (24.33–65.03)	41.76 (24.85–64.5)	1.96	0.07 (0.03 to 0.11)
High-middle SDI	45 to 49	75.01 (53.96–95.17)	160.48 (117.17–203.5)	113.94	305.95 (220.08–388.16)	332.75 (242.94–421.96)	8.76	0.27 (0.23 to 0.31)
High-middle SDI	40 to 44	100.17 (70.77–129.96)	158.38 (113.53–200.86)	58.11	325.81 (230.19–422.72)	347.93 (249.42–441.26)	6.79	0.24 (0.2 to 0.28)
High-middle SDI	35 to 39	132.69 (92.94–171.49)	178.74 (125.87–233.25)	34.70	335.96 (235.31–434.19)	360.72 (254.02–470.72)	7.37	0.2 (0.16 to 0.25)
High-middle SDI	30 to 34	133.03 (93.68–176.64)	180.54 (126.86–236.84)	35.71	318.66 (224.39–423.11)	350.63 (246.38–459.96)	10.03	0.21 (0.17 to 0.26)
High-middle SDI	25 to 29	125.86 (89.06–172.76)	116.69 (81.38–161.51)	−7.29	275.13 (194.69–377.66)	289.48 (201.89–400.67)	5.22	0.13 (0.09 to 0.17)
High-middle SDI	20 to 24	82.6 (52.91–117.82)	62.22 (39.42–92.7)	−24.68	171.58 (109.91–244.75)	174.79 (110.73–260.42)	1.87	0.1 (0.06 to 0.15)
High-middle SDI	15 to 19	22.96 (14.06–34.79)	17.05 (10.5–25.59)	−25.76	48.48 (29.68–73.45)	49.5 (30.51–74.32)	2.11	0.08 (0.04 to 0.12)
Low SDI	45 to 49	23.75 (16.85–31.15)	60.14 (42.09–79.16)	153.24	286.07 (202.94–375.17)	289.59 (202.69–381.16)	1.23	0.09 (0.07 to 0.1)
Low SDI	40 to 44	30.17 (21.17–39.82)	80.54 (56.5–106.63)	166.95	304.3 (213.48–401.62)	308.36 (216.32–408.22)	1.33	0.08 (0.06 to 0.09)
Low SDI	35 to 39	39.96 (27.25–53.69)	99.95 (67.98–133.95)	150.15	310.11 (211.53–416.66)	313.66 (213.32–420.35)	1.14	0.07 (0.06 to 0.09)
Low SDI	30 to 34	44.3 (29.37–61.13)	109.53 (73.84–150.3)	147.25	291.22 (193.08–401.9)	295.07 (198.92–404.93)	1.32	0.07 (0.06 to 0.08)
Low SDI	25 to 29	43.79 (27.66–63.33)	105.49 (67.59–154.28)	140.90	236.41 (149.35–341.88)	239.49 (153.45–350.26)	1.30	0.06 (0.05 to 0.07)
Low SDI	20 to 24	29.57 (17.75–47.25)	72.33 (42.87–115.18)	144.59	136.15 (81.73–217.54)	137.17 (81.3–218.42)	0.75	0.05 (0.04 to 0.06)
Low SDI	15 to 19	9.66 (5.07–16.09)	24.18 (13.16–40.71)	150.31	38.44 (20.18–64.02)	39.21 (21.35–66.03)	2.01	0.06 (0.05 to 0.07)
Low-middle SDI	45 to 49	62.57 (44.09–80.29)	146.31 (103.02–190.04)	133.83	288.27 (203.12–369.93)	295.95 (208.39–384.41)	2.66	0.12 (0.09 to 0.15)
Low-middle SDI	40 to 44	79.69 (56.69–102.99)	181.49 (126.78–237.17)	127.75	307.14 (218.5–396.94)	314.64 (219.8–411.16)	2.44	0.11 (0.09 to 0.14)
Low-middle SDI	35 to 39	100.88 (69.58–132.03)	215.02 (149.06–290.46)	113.15	314.9 (217.19–412.16)	322.92 (223.86–436.21)	2.55	0.11 (0.08 to 0.13)
Low-middle SDI	30 to 34	111.48 (76.62–151.28)	225.4 (155.5–309.07)	102.18	297.73 (204.62–404.02)	304.97 (210.4–418.19)	2.43	0.1 (0.08 to 0.12)
Low-middle SDI	25 to 29	110.37 (72.91–159.15)	203.19 (134.83–295.16)	84.09	245.92 (162.46–354.59)	250.09 (165.95–363.29)	1.70	0.08 (0.05 to 0.1)
Low-middle SDI	20 to 24	75.03 (46.16–117.62)	125.75 (75–202.53)	67.59	143.86 (88.5–225.51)	144.52 (86.2–232.77)	0.46	0.04 (0.03 to 0.06)
Low-middle SDI	15 to 19	24.2 (14.16–39.18)	37.37 (21.16–59.33)	54.42	41.19 (24.1–66.68)	41.34 (23.41–65.64)	0.36	0.04 (0.02 to 0.06)
Middle SDI	45 to 49	103.48 (73.19–133.38)	250.76 (178.74–326.27)	142.32	305.32 (215.95–393.54)	309.15 (220.37–402.25)	1.26	0.04 (0.02 to 0.06)
Middle SDI	40 to 44	138.11 (98.79–179.71)	267.92 (190.5–349.87)	93.99	326.84 (233.8–425.3)	327.32 (232.75–427.45)	0.15	0.01 (0 to 0.03)
Middle SDI	35 to 39	187.48 (131.93–243.64)	309.42 (214.67–408.29)	65.04	338.22 (238.01–439.54)	337.61 (234.23–445.5)	−0.18	0.01 (−0.01 to 0.02)
Middle SDI	30 to 34	193.97 (134.95–260.7)	321.31 (223.6–430.94)	65.65	323.08 (224.77–434.22)	325.37 (226.42–436.38)	0.71	0.01 (−0.01 to 0.03)
Middle SDI	25 to 29	207.4 (142.59–291.36)	244.79 (164.5–346.61)	18.03	276.87 (190.36–388.96)	270.19 (181.57–382.57)	−2.41	−0.06 (−0.09 to −0.03)
Middle SDI	20 to 24	151.97 (93.29–229.91)	140.01 (88.36–214.27)	−7.87	172.04 (105.62–260.28)	161.55 (101.95–247.23)	−6.10	−0.13 (−0.17 to −0.1)
Middle SDI	15 to 19	46.62 (28.31–71.8)	42.19 (25.33–65.15)	−9.49	50.56 (30.71–77.87)	48.03 (28.83–74.17)	−4.99	−0.14 (−0.18 to −0.1)

EAPC, Estimated Annual Percentage Change.

### Divergent age-group-specific trends in schizophrenia burden among women of reproductive age across sociodemographic index levels

The burden of schizophrenia among women of reproductive age exhibited distinct patterns across the five SDI regions from 1990 to 2021, as evidenced by trends in incidence (Table 2), prevalence (Supplementary Table S6), and DALYs (Supplementary Table S7). High SDI regions demonstrated the most modest changes. In the 45–49 age group, which experienced the largest increases, incidence cases rose by 40.83%, prevalence cases by 41.65%, and DALY cases by 40.83% (Table 2 and Supplementary Tables S6 and S7). However, the corresponding rates showed slight decreases, with DALY rates declining by 0.90% and a low EAPC of 0.01 (95% CI: −0.02 to 0.04) (Supplementary Table S7). High-middle SDI regions exhibited the most rapid increases, particularly in older age groups. For the 45–49 age group, incidence cases increased by 113.94%, prevalence cases by 112.59%, and DALY cases by 113.94% (Tables 2 and Supplementary Tables S6 and S7). The DALY rate for this group increased by 8.76%, with the highest EAPC of 0.27 (95% CI: 0.23 to 0.31) across all regions and metrics (Supplementary Table S7). Middle SDI regions presented a mixed pattern. Older age groups (35–49 years) showed significant increases, with incidence cases rising between 65.04% and 142.32%, prevalence cases between 61.39% and 142.43%, and DALY cases between 65.04% and 142.32% (Table 2 and Supplementary Tables S6 and S7). However, younger groups (15–29 years) demonstrated decreases or minimal increases across all metrics. Low-middle SDI regions showed substantial increases, particularly in older age groups. The 45–49 age group saw increases of 133.83% in incidence, 133.45% in prevalence, and 133.83% in DALY cases (Table 2 and Supplementary Tables S6 and S7). The DALY rate for this group increased by 2.66%, with an EAPC of 0.12 (95% CI: 0.09 to 0.15) (Supplementary Table S7). Low SDI regions consistently experienced the most substantial increases across all metrics and age groups. In the 40–44 age group, which saw the highest rises, incidence, prevalence, and DALY cases all increased by 166.95% (Table 2 and Supplementary Tables S6 and S7). The corresponding DALY rate increased by 1.33%, with an EAPC of 0.08 (95% CI: 0.06 to 0.09) (Supplementary Table S7).

### Relationship between schizophrenia burden and sociodemographic development from 1990 to 2021

**FIG. 6.**
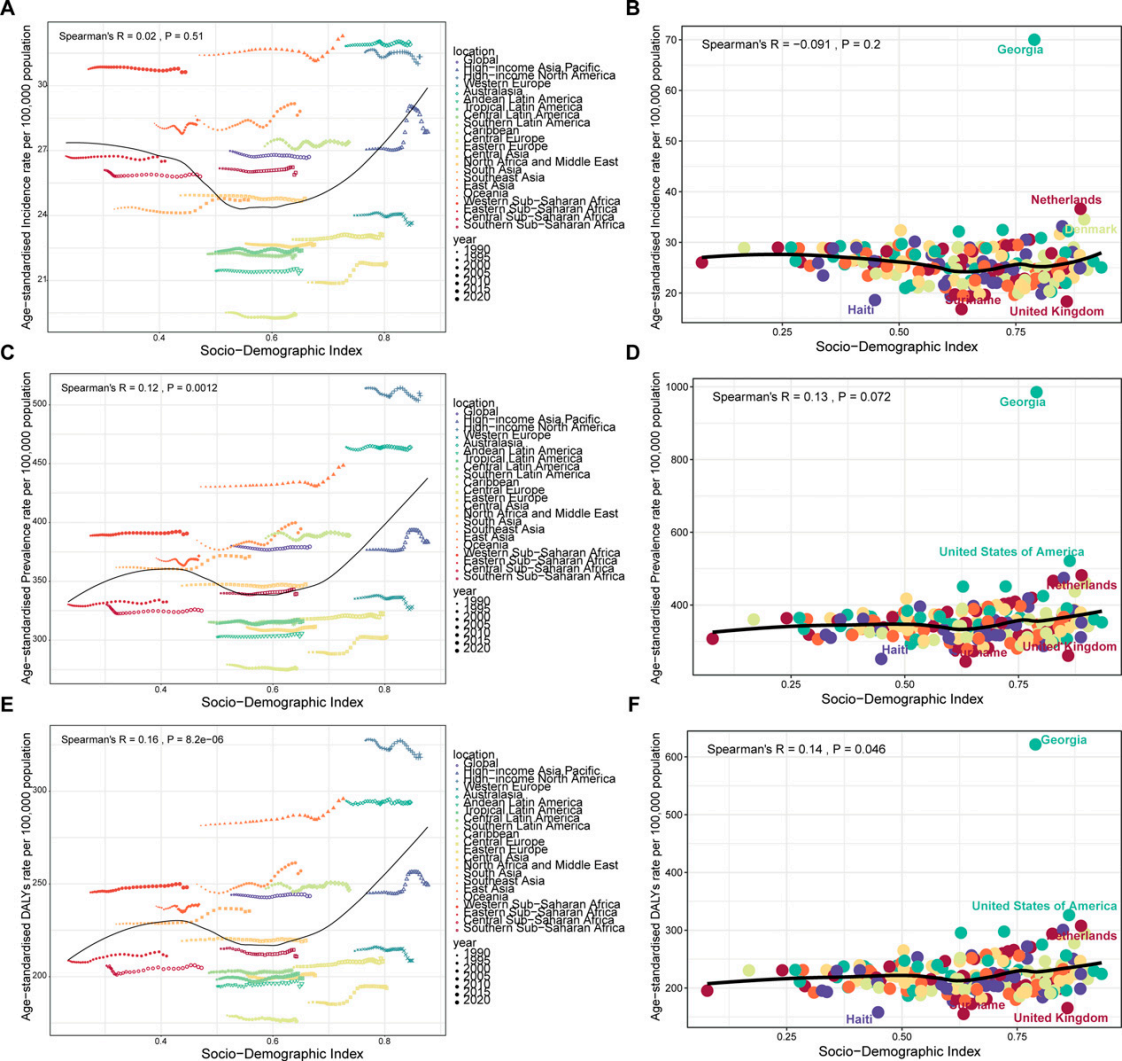
Relationship between SDI and schizophrenia burden among women of reproductive age. **(A)** Association between SDI and incidence rates (per 100,000) of schizophrenia among women of reproductive age in 21 GBD regions. **(B)** Association between SDI and incidence rates (per 100,000) of schizophrenia among women of reproductive age in 204 GBD countries. **(C)** Association between SDI and prevalence rates (per 100,000) of schizophrenia among women of reproductive age in 21 GBD regions. **(D)** Association between SDI and prevalence rates (per 100,000) of schizophrenia among women of reproductive age in 204 GBD countries. **(E)** Association between SDI and DALY rates (per 100,000) of schizophrenia among women of reproductive age in 21 GBD regions. **(F)** Association between SDI and DALY rates (per 100,000) of schizophrenia among women of reproductive age in 204 GBD countries.

The relationship between schizophrenia burden and sociodemographic development exhibited complex nonlinear patterns from 1990 to 2021 (Fig. 6A–F). Analysis across 21 regions revealed that age-standardized schizophrenia burden generally decreased when SDI values ranged between 0.4 and 0.6, suggesting potential benefits of moderate sociodemographic development on population mental health. However, when SDI values exceeded 0.6, the burden showed an increasing trend, reaching peak levels around an SDI of 0.8. This U-shaped relationship indicates that very high levels of sociodemographic development may be associated with increased schizophrenia burden, possibly reflecting urbanization effects, lifestyle changes, or improved diagnostic capacity. Regional variations from this overall pattern were observed: Australasia and high-income North America demonstrated age-standardized burden levels above the global SDI-burden curve, while the Caribbean and Eastern Europe showed levels below this curve. Similarly, country-level analysis revealed that Georgia, the Netherlands, and Denmark exhibited schizophrenia burden above expected levels based on their SDI values, with Georgia showing particularly elevated burden. Conversely, Haiti, Suriname, and the United Kingdom demonstrated burden levels below expectations relative to their sociodemographic development status.

### Decomposition analysis reveals divergent drivers of schizophrenia burden across global and SDI-specific contexts

**FIG. 7.**
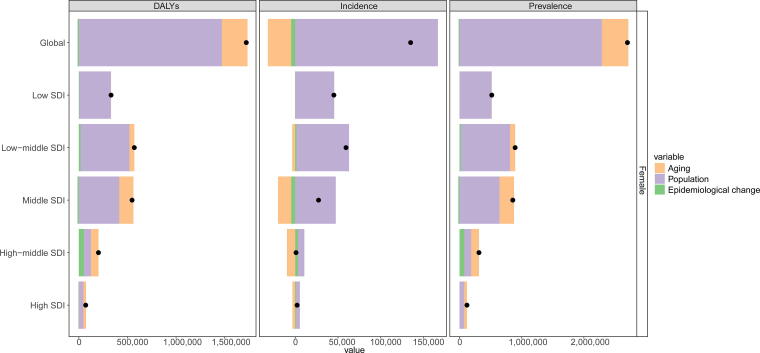
Decomposition analysis of incidence, prevalence and DALYs for schizophrenia among women of reproductive age globally and in 5 SDI regions.

Globally, the burden of schizophrenia among women of reproductive age increased substantially from 1990 to 2021, with population growth emerging as the primary driver (Fig. 7). As shown in Supplementary Table S8, population growth accounted for 85.4% of the increase in DALYs, 84.71% of the rise in prevalence, and 123.68% of the increase in incidence. Aging also played a significant role, contributing to 15.33% of the DALY increase and 15.8% of the prevalence increase. Interestingly, epidemiological changes had a slight mitigating effect, reducing DALYs by 0.73%, prevalence by 0.51%, and incidence by 3.51%.

The decomposition analysis revealed distinct patterns across SDI regions (Supplementary Table S8). In high SDI regions, aging was a major contributor, accounting for 39.41% of the DALY increase and 37.84% of the prevalence rise, while population growth contributed 65.61% and 60.8%, respectively. Middle SDI regions showed a mixed pattern, with population growth driving 75.89% of the DALY increase, but epidemiological changes reducing incidence by 16.79%. High-middle SDI regions uniquely demonstrated positive epidemiological changes, contributing 25.73% to the DALY increase and 24.36% to the prevalence rise. Low-middle SDI regions were predominantly influenced by population growth, accounting for 88.11% of the DALY increase and 88.32% of the prevalence rise. In low SDI regions, population growth was overwhelmingly the main driver, responsible for 98.11% of the DALY increase, 98.9% of the prevalence rise, and 100.41% of the incidence increase, highlighting the critical need for population-focused interventions in these areas.

### Health inequality analysis of schizophrenia among women of reproductive age: 1990–2021

**FIG. 8.**
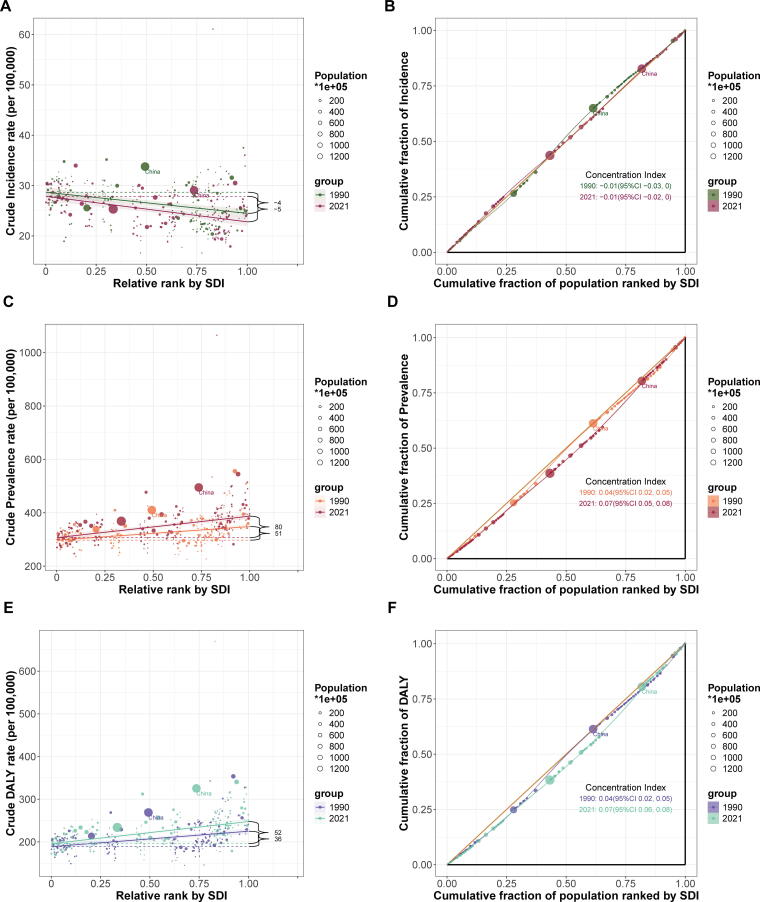
Health inequality analysis of the global burden of schizophrenia among women of reproductive age in 1990 and 2021. **(A)** Slope index of crude prevalence rate of schizophrenia among women of reproductive age in 1990 and 2021. **(B)** Concentration index of prevalence of schizophrenia among women of reproductive age in 1990 and 2021. **(C)** Slope index of crude incidence rate of schizophrenia among women of reproductive age in 1990 and 2021. **(D)** Concentration index of incidence of schizophrenia among women of reproductive age in 1990 and 2021. **(E)** Slope index of crude DALY rate due to schizophrenia among women of reproductive age in 1990 and 2021. **(F)** Concentration index of DALYs due to schizophrenia among women of reproductive age in 1990 and 2021.

The health inequality analysis of schizophrenia among WRA revealed significant disparities across different SDI levels from 1990 to 2021 (Fig. 8). For incidence, the SII showed a slight increase in absolute inequality, from −4 in 1990 to −5 in 2021 (Fig. 8A). The concentration index (CIX) for incidence remained stable at −0.01 (95% CI: −0.03 to 0 in 1990; −0.02 to 0 in 2021), indicating a consistent but minor concentration of incidence among lower SDI regions (Fig. 8B). Prevalence exhibited a more pronounced increase in inequality, with the SII rising from 51 in 1990 to 80 in 2021 (Fig. 8C). This trend was mirrored in the CIX, which increased from 0.04 (95% CI: 0.02 to 0.05) in 1990 to 0.07 (95% CI: 0.05 to 0.08) in 2021, suggesting a growing concentration of prevalence in higher SDI regions (Fig. 8D). The DALY showed a similar pattern to prevalence, with the SII increasing from 36 in 1990 to 52 in 2021 (Fig. 8E), and the CIX rising from 0.04 (95% CI: 0.02 to 0.05) to 0.07 (95% CI: 0.06 to 0.08) over the same period (Fig. 8F). These results indicate that while the incidence of schizophrenia among WRA remained slightly more concentrated in lower SDI regions, both prevalence and DALY became increasingly concentrated in higher SDI regions over the three decades, suggesting a widening gap in the burden of schizophrenia across different sociodemographic levels.

### Projections of schizophrenia burden among women of reproductive age globally and in China and India to 2040

**FIG. 9.**
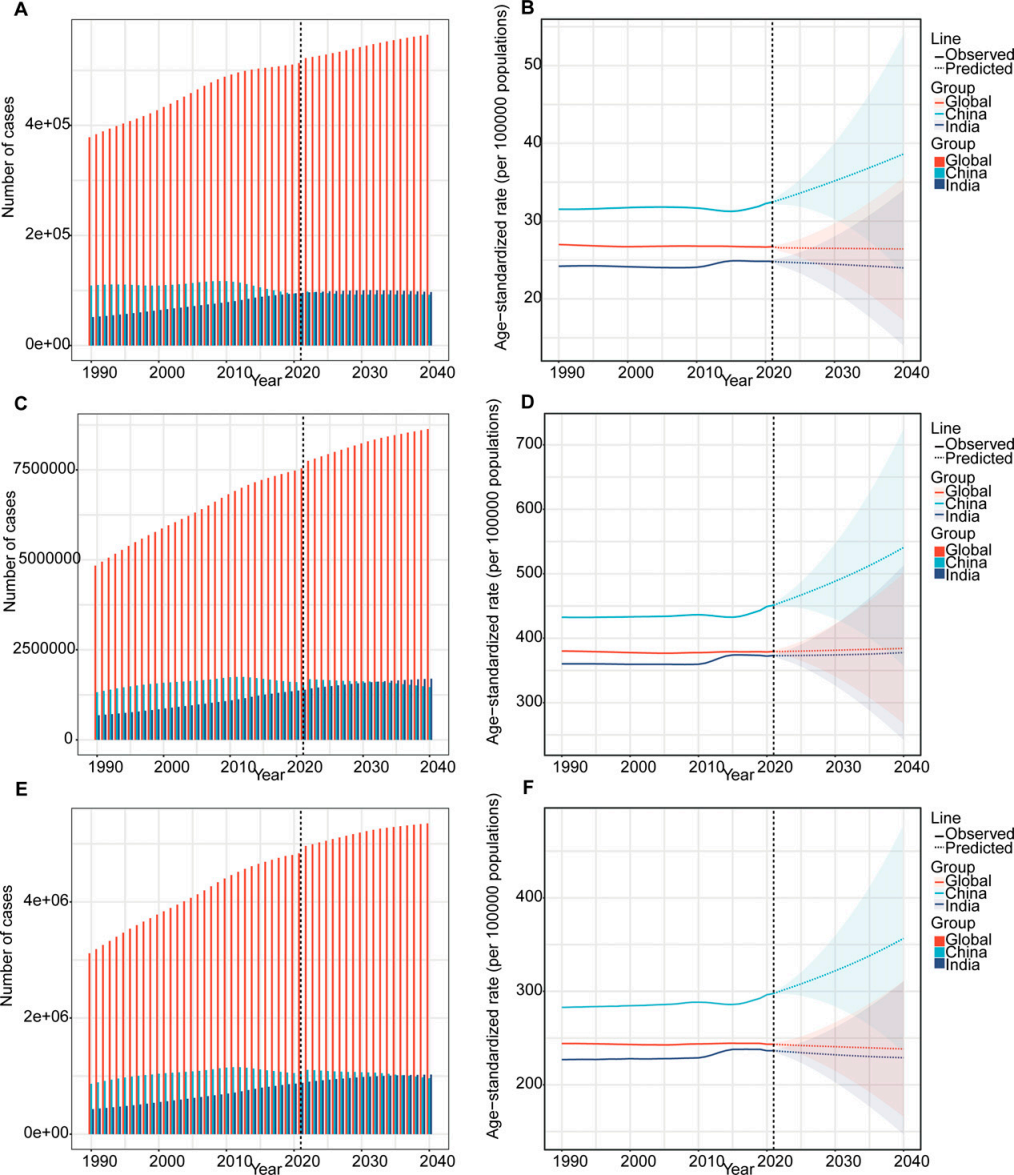
Projected burden of schizophrenia among women of reproductive age globally and in China in 2040. **(A, B)** Incidence of schizophrenia among women of reproductive age globally and in China in 2040. **(C, D)** Prevalence of schizophrenia among women of reproductive age globally and in China in 2040. **(E, F)** DALYs due to schizophrenia among women of reproductive age globally and in China in 2040.

Given that China and India are the world’s two most populous countries, each with over 1.4 billion people, their disease burden patterns significantly influence global health trends and require specific attention for public health planning. Globally, from 2022 to 2040, the incidence, prevalence, and number of cases of schizophrenia in women of childbearing age will show a gradual increase, corresponding to a gradual decrease in the ASR. The number of incidence cases is projected to increase from 0.05 million cases in 2022 to 0.56 million cases in 2040, with the ASR decreasing from 26.57 to 26.43; the number of prevalence cases will increase from 7.71 million cases in 2022 to 8.66 million cases in 2040, with the ASR decreasing from 371.23 to 370.43, and the number of DALYs cases will increase from 4.96 million in 2022 to 5.35 million in 2040, and the ASR will decrease from 243.02 to 238.46 (Supplementary Table S9 and Fig. 9).

Within China, from 2022 to 2040, the incidence rate, prevalence rate, and the number of cases of schizophrenia in women of childbearing age will show a gradual decreasing trend, corresponding to a gradual increase in ASR. The number of incidence cases is projected to decrease from 0.098 million cases in 2022 to 0.092 million cases in 2040, with ASR increasing from 32.72 to 38.63; the number of prevalence cases will decrease from 1.63 million cases in 2022 to 1.36 million cases in 2040, with ASR increasing from 420.90 to 455.01, and the number of DALYs cases will decrease from 1.11 million in 2022 to 0.96 million cases in 2040 with ASR increasing from 300.34 to 356.62 (Supplementary Table S9 and Fig. 9).

Within India, from 2022 to 2040, the number of schizophrenia incidence cases in women of childbearing age will increase gradually during 2022–2034 and then decrease gradually during 2035–2040, with a gradual decrease in its ASR; the prevalence and the number of DALYs cases will increase gradually during 2022–2040, with the ASR of prevalence gradually decreasing from 2022 to 2030 and then increasing from 2031 to 2040, while the ASR for DALYs will gradually decrease from 2022 to 2040. The number of incidence cases is projected to increase from 0.096 million in 2022–0.100 million in 2034 and then decrease to 0.097 million in 2040, with its ASR decreasing from 24.74 to 23.99; the number of prevalence cases will increase from 1.43 million in 2022 to 1.74 million in 2040, with its ASR decreasing from 352.94 in 2022 to 352.62 in 2030 and then increasing to 359.68 in 2040; the number of DALYs cases will increase from 0.91 million in 2022 to 1.03 million in 2040 with ASR decreasing from 235.89 to 229.03 (Supplementary Table S9 and Fig. 9).

## Discussion

This comprehensive 32-year global analysis reveals complex patterns in the burden of schizophrenia among women of reproductive age from 1990 to 2021. While absolute case numbers increased substantially across all measures—with incidence rising by 35.68%, prevalence by 55.82%, and DALYs by 55.27%—the underlying epidemiological trends showed more nuanced patterns. Age-standardized incidence rates demonstrated a slight but consistent global decline (EAPC: −0.022), while prevalence and DALY rates remained relatively stable with minimal changes. These findings indicate that population growth and demographic shifts, rather than increased disease risk, have been the primary drivers of the growing absolute burden. Notably, our decomposition analysis confirmed that population growth accounted for over 84% of the increase across all burden measures globally, with aging contributing an additional 15–16% to prevalence and DALY increases. The burden exhibited significant heterogeneity across sociodemographic contexts, with low SDI regions experiencing the largest increases in absolute numbers (144–150% across measures), while high SDI regions showed more modest growth (3–12%). Furthermore, a clear age gradient emerged, with older reproductive age groups (particularly 45–49 years) experiencing disproportionately larger increases in burden compared to younger cohorts, suggesting important implications for targeted intervention strategies.

Our findings reveal that the burden of schizophrenia among women of reproductive age has increased substantially globally, with significant regional and sociodemographic disparities. These results align with previous research demonstrating the complex interplay between population dynamics, health care systems, and mental health outcomes. Schizophrenia is characterized by high recurrence rates, low medication compliance due to adverse effects including metabolic disturbances and sexual dysfunction, and substantial comorbidities including depression and anxiety disorders, which collectively contribute to its persistent global burden. The relatively stable ASR observed in our study are consistent with a previous study that showed no significant increase in the ASR of prevalence of schizophrenia.^28^ This may be due to early intervention that effectively controls symptoms and reduces the recurrence rate and severity of the disease.^2^

The sociodemographic disparities observed in our study reveal important patterns in schizophrenia burden distribution. The present study found that the absolute number of cases of schizophrenia was highest among women of childbearing age in the medium SDI region in 2021. This trend may be attributed to improved health care and increased awareness of mental health issues in the mid-SDI region. In contrast, the burden of schizophrenia increased significantly in low SDI regions, reflecting the challenges posed by a lack of resources and poor health care.^29,30^ High SDI regions had lower case counts and growth rates, suggesting that their well-established health care support systems are effective in controlling the prevalence of schizophrenia.^17^ However, our age-standardized analysis shows that higher SDI regions demonstrate increasing trends in prevalence (EAPC: 0.174 in high-middle SDI regions) and DALYs (EAPC: 0.194 in high-middle SDI regions), while lower SDI regions, despite having the highest absolute increases, show more stable or declining ASR. This pattern may reflect improved diagnostic capacity and health care access in higher SDI regions, along with lifestyle changes and urbanization stress associated with economic development.^2^ These results emphasize the importance of adopting differentiated strategies in different SDI regions, especially in low and medium SDI regions, and the need to strengthen mental health resource allocation and public health policies to reduce the global gap in mental health services.^17^

Our study revealed regional disparities in the epidemiological features of schizophrenia among women of reproductive age. Over the past 32 years, the prevalence, incidence, and DALYs associated with schizophrenia have generally increased across most regions. However, certain high SDI/high-medium SDI regions, such as Eastern and Central Europe and the high-income Asia-Pacific region, have shown varying trends. The observed increases in Eastern Europe, with the highest positive EAPC in prevalence (0.201) and DALYs (0.211), may be attributed to post-transition economic and social instability, changes in health care systems, and lifestyle modifications following political transitions. The GBD 2016 report indicates a significant and growing burden of schizophrenia, largely driven by population growth and aging, particularly in middle-income countries.^6^ In Eastern Europe and Southeast Asia, despite economic growth in some areas, the risk of schizophrenia may be exacerbated by urbanization, increasing social pressures during industrialization, and lifestyle changes. In contrast, regions where the prevalence and DALYs have decreased may owe this trend to more robust health care systems and public health management. Continued monitoring of trends in these regions could help inform global prevention and control strategies for schizophrenia in women of reproductive age and guide the development of targeted prevention and treatment measures.

Country-level analysis reveals substantial heterogeneity that requires careful interpretation of age-standardized data rather than crude values. From 1990 to 2021, the most significant increase in ASR of schizophrenia globally was observed in Georgia and Denmark, which may be related to multifaceted interactions between genetic and environmental risk factors.^31^ Georgia’s dramatic increase in age-standardized incidence rates (115.61% increase, EAPC: 2.5) and Denmark’s consistent increases across all metrics (prevalence EAPC: 1.51) represent genuine epidemiological changes that warrant investigation. In addition, studies have also shown that the higher incidence of schizophrenia is related to urban living and immigration status,^2^ which may also explain the differences between countries. Our study shows significant differences in the prevalence, incidence, and cases of DALYs among women of childbearing age in different countries. The data show a polarization trend in medium or high SDI regions, with differences of up to seven times between some countries. Although countries such as Qatar and the United Arab Emirates have observed the highest percentage changes in crude schizophrenia cases among women of childbearing age, they also have high global population growth rates^32^ and there may be a link between the percentage changes in cases and population increases or decreases. However, when examining ASR, these patterns differ significantly, emphasizing the importance of using age-adjusted data for epidemiological interpretation. Second, the percentage decline observed in a small number of countries may indicate that psychiatric disorders such as schizophrenia are effectively controlled in that country and that treatment outcomes for the disease are better. Studies of the decline in these countries may help to understand strategies for the prevention and control of schizophrenia in women of reproductive age worldwide.

The age-group-specific patterns observed in our study provide crucial insights into the epidemiology of schizophrenia among women of reproductive age. In terms of age patterns, the 45–49-year-old age group is the most prominent in terms of the new burden of schizophrenia globally in 2021. This may suggest that this age group faces specific environmental stressors, physiological changes, or other as yet unspecified factor interactions that lead to a high prevalence of schizophrenia in this age group. The most substantial increases in the 45–49 age group (109.44% increase in incidence cases) align with established knowledge about the protective effects of estrogen and the vulnerability period around menopause. Research demonstrates that estradiol has protective effects against schizophrenia onset and symptom severity, with randomized controlled trials showing significant improvement in positive symptoms (effect size 0.44) when estradiol supplementation is provided.^8,9^ This result has important implications for the allocation of public health resources and the development of targeted preventive measures, suggesting that more attention and resources need to be devoted to this age group. Globally, the prevalence, incidence, and DALY of schizophrenia among women of reproductive age have increased with age over the past 32 years, with the highest increase in the 45–49 age group. This phenomenon may be because women in this age group face physiological changes in the pre-menopausal or early menopausal period, such as fluctuating hormone levels, *etc.* These physiological factors, in conjunction with psychological stress and changes in social roles, increase the risk of developing schizophrenia. It has been found that sex hormones, particularly estrogen, have a degree of protective effect on female schizophrenic patients, and that reduced estrogen in women exacerbates the risk of schizophrenia and prolongs the course of schizophrenia.^33^ Estradiol is the main female sex hormone, and studies have shown a higher prevalence of psychiatric disorders during periods of sudden decline in female estrogen levels such as menopause, after childbirth, and at the end of the luteal phase of the menstrual cycle.^34^ Additionally, menopausal hormone therapy has demonstrated protective effects against psychosis relapse in women with schizophrenia, with a 16% lower relapse risk during hormone therapy use, particularly effective in women aged 40–55 years.^20^

The percentage change in the number of females with schizophrenia in the childbearing age group showed different trends with age in the five sociodemographic index regions. The greatest percentage change across age groups was found in the low sociodemographic index areas. This reflects the fact that low sociodemographic index areas may have some generalized disadvantages, such as poverty, lack of medical resources, and low levels of education, which have a more severe impact on women of childbearing age in all age groups. However, lower SDI regions face significant challenges in schizophrenia diagnosis and treatment due to limited mental health resources, competing health priorities such as infectious diseases, social stigma surrounding mental illness, and geopolitical instabilities that disrupt health care systems.^13^ The substantial under-diagnosis in these regions may mask the true burden of schizophrenia, with many cases remaining undetected and untreated due to lack of trained mental health professionals, diagnostic facilities, and awareness about mental health conditions. This diagnostic gap is exacerbated by cultural factors where mental health symptoms may be attributed to spiritual or social causes rather than medical conditions, leading to delayed or inappropriate treatment. In contrast, the percentage change in the number of people with schizophrenia increases gradually with age in the low to medium SDI to high SDI areas. This suggests that the impact of aging-related factors on the prevalence of schizophrenia among women of childbearing age is more pronounced in areas with relatively better levels of economic and social development. With aging, women in these areas face more complex psychological stressors such as social competition and family pressure, as well as a lack of adequate coping resources during physiological changes, which leads to a gradual increase in the prevalence rate. Epidemiological investigations have shown that social pressure, employment, and marital pressure increase the risk of schizophrenia, and adverse psychological factors may have a certain impact on the function of the cerebral cortex, the immune system, and the endocrine system.^35^ In recent years, the pressures of women’s roles at work and home, as well as the accelerated pace of life, have also led to a significant increase in the proportion of female patients suffering from schizophrenia.^36^ However, as economic conditions improve (from low to high sociodemographic indices), the percentage change across age groups gradually decreases. This reflects that economic development and the abundance of social resources have a positive effect on the prevention and control of schizophrenia. Taken together, these data can help guide the development of public health strategies and resource allocation for schizophrenia worldwide.

The trends in schizophrenia burden relative to SDI are of great significance. The overall upward trend with economic progress (increasing SDI) might be due to lifestyle changes in developed regions.^37^ When SDI is 0.4–0.6 and the burden declines, it could indicate effective local policies. In regions like Australasia and high-income North America with higher SDI and increased burden, rapid economic growth may lead to factors like stress and disrupted social structures.^38^ For the Caribbean and Eastern Europe with lower burden, within their SDI range, it may be that their social systems, such as strong community and family support, mitigate the impact of schizophrenia.^39^ Understanding these differences helps in formulating region-specific interventions to manage the schizophrenia burden effectively.

Our decomposition analysis reveals that population growth is the primary driver of increased schizophrenia burden globally (85.4% of DALY increase), with aging contributing substantially (15.33% of DALY increase) and epidemiological changes having minimal impact. However, this pattern varies significantly across SDI regions, reflecting different demographic transitions and health care contexts. In high SDI regions, aging plays a more prominent role (39.41% of DALY increase), consistent with advanced demographic transition and longer life expectancy. The positive epidemiological changes observed in high-middle SDI regions (contributing 25.73% to DALY increase) may reflect improved diagnostic capacity and health care access rather than true increases in disease occurrence.

Projections based on available datasets indicate a gradual decline in the ASR of schizophrenia worldwide, despite an increase in incidence, prevalence, and DALYs. This suggests that, in the context of global demographic shifts, while the absolute number of cases has risen, the rate of growth is slowing relative to population size. However, this does not imply that the issue of schizophrenia in women of reproductive age can be overlooked, and addressing the global burden of schizophrenia in this group remains an urgent concern.^40^ The contrasting patterns between China (declining absolute burden with increasing ASR) and India (mixed patterns with regional variations) reflect different demographic transitions and health care development trajectories. Therefore, in addition to addressing known risk factors, such as genetic predisposition and environmental stress, it is crucial to place greater emphasis on the mental health of women of childbearing age, particularly in light of increasing social pressures.^41^

## Limitation

The study has several limitations. First of all, the estimates presented in this article are not comprehensive, as we only generalize “schizophrenia” in the WRA and do not rise to the level of “mental illness.” Due to the low level of basic medical care in some underdeveloped countries, misdiagnosis and incomplete diagnosis may occur, resulting in a low burden in individual regions. Second, the data obtained from GBD relies heavily on the established model data, and GBD collaborators use a variety of statistical modeling methods, resulting in results that may be inconsistent with reality in countries where raw data is lacking. Third, the burden of schizophrenia is composed of many aspects, and the description of the burden of schizophrenia from the incidence rate, prevalence rate, and DALYs ASR is still very one-sided. Therefore, the selection of the method to measure the burden data and how to ensure the accuracy of the data have not been fully considered. Finally, the most important limitation to note is the temporal lag inherent in GBD data collection and processing. The GBD methodology requires extensive data harmonization, statistical modeling, and validation processes that result in a delay between data collection and publication, meaning our 2021 estimates may not fully capture the most recent epidemiological trends or the impact of recent global events such as the COVID-19 pandemic on mental health outcomes. This temporal limitation has important implications for policy planning and intervention strategies. Therefore, on the one hand, there is a need for further development of disability weights and severity coefficients involving patient functional impairment, which may provide more standardized information for future GBD definitions of schizophrenia burden. Additionally, real-time surveillance systems and more frequent large-scale epidemiological studies are needed to validate and update these results for more accurate, timely, and comprehensive assessment of schizophrenia burden among women of reproductive age globally.

## Conclusions

This comprehensive analysis of the global burden of schizophrenia among women of reproductive age from 1990 to 2021 reveals a complex landscape of increasing absolute burden driven primarily by population growth (85.4% of DALY increase) and aging (15.33% of DALY increase), despite relatively stable ASR globally (EAPC: 0.005 for DALYs). Significant disparities were observed across sociodemographic index regions, with low SDI areas experiencing the largest absolute increases (147.92% increase in prevalence cases), while high-middle SDI regions demonstrated the highest age-standardized rate increases (EAPC: 0.174 for prevalence, 0.194 for DALYs). Age-group-specific analysis revealed that the 45–49 age group bore the greatest burden increase (109.44% increase in incidence cases), consistent with declining estrogen protection during the perimenopausal period. Health inequality analysis demonstrated widening disparities, with prevalence and DALYs becoming increasingly concentrated in higher SDI regions (concentration index increasing from 0.04 to 0.07), reflecting complex interactions between diagnostic capacity, health care access, and true disease burden. Regional variations highlighted the heterogeneous nature of schizophrenia burden, with Eastern Europe showing the highest age-standardized increases and substantial country-level differences requiring tailored national strategies. Projections to 2040 indicate continued global increases in absolute burden with declining ASR, emphasizing the need for population-focused interventions in rapidly growing low SDI regions and specialized care strategies in higher SDI areas where the concentration of long-term burden is increasing. These findings underscore the urgent need for differentiated, evidence-based approaches to schizophrenia prevention and management that account for regional demographic transitions, health care infrastructure, and socioeconomic contexts to effectively address the evolving global burden of this debilitating condition among women of reproductive age.

## Supplementary Material

Supplementary Tables

## Data Availability

The data used for the analyses in the study are publicly available at https://ghdx.healthdata.org/gbd-2021. All the data supporting the study’s findings were shown in this article and are available upon reasonable request.

## Abbreviations Used

ASR: Age-Standardized Rate
BAPC: Bayesian Age-Period-Cohort
CI: Confidence Interval
CIX: Concentration Index
DALYs: Disability-Adjusted Life Years
EAPC: Estimated Annual Percentage Change
GBD: Global Burden of Disease
ICD: International Classification of Diseases
INLA: Integrated Nested Laplace Approximation
SDI: Socio-demographic Index
SII: Slope Index of Inequality
UI: Uncertainty Interval
WRA: Women of Reproductive Age
YLD: Years Lived with Disability
