# Supplementary material for: Global Burden of Schizophrenia Among Women of Reproductive Age from 1990 to 2021: Trends, Inequalities, and Projections to 2040
Source: Womens Health Rep (New Rochelle). 2025 Sep 16;6(1):864–87. doi: 10.1177/26884844251379002 (PMC12543430; doi:10.1177/26884844251379002)
Supplement: Supplementary Tables [file 26884844251379002_supp_tables.docx]

**Supplementray Tables：**

**Table S1：Global Prevalence of Schizophrenia among women of reproductive age in 1990 and 2021, with trends from 1990 to 2021.**

| Location | Prevalence cases | | |  | Prevalence rates | | |  |  |
| --- | --- | --- | --- | --- | --- | --- | --- | --- | --- |
|  | 1990_numbers(95% UI) | 2021_numbers(95% UI) | Percentage change in case(100%) |  | 1990_per 100 000(95% UI) | 2021_per 100 000(95% UI) | Percentage change in ASRs(100%) |  | EAPC(95% CI) |
| Global | 4840114 (3707813, 6153282) | 7541989 (5775873, 9580441) | 55.82 |  | 380.1 (293.46,479.71) | 379.43 (289.8,483.21) | -0.18 |  | -0.004 (-0.014 to 0.006) |
| Low SDI | 350210 (253232, 468211) | 868238 (628975, 1166167) | 147.92 |  | 349.04 (256.93,459.59) | 350.93 (258.31,464.78) | 0.54 |  | 0.032 (0.021 to 0.042) |
| Low-middle SDI | 887821 (652986, 1160166) | 1782349 (1327308, 2319734) | 100.76 |  | 355.24 (264.71,458.82) | 361.43 (270.44,468.35) | 1.74 |  | 0.069 (0.046 to 0.091) |
| Middle SDI | 1587619 (1209438, 2028380) | 2444267 (1870530, 3096154) | 53.96 |  | 382.45 (294.96,483.5) | 379.03 (288.42,482.55) | -0.89 |  | -0.025 (-0.04 to -0.011) |
| High-middle SDI | 1034371 (818959, 1278490) | 1346547 (1086908, 1637565) | 30.18 |  | 378.15 (300.72,465.69) | 402.71 (320.99,495.13) | 6.49 |  | 0.174 (0.157 to 0.191) |
| High SDI | 976177 (760990, 1226184) | 1095395 (857081, 1370007) | 12.21 |  | 414.41 (322.15,521.94) | 415.3 (322.08,524) | 0.21 |  | 0.024 (0.003 to 0.045) |
| High-income Asia Pacific | 176649 (133047, 227605) | 164105 (124817, 209560) | -7.10 |  | 376.68 (281.92,488.13) | 383.31 (286.61,497.27) | 1.76 |  | 0.141 (0.093 to 0.188) |
| High-income North America | 402404 (320353, 491611) | 447179 (356758, 545972) | 11.13 |  | 514.04 (408.04,629.63) | 507.75 (403.09,622.54) | -1.22 |  | -0.041 (-0.06 to -0.022) |
| Western Europe | 331615 (252157, 429137) | 337204 (258815, 433421) | 1.69 |  | 335.17 (254.23,434.94) | 328.22 (248.52,427.56) | -2.07 |  | -0.065 (-0.093 to -0.037) |
| Australasia | 25431 (21114, 30360) | 35999 (30170, 42874) | 41.56 |  | 464.05 (384.52,554.49) | 463.8 (386.22,555.06) | -0.05 |  | 0.003 (-0.005 to 0.01) |
| Andean Latin America | 26117 (17973, 36522) | 53770 (37509, 74105) | 105.88 |  | 302.64 (212.15,417.12) | 305.66 (213.59,420.87) | 1.00 |  | 0.021 (0.014 to 0.027) |
| Tropical Latin America | 117788 (88851, 151359) | 201403 (153969, 255121) | 70.99 |  | 313.86 (239.13,399.82) | 316.06 (240.38,402.23) | 0.70 |  | 0.015 (0.004 to 0.025) |
| Central Latin America | 119463 (86265, 159288) | 217323 (160267, 284915) | 81.92 |  | 315.34 (231.46,414.72) | 315.34 (232.29,413.85) | 0.00 |  | -0.001 (-0.009 to 0.006) |
| Southern Latin America | 46879 (32567, 64614) | 70504 (49490, 96779) | 50.40 |  | 386.57 (269.59,531.14) | 389.03 (271.62,536.53) | 0.64 |  | 0.014 (-0.003 to 0.03) |
| Caribbean | 24387 (16975, 33721) | 33685 (23699, 46413) | 38.13 |  | 279.21 (197.23,381.63) | 275.82 (193.72,380.57) | -1.21 |  | -0.041 (-0.051 to -0.032) |
| Central Europe | 100861 (73450, 134018) | 92522 (68693, 121041) | -8.27 |  | 317.85 (230.11,424.66) | 322.33 (234.81,429.27) | 1.41 |  | 0.041 (0.033 to 0.048) |
| Eastern Europe | 168162 (127836, 214588) | 164793 (126639, 207039) | -2.00 |  | 289.69 (219.52,370.77) | 302.74 (228.92,386.41) | 4.50 |  | 0.201 (0.159 to 0.243) |
| Central Asia | 49078 (33987, 68648) | 79030 (55255, 108945) | 61.03 |  | 311.05 (219.81,428.43) | 311.39 (217.12,430.96) | 0.11 |  | 0.011 (-0.002 to 0.024) |
| North Africa and Middle East | 488739 (350165, 662637) | 1121036 (811644, 1497349) | 129.37 |  | 346.24 (252.55,461.4) | 347.37 (251.09,464.7) | 0.33 |  | -0.008 (-0.014 to -0.001) |
| South Asia | 1709891 (1284589, 2188951) | 3602885 (2726692, 4597909) | 110.71 |  | 361.81 (274.46,459.12) | 371.06 (281.72,472.12) | 2.56 |  | 0.096 (0.064 to 0.127) |
| Southeast Asia | 434154 (314840, 577818) | 738740 (550774, 964488) | 70.16 |  | 384.84 (283.51,506.41) | 394.54 (293.19,516.48) | 2.52 |  | 0.156 (0.117 to 0.195) |
| East Asia | 1362232 (1082707, 1674334) | 1626727 (1319691, 1963377) | 19.42 |  | 430.39 (345.66,525.12) | 448.51 (358.91,546.62) | 4.21 |  | 0.073 (0.045 to 0.101) |
| Oceania | 5296 (3621, 7482) | 12574 (8718, 17419) | 137.42 |  | 369.78 (258,513.53) | 371.52 (259.58,511.42) | 0.47 |  | 0 (-0.022 to 0.021) |
| Western Sub-Saharan Africa | 148854 (107679, 199856) | 417486 (303379, 558523) | 180.47 |  | 388.79 (287.43,512.41) | 390.61 (288.98,514.62) | 0.47 |  | 0.022 (0.017 to 0.027) |
| Eastern Sub-Saharan Africa | 124491 (88743, 169216) | 317413 (228454, 430101) | 154.97 |  | 330.36 (241.05,440.61) | 332.51 (243.9,443.41) | 0.65 |  | 0.042 (0.032 to 0.053) |
| Central Sub-Saharan Africa | 36351 (24915, 51333) | 95635 (66087, 134250) | 163.09 |  | 330.29 (231.58,457.77) | 325.1 (228.75,449.46) | -1.57 |  | -0.027 (-0.048 to -0.005) |
| Southern Sub-Saharan Africa | 40587 (29895, 53209) | 73936 (55624, 95907) | 82.17 |  | 339.81 (254.64,439.32) | 339.32 (255.73,439.46) | -0.14 |  | 0.021 (0.011 to 0.031) |

**Table S2：Disability-adjusted life years (DALYs) due to Schizophrenia among women of reproductive age in 1990 and 2021, with trends from 1990 to 2021.**

| Location | DALY cases | | |  | | DALY rates | | |  | |
| --- | --- | --- | --- | --- | --- | --- | --- | --- | --- | --- |
|  | 1990_numbers(95% UI) | 2021_numbers(95% UI) | Percentage change in case(100%) | |  | 1990_per 100 000(95% UI) | 2021_per 100 000(95% UI) | Percentage change in ASRs(100%) | | EAPC(95% CI) |
| Global | 3114984 (2138675, 4238277) | 4836703 (3318473, 6567286) | 55.27 | |  | 244.12 (168.33,329.91) | 243.46 (166.79,331.29) | -0.27 | | 0.005 (-0.004 to 0.013) |
| Low SDI | 221196 (145125, 312452) | 552163 (364040, 780207) | 149.63 | |  | 219.69 (146.04,306.07) | 222.41 (148.23,310.27) | 1.24 | | 0.071 (0.059 to 0.083) |
| Low-middle SDI | 564224 (380198, 782537) | 1134533 (765367, 1583760) | 101.08 | |  | 225.05 (152.9,308.25) | 229.78 (155.49,319.34) | 2.10 | | 0.094 (0.072 to 0.115) |
| Middle SDI | 1029028 (703067, 1410514) | 1576391 (1085696, 2141396) | 53.19 | |  | 247.12 (170.17,335.27) | 244.76 (168.02,333.99) | -0.96 | | -0.018 (-0.03 to -0.006) |
| High-middle SDI | 672326 (467381, 898636) | 874087 (614730, 1154235) | 30.01 | |  | 245.4 (170.92,327.05) | 262.16 (183.06,349.75) | 6.83 | | 0.194 (0.18 to 0.208) |
| High SDI | 625687 (434444, 846991) | 696195 (485441, 928011) | 11.27 | |  | 265.73 (184.12,360.7) | 264.42 (183.43,355.41) | -0.49 | | 0.015 (-0.01 to 0.04) |
| High-income Asia Pacific | 114729 (77400, 158883) | 106485 (72599, 146142) | -7.19 | |  | 244.9 (164.58,341.03) | 249.5 (168.01,347.54) | 1.88 | | 0.149 (0.102 to 0.197) |
| High-income North America | 256201 (181673, 339040) | 279996 (198177, 368294) | 9.29 | |  | 327.38 (231.52,434.21) | 318.29 (224.62,420.46) | -2.78 | | -0.074 (-0.098 to -0.051) |
| Western Europe | 211485 (144166, 292825) | 213924 (145945, 291357) | 1.15 | |  | 213.85 (145.53,297) | 208.66 (141.2,287.71) | -2.43 | | -0.067 (-0.096 to -0.037) |
| Australasia | 16151 (11008, 21890) | 22786 (15761, 30509) | 41.08 | |  | 294.74 (200.5,400.09) | 293.96 (202.28,395.85) | -0.26 | | 0.002 (-0.006 to 0.01) |
| Andean Latin America | 16860 (10165, 25596) | 34512 (20991, 51594) | 104.70 | |  | 194.82 (119.27,290.55) | 196.14 (119.39,293.12) | 0.68 | | 0.037 (0.025 to 0.048) |
| Tropical Latin America | 74646 (50293, 103442) | 126685 (86015, 172103) | 69.71 | |  | 198.43 (134.47,272.8) | 199.05 (134.82,271.53) | 0.31 | | 0.023 (0.004 to 0.041) |
| Central Latin America | 76792 (50135, 109813) | 138687 (93391, 195251) | 80.60 | |  | 202.02 (133.3,284.96) | 201.3 (135.46,283.64) | -0.36 | | -0.007 (-0.017 to 0.004) |
| Southern Latin America | 30027 (18483, 44134) | 44689 (27665, 65283) | 48.83 | |  | 247.47 (152.84,362.62) | 246.78 (152.04,362.14) | -0.28 | | 0.013 (-0.007 to 0.032) |
| Caribbean | 15675 (9835, 23367) | 21475 (13708, 31547) | 37.00 | |  | 179.06 (113.58,263.89) | 175.9 (112.05,258.71) | -1.76 | | -0.044 (-0.053 to -0.035) |
| Central Europe | 65027 (42702, 91369) | 59564 (39572, 82440) | -8.40 | |  | 205.08 (134.2,289.62) | 208.33 (136.66,292.93) | 1.58 | | 0.063 (0.054 to 0.072) |
| Eastern Europe | 107986 (73516, 147464) | 105114 (71644, 141678) | -2.66 | |  | 186.03 (126.15,255.05) | 193.92 (130.8,265.36) | 4.24 | | 0.211 (0.168 to 0.255) |
| Central Asia | 31813 (20101, 47603) | 50917 (32148, 74598) | 60.05 | |  | 200.93 (128.58,296.31) | 200.69 (126.23,295.3) | -0.12 | | 0.023 (0.008 to 0.038) |
| North Africa and Middle East | 310641 (202434, 444543) | 708272 (469267, 1011143) | 128.00 | |  | 219.24 (144.88,309.25) | 219.52 (145.26,313.92) | 0.13 | | -0.006 (-0.013 to 0.001) |
| South Asia | 1082238 (735574, 1479422) | 2287815 (1559106, 3136025) | 111.40 | |  | 228.33 (156.11,309.09) | 235.38 (160.72,321.62) | 3.09 | | 0.127 (0.098 to 0.155) |
| Southeast Asia | 282665 (185712, 401691) | 480916 (322313, 669290) | 70.14 | |  | 249.91 (165.53,351) | 257.01 (171.91,358.59) | 2.84 | | 0.176 (0.137 to 0.214) |
| East Asia | 893224 (621394, 1197732) | 1069773 (755348, 1409687) | 19.77 | |  | 281.46 (196.96,374.74) | 295.9 (207.28,393.75) | 5.13 | | 0.106 (0.081 to 0.131) |
| Oceania | 3418 (2084, 5090) | 8096 (4945, 12207) | 136.86 | |  | 238.01 (147.47,349.35) | 238.94 (146.82,358.18) | 0.39 | | 0.009 (-0.01 to 0.028) |
| Western Sub-Saharan Africa | 94388 (62049, 134465) | 266315 (174938, 375887) | 182.15 | |  | 245.42 (163.91,343.73) | 248.2 (165.12,345.52) | 1.13 | | 0.057 (0.049 to 0.065) |
| Eastern Sub-Saharan Africa | 78930 (51044, 113501) | 202311 (131136, 289608) | 156.32 | |  | 208.61 (137.18,295.17) | 211.12 (138.53,297.67) | 1.20 | | 0.086 (0.071 to 0.102) |
| Central Sub-Saharan Africa | 22743 (13574, 34385) | 60440 (36906, 90571) | 165.75 | |  | 205.9 (125.6,306.16) | 204.75 (127.02,301.9) | -0.56 | | 0.024 (-0.004 to 0.051) |
| Southern Sub-Saharan Africa | 25785 (17078, 36218) | 45975 (30907, 63777) | 78.30 | |  | 215.04 (143.91,298.09) | 210.8 (141.82,291.98) | -1.97 | | -0.035 (-0.055 to -0.016) |

**Table S3：** Country-specific Incidence of Schizophrenia among women of reproductive age in 1990 and 2021, with trends from 1990 to 2021 across 204 countries.

| Location | Incidence cases | | |  | | Incidence rates | | |  | |
| --- | --- | --- | --- | --- | --- | --- | --- | --- | --- | --- |
|  | 1990_numbers(95% UI) | 2021_numbers(95% UI) | Percentage change in case(100%) |  | 1990_per 100 000(95% UI) | | 2021_per 100 000(95% UI) | Percentage change in ASRs(100%) | | EAPC(95% CI) |
| Afghanistan | 531 (287, 846) | 1816 (962, 2967) | 242.00 |  | 23.77 (12.84,37.56) | | 23.46 (12.45,38.23) | -1.30 | -0.06 (-0.07 to -0.04) | |
| Albania | 203 (109, 328) | 139 (74, 224) | -31.53 |  | 22.61 (12.19,36.34) | | 22.88 (12.16,36.96) | 1.19 | 0.01 (0.01 to 0.02) | |
| Algeria | 1621 (855, 2624) | 2721 (1431, 4437) | 67.86 |  | 25.25 (13.37,40.75) | | 24.98 (13.1,40.85) | -1.07 | -0.05 (-0.05 to -0.04) | |
| American Samoa | 4 (2, 6) | 3 (2, 5) | -25.00 |  | 29.87 (15.79,48.59) | | 29.53 (15.53,47.46) | -1.14 | -0.02 (-0.03 to 0) | |
| Andorra | 4 (2, 6) | 5 (3, 8) | 25.00 |  | 25.27 (13.58,40.92) | | 25.22 (13.32,40.95) | -0.20 | -0.01 (-0.02 to -0.01) | |
| Angola | 638 (342, 1034) | 2088 (1121, 3415) | 227.27 |  | 26.29 (14.2,42.38) | | 26.15 (14.16,42.53) | -0.53 | 0 (-0.01 to 0.02) | |
| Antigua and Barbuda | 3 (2, 6) | 5 (2, 8) | 66.67 |  | 20.22 (10.25,33.23) | | 20.07 (10.41,32.74) | -0.74 | 0 (-0.01 to 0.01) | |
| Argentina | 2174 (1155, 3514) | 3204 (1692, 5135) | 47.38 |  | 27.11 (14.42,43.76) | | 27.3 (14.37,43.82) | 0.70 | -0.01 (-0.03 to 0.01) | |
| Armenia | 214 (116, 341) | 161 (86, 257) | -24.77 |  | 23.09 (12.5,36.85) | | 23.12 (12.27,37.1) | 0.13 | -0.01 (-0.02 to 0) | |
| Australia | 1395 (898, 2000) | 1819 (1167, 2616) | 30.39 |  | 31.62 (20.45,45.16) | | 31.63 (20.5,45.01) | 0.03 | 0.02 (0.01 to 0.02) | |
| Austria | 495 (265, 811) | 478 (259, 769) | -3.43 |  | 24.86 (13.32,40.77) | | 24.89 (13.37,40.43) | 0.12 | 0 (0 to 0.01) | |
| Azerbaijan | 479 (255, 762) | 622 (333, 999) | 29.85 |  | 22.93 (12.28,36.59) | | 23 (12.32,37.07) | 0.31 | 0.02 (0 to 0.03) | |
| Bahamas | 15 (8, 26) | 21 (11, 34) | 40.00 |  | 20.03 (10.3,33.19) | | 19.87 (10.25,32.47) | -0.80 | -0.01 (-0.02 to -0.01) | |
| Bahrain | 33 (17, 54) | 83 (43, 133) | 151.52 |  | 25.87 (13.46,41.75) | | 25.83 (13.39,41.48) | -0.15 | -0.02 (-0.02 to -0.01) | |
| Bangladesh | 6567 (3469, 10715) | 11132 (5876, 18162) | 69.51 |  | 24.16 (12.76,39.5) | | 23.6 (12.46,38.53) | -2.32 | -0.09 (-0.1 to -0.08) | |
| Barbados | 16 (10, 24) | 15 (8, 24) | -6.25 |  | 23.3 (14.88,33.73) | | 21.99 (11.83,35.02) | -5.62 | -0.05 (-0.09 to -0.02) | |
| Belarus | 552 (291, 890) | 416 (221, 667) | -24.64 |  | 21.63 (11.39,34.99) | | 21.75 (11.54,34.97) | 0.55 | 0 (-0.01 to 0.01) | |
| Belgium | 606 (320, 984) | 595 (320, 958) | -1.82 |  | 24.82 (13.07,40.47) | | 24.68 (13.13,39.95) | -0.56 | -0.01 (-0.02 to 0) | |
| Belize | 9 (5, 15) | 24 (13, 39) | 166.67 |  | 19.48 (10.21,31.58) | | 19.46 (10.31,31.37) | -0.10 | -0.01 (-0.01 to 0) | |
| Benin | 343 (185, 545) | 1000 (534, 1632) | 191.55 |  | 29.26 (15.94,46.44) | | 29.27 (15.75,47.48) | 0.03 | -0.02 (-0.03 to -0.01) | |
| Bermuda | 3 (2, 6) | 3 (1, 4) | 0.00 |  | 20.64 (10.83,33.8) | | 20.65 (10.78,33.96) | 0.05 | 0.03 (0.01 to 0.04) | |
| Bhutan | 38 (20, 61) | 51 (27, 82) | 34.21 |  | 24.11 (12.74,38.48) | | 23.87 (12.67,38.72) | -1.00 | -0.05 (-0.05 to -0.04) | |
| Bolivia (Plurinational State of) | 338 (177, 544) | 673 (359, 1097) | 99.11 |  | 21.07 (11.06,33.86) | | 21.11 (11.27,34.42) | 0.19 | -0.01 (-0.02 to 0) | |
| Bosnia and Herzegovina | 267 (140, 433) | 154 (82, 246) | -42.32 |  | 22.54 (11.84,36.58) | | 22.83 (12.05,36.56) | 1.29 | 0.04 (0.03 to 0.05) | |
| Botswana | 89 (47, 143) | 180 (97, 290) | 102.25 |  | 25.85 (13.94,41.68) | | 26.19 (14.07,42.26) | 1.32 | 0.06 (0.05 to 0.07) | |
| Brazil | 9162 (5391, 13529) | 12779 (7516, 18943) | 39.48 |  | 22.34 (13.13,33.07) | | 22.41 (13.17,33.2) | 0.31 | 0 (-0.01 to 0.01) | |
| Brunei Darussalam | 20 (10, 33) | 34 (18, 55) | 70.00 |  | 27.79 (14.65,45.05) | | 27.36 (14.3,44.03) | -1.55 | -0.04 (-0.05 to -0.04) | |
| Bulgaria | 432 (233, 695) | 276 (149, 438) | -36.11 |  | 21.99 (11.79,35.35) | | 21.96 (11.78,34.85) | -0.14 | 0 (-0.01 to 0) | |
| Burkina Faso | 631 (340, 1018) | 1659 (890, 2688) | 162.92 |  | 29.03 (15.76,46.49) | | 28.82 (15.62,46.42) | -0.72 | -0.03 (-0.04 to -0.02) | |
| Burundi | 352 (189, 571) | 853 (457, 1377) | 142.33 |  | 26.35 (14.3,42.64) | | 26.1 (14.09,41.85) | -0.95 | -0.02 (-0.03 to -0.01) | |
| Cabo Verde | 26 (14, 41) | 47 (25, 74) | 80.77 |  | 30.01 (16.22,47.24) | | 30.14 (16.37,47.92) | 0.43 | 0.01 (0 to 0.02) | |
| Cambodia | 725 (387, 1151) | 1269 (678, 2053) | 75.03 |  | 27.14 (14.55,43.25) | | 27.57 (14.76,44.59) | 1.58 | 0.11 (0.07 to 0.15) | |
| Cameroon | 738 (391, 1185) | 2370 (1275, 3800) | 221.14 |  | 29.22 (15.61,46.63) | | 28.91 (15.68,46.23) | -1.06 | -0.03 (-0.04 to -0.03) | |
| Canada | 1551 (1296, 1844) | 1739 (1465, 2034) | 12.12 |  | 22.18 (18.82,26.03) | | 23.05 (19.79,26.57) | 3.92 | 0.15 (0.12 to 0.19) | |
| Central African Republic | 173 (93, 280) | 361 (195, 573) | 108.67 |  | 25.68 (13.92,41.37) | | 25.37 (13.75,40.12) | -1.21 | -0.04 (-0.05 to -0.03) | |
| Chad | 403 (219, 648) | 1174 (626, 1906) | 191.32 |  | 29.13 (15.91,46.45) | | 28.97 (15.6,46.61) | -0.55 | -0.01 (-0.02 to 0) | |
| Chile | 1028 (555, 1650) | 1283 (684, 2085) | 24.81 |  | 27.4 (14.83,43.91) | | 27.63 (14.7,45.02) | 0.84 | 0.02 (0.01 to 0.04) | |
| China | 108821 (69356, 153473) | 92615 (56979, 133175) | -14.89 |  | 31.52 (19.87,44.83) | | 32.45 (20.24,46.2) | 2.95 | 0.02 (-0.01 to 0.05) | |
| Colombia | 2030 (1062, 3327) | 2875 (1505, 4661) | 41.63 |  | 21.82 (11.46,35.72) | | 21.8 (11.39,35.41) | -0.09 | -0.01 (-0.02 to 0) | |
| Comoros | 30 (16, 48) | 54 (29, 87) | 80.00 |  | 26.87 (14.43,42.65) | | 26.79 (14.36,43.3) | -0.30 | 0 (0 to 0.01) | |
| Congo | 156 (83, 249) | 378 (204, 602) | 142.31 |  | 26.14 (13.98,41.55) | | 26 (14.06,41.42) | -0.54 | 0 (-0.01 to 0.01) | |
| Cook Islands | 1 (1, 2) | 1 (1, 2) | 0.00 |  | 30 (15.88,47.88) | | 30.54 (15.81,50.18) | 1.80 | 0.06 (0.04 to 0.08) | |
| Costa Rica | 183 (96, 298) | 283 (148, 464) | 54.64 |  | 21.94 (11.62,35.82) | | 22.03 (11.56,36.26) | 0.41 | 0.01 (0 to 0.01) | |
| Coted'Ivoire | 858 (463, 1369) | 2018 (1075, 3267) | 135.20 |  | 29.1 (15.86,46.18) | | 29.03 (15.57,46.78) | -0.24 | -0.02 (-0.03 to -0.01) | |
| Croatia | 270 (143, 437) | 193 (102, 312) | -28.52 |  | 23.06 (12.16,37.46) | | 23.22 (12.17,37.77) | 0.69 | 0.01 (0 to 0.01) | |
| Cuba | 637 (333, 1044) | 468 (242, 769) | -26.53 |  | 19.94 (10.44,32.66) | | 19.87 (10.26,32.68) | -0.35 | -0.01 (-0.03 to 0.01) | |
| Cyprus | 49 (26, 79) | 85 (47, 136) | 73.47 |  | 24.79 (13.26,39.95) | | 24.74 (13.35,39.93) | -0.20 | -0.01 (-0.02 to 0) | |
| Czechia | 565 (299, 919) | 471 (255, 759) | -16.64 |  | 23.55 (12.41,38.31) | | 23.68 (12.73,38.15) | 0.55 | -0.01 (-0.02 to 0) | |
| Democratic People's Republic of Korea | 1648 (875, 2659) | 1761 (941, 2815) | 6.86 |  | 27.95 (14.84,45.05) | | 27.47 (14.66,43.95) | -1.72 | -0.08 (-0.1 to -0.06) | |
| Democratic Republic of the Congo | 2342 (1257, 3714) | 5686 (3100, 9034) | 142.78 |  | 26.06 (14.09,41.13) | | 25.67 (14.11,40.58) | -1.50 | -0.04 (-0.05 to -0.03) | |
| Denmark | 329 (222, 449) | 428 (391, 462) | 30.09 |  | 25.32 (16.97,34.78) | | 34.54 (31.51,37.31) | 36.41 | 1.44 (1.17 to 1.71) | |
| Djibouti | 28 (15, 44) | 86 (46, 137) | 207.14 |  | 26.73 (14.43,42.29) | | 26.6 (14.26,42.32) | -0.49 | 0.01 (0 to 0.02) | |
| Dominica | 4 (2, 6) | 3 (2, 5) | -25.00 |  | 19.72 (10.28,32.28) | | 19.65 (10.21,31.71) | -0.35 | 0 (-0.01 to 0.01) | |
| Dominican Republic | 399 (208, 656) | 581 (300, 951) | 45.61 |  | 19.6 (10.24,32.07) | | 19.73 (10.2,32.31) | 0.66 | 0.03 (0.02 to 0.03) | |
| Ecuador | 578 (304, 935) | 1027 (545, 1666) | 77.68 |  | 21.56 (11.37,34.94) | | 21.42 (11.37,34.79) | -0.65 | -0.01 (-0.01 to 0) | |
| Egypt | 3429 (1813, 5505) | 6630 (3586, 10548) | 93.35 |  | 24.85 (13.18,39.93) | | 24.9 (13.47,39.65) | 0.20 | -0.03 (-0.04 to -0.02) | |
| El Salvador | 299 (159, 487) | 388 (203, 624) | 29.77 |  | 21.4 (11.39,34.72) | | 21.38 (11.18,34.43) | -0.09 | 0 (-0.01 to 0.01) | |
| Equatorial Guinea | 27 (14, 43) | 102 (53, 164) | 277.78 |  | 26 (13.93,41.95) | | 26.66 (14.02,42.67) | 2.54 | 0.14 (0.11 to 0.16) | |
| Eritrea | 218 (117, 349) | 454 (244, 731) | 108.26 |  | 26.53 (14.35,42.24) | | 26.46 (14.25,42.43) | -0.26 | 0.01 (0 to 0.02) | |
| Estonia | 81 (43, 130) | 56 (29, 91) | -30.86 |  | 21.85 (11.56,35.42) | | 22.06 (11.53,35.93) | 0.96 | 0.03 (0.02 to 0.03) | |
| Eswatini | 53 (28, 87) | 85 (46, 138) | 60.38 |  | 25.82 (13.71,41.86) | | 25.73 (13.94,41.59) | -0.35 | 0.01 (0 to 0.03) | |
| Ethiopia | 3286 (1984, 4815) | 7946 (4797, 11581) | 141.81 |  | 27.57 (16.66,40.52) | | 27.19 (16.45,39.68) | -1.38 | -0.02 (-0.04 to -0.01) | |
| Fiji | 59 (31, 95) | 66 (35, 106) | 11.86 |  | 28.81 (15.29,46.37) | | 29.11 (15.41,46.7) | 1.04 | 0.04 (0.03 to 0.06) | |
| Finland | 308 (209, 428) | 249 (142, 392) | -19.16 |  | 24.93 (16.77,34.62) | | 22.42 (12.64,35.39) | -10.07 | -0.35 (-0.44 to -0.27) | |
| France | 3554 (1908, 5782) | 3357 (1805, 5374) | -5.54 |  | 24.56 (13.13,40.09) | | 24.43 (13.01,39.27) | -0.53 | -0.01 (-0.02 to 0) | |
| Gabon | 64 (34, 104) | 136 (73, 219) | 112.50 |  | 26.73 (14.4,43.22) | | 26.6 (14.32,42.77) | -0.49 | -0.02 (-0.03 to -0.01) | |
| Gambia | 72 (38, 116) | 188 (101, 299) | 161.11 |  | 29.33 (15.78,46.87) | | 28.97 (15.72,46.03) | -1.23 | -0.03 (-0.04 to -0.02) | |
| Georgia | 911 (519, 1398) | 961 (555, 1460) | 5.49 |  | 32.47 (18.54,49.89) | | 70.01 (40.87,105.3) | 115.61 | 2.5 (2.42 to 2.57) | |
| Germany | 4841 (2618, 7819) | 4087 (2229, 6570) | -15.58 |  | 24.66 (13.3,39.98) | | 24.6 (13.27,39.77) | -0.24 | -0.01 (-0.04 to 0.02) | |
| Ghana | 1093 (574, 1746) | 2776 (1466, 4432) | 153.98 |  | 29.26 (15.54,46.58) | | 29.21 (15.48,46.58) | -0.17 | 0.01 (0 to 0.01) | |
| Greece | 623 (336, 1015) | 503 (274, 803) | -19.26 |  | 24.88 (13.37,40.61) | | 24.7 (13.19,39.86) | -0.72 | -0.01 (-0.02 to 0) | |
| Greenland | 4 (2, 7) | 4 (2, 6) | 0.00 |  | 27.29 (14.56,43.75) | | 27.85 (15.07,44.19) | 2.05 | 0.07 (0.05 to 0.09) | |
| Grenada | 4 (2, 7) | 5 (3, 8) | 25.00 |  | 19.51 (10.26,31.93) | | 19.71 (10.28,32.02) | 1.03 | 0.02 (0.02 to 0.03) | |
| Guam | 11 (6, 19) | 11 (6, 18) | 0.00 |  | 30.89 (16.14,50.71) | | 30.87 (16.09,49.99) | -0.06 | 0.02 (0 to 0.04) | |
| Guatemala | 410 (216, 669) | 968 (515, 1555) | 136.10 |  | 21.09 (11.18,34.35) | | 21.08 (11.24,33.87) | -0.05 | -0.02 (-0.03 to -0.01) | |
| Guinea | 413 (222, 665) | 1006 (543, 1604) | 143.58 |  | 29.17 (15.79,46.79) | | 28.92 (15.76,45.83) | -0.86 | -0.03 (-0.04 to -0.02) | |
| Guinea-Bissau | 71 (38, 114) | 158 (84, 253) | 122.54 |  | 28.9 (15.74,46.42) | | 28.82 (15.51,45.91) | -0.28 | -0.02 (-0.03 to -0.01) | |
| Guyana | 42 (22, 69) | 40 (21, 66) | -4.76 |  | 19.23 (10,31.4) | | 19.33 (10.09,31.7) | 0.52 | 0.01 (0 to 0.02) | |
| Haiti | 303 (160, 489) | 670 (357, 1092) | 121.12 |  | 18.77 (9.93,30.28) | | 18.62 (9.94,30.33) | -0.80 | -0.04 (-0.04 to -0.03) | |
| Honduras | 236 (126, 380) | 623 (329, 1002) | 163.98 |  | 21.09 (11.24,33.82) | | 20.98 (11.13,33.69) | -0.52 | -0.04 (-0.05 to -0.03) | |
| Hungary | 539 (288, 869) | 436 (235, 698) | -19.11 |  | 22.76 (12.08,36.65) | | 22.98 (12.28,36.94) | 0.97 | 0.01 (0 to 0.02) | |
| Iceland | 16 (9, 26) | 20 (10, 32) | 25.00 |  | 24.95 (13.47,40.04) | | 24.97 (13.14,40.75) | 0.08 | 0 (0 to 0.01) | |
| India | 51612 (30429, 76966) | 95794 (56189, 143395) | 85.60 |  | 24.18 (14.18,36.27) | | 24.82 (14.53,37.24) | 2.65 | 0.09 (0.06 to 0.13) | |
| Indonesia | 14363 (8578, 21255) | 20808 (12369, 30924) | 44.87 |  | 28 (16.68,41.58) | | 28.22 (16.77,41.92) | 0.79 | 0.1 (0.06 to 0.14) | |
| Iran (Islamic Republic of) | 3510 (2110, 5147) | 5491 (3255, 8099) | 56.44 |  | 25.45 (15.25,37.38) | | 25.46 (15.22,37.45) | 0.04 | -0.02 (-0.03 to -0.01) | |
| Iraq | 1100 (581, 1753) | 2710 (1405, 4334) | 146.36 |  | 24.94 (13.26,39.59) | | 24.81 (12.91,39.65) | -0.52 | -0.02 (-0.03 to -0.01) | |
| Ireland | 251 (137, 391) | 311 (155, 515) | 23.90 |  | 28.24 (15.45,44.12) | | 29 (14.18,48.12) | 2.69 | 0.21 (0.14 to 0.27) | |
| Israel | 290 (156, 469) | 516 (280, 844) | 77.93 |  | 23.68 (12.74,38.3) | | 23.54 (12.69,38.56) | -0.59 | -0.01 (-0.02 to 0) | |
| Italy | 3112 (1878, 4601) | 2504 (1533, 3699) | -19.54 |  | 21.86 (13.17,32.33) | | 21.92 (13.32,32.46) | 0.27 | 0 (0 to 0.01) | |
| Jamaica | 127 (66, 206) | 154 (80, 247) | 21.26 |  | 19.68 (10.32,32.05) | | 19.65 (10.19,31.53) | -0.15 | -0.02 (-0.02 to -0.01) | |
| Japan | 8159 (4897, 12069) | 6373 (3784, 9394) | -21.89 |  | 26.92 (16.12,39.78) | | 28.1 (16.66,41.27) | 4.38 | 0.33 (0.22 to 0.44) | |
| Jordan | 234 (123, 376) | 801 (428, 1307) | 242.31 |  | 25.25 (13.4,40.41) | | 25.13 (13.39,40.99) | -0.48 | -0.04 (-0.05 to -0.03) | |
| Kazakhstan | 982 (521, 1566) | 1048 (556, 1700) | 6.72 |  | 22.79 (12.11,36.4) | | 22.93 (12.2,37.19) | 0.61 | 0.03 (0.03 to 0.04) | |
| Kenya | 1495 (900, 2176) | 3725 (2246, 5430) | 149.16 |  | 26.93 (16.27,39.22) | | 26.74 (16.14,39.03) | -0.71 | -0.01 (-0.02 to 0) | |
| Kiribati | 6 (3, 9) | 9 (5, 15) | 50.00 |  | 27.83 (14.71,44.63) | | 27.77 (15,44.28) | -0.22 | 0 (-0.03 to 0.02) | |
| Kuwait | 120 (62, 194) | 348 (180, 554) | 190.00 |  | 26.28 (13.68,42.56) | | 26.24 (13.61,42.15) | -0.15 | -0.02 (-0.03 to -0.01) | |
| Kyrgyzstan | 257 (137, 415) | 396 (213, 629) | 54.09 |  | 22.64 (12.13,36.52) | | 22.43 (12.09,35.69) | -0.93 | -0.03 (-0.04 to -0.02) | |
| Lao People's Democratic Republic | 279 (147, 450) | 567 (303, 924) | 103.23 |  | 27.12 (14.34,43.62) | | 27.63 (14.8,45.04) | 1.88 | 0.14 (0.11 to 0.18) | |
| Latvia | 137 (72, 221) | 77 (40, 123) | -43.80 |  | 21.76 (11.44,35.08) | | 21.82 (11.47,34.99) | 0.28 | 0.02 (0.01 to 0.03) | |
| Lebanon | 200 (106, 323) | 363 (192, 588) | 81.50 |  | 25.48 (13.43,41.29) | | 25.39 (13.44,41.23) | -0.35 | -0.02 (-0.03 to -0.02) | |
| Lesotho | 97 (52, 155) | 134 (71, 219) | 38.14 |  | 25.26 (13.61,40.15) | | 25.31 (13.55,41.1) | 0.20 | 0.03 (0.02 to 0.03) | |
| Liberia | 173 (91, 280) | 411 (222, 655) | 137.57 |  | 29.18 (15.57,47.08) | | 28.82 (15.55,45.79) | -1.23 | -0.03 (-0.03 to -0.02) | |
| Libya | 257 (136, 415) | 481 (253, 782) | 87.16 |  | 25.6 (13.59,41.15) | | 25.1 (13.13,40.84) | -1.95 | -0.1 (-0.11 to -0.08) | |
| Lithuania | 199 (107, 322) | 118 (63, 192) | -40.70 |  | 21.72 (11.65,35.19) | | 21.98 (11.65,35.77) | 1.20 | 0.02 (0.01 to 0.03) | |
| Luxembourg | 24 (13, 39) | 38 (21, 61) | 58.33 |  | 25.01 (13.15,40.44) | | 25.05 (13.36,40.33) | 0.16 | 0.03 (0.02 to 0.04) | |
| Madagascar | 757 (404, 1221) | 1995 (1059, 3263) | 163.54 |  | 26.6 (14.35,42.62) | | 26.4 (14.14,42.86) | -0.75 | -0.03 (-0.04 to -0.02) | |
| Malawi | 626 (340, 995) | 1354 (725, 2172) | 116.29 |  | 26.2 (14.37,41.47) | | 25.95 (14.04,41.38) | -0.95 | -0.02 (-0.03 to -0.01) | |
| Malaysia | 1381 (713, 2225) | 2528 (1330, 4093) | 83.06 |  | 29.01 (15.03,46.75) | | 29.51 (15.53,47.81) | 1.72 | 0.12 (0.08 to 0.16) | |
| Maldives | 15 (8, 24) | 32 (17, 52) | 113.33 |  | 28.42 (15.1,45.92) | | 28.99 (14.95,46.78) | 2.01 | 0.14 (0.1 to 0.17) | |
| Mali | 582 (316, 936) | 1664 (897, 2689) | 185.91 |  | 29.07 (15.91,46.62) | | 29 (15.76,46.62) | -0.24 | -0.02 (-0.03 to -0.01) | |
| Malta | 23 (12, 37) | 23 (12, 36) | 0.00 |  | 24.76 (13.19,39.74) | | 24.91 (13.29,40.2) | 0.61 | 0.02 (0.01 to 0.02) | |
| Marshall Islands | 3 (2, 5) | 4 (2, 7) | 33.33 |  | 27.95 (14.69,45.23) | | 28 (15.12,44.44) | 0.18 | 0.02 (0 to 0.03) | |
| Mauritania | 146 (78, 234) | 330 (175, 540) | 126.03 |  | 29.46 (15.79,46.99) | | 29.47 (15.75,47.88) | 0.03 | -0.01 (-0.02 to -0.01) | |
| Mauritius | 90 (47, 146) | 89 (47, 141) | -1.11 |  | 28.83 (14.93,46.77) | | 29.14 (15.49,46.37) | 1.08 | 0.11 (0.07 to 0.15) | |
| Mexico | 5332 (3142, 7869) | 7883 (4641, 11705) | 47.84 |  | 22.73 (13.35,33.67) | | 22.75 (13.39,33.78) | 0.09 | 0 (-0.01 to 0) | |
| Micronesia (Federated States of) | 7 (4, 11) | 8 (4, 12) | 14.29 |  | 27.99 (14.76,45.11) | | 28.13 (15.15,45.2) | 0.50 | 0.01 (-0.01 to 0.03) | |
| Monaco | 2 (1, 3) | 2 (1, 3) | 0.00 |  | 25.78 (13.6,41.63) | | 25.95 (13.47,42.16) | 0.66 | 0 (-0.01 to 0.01) | |
| Mongolia | 125 (67, 200) | 185 (101, 296) | 48.00 |  | 22.26 (12.01,35.43) | | 22.49 (12.25,35.89) | 1.03 | 0.03 (0.02 to 0.04) | |
| Montenegro | 36 (19, 58) | 31 (16, 50) | -13.89 |  | 22.78 (11.98,36.98) | | 22.81 (12.06,36.93) | 0.13 | 0 (-0.01 to 0.01) | |
| Morocco | 1677 (870, 2724) | 2359 (1245, 3815) | 40.67 |  | 24.8 (12.92,40.18) | | 24.63 (12.99,39.85) | -0.69 | -0.04 (-0.05 to -0.03) | |
| Mozambique | 838 (453, 1338) | 2064 (1112, 3307) | 146.30 |  | 26.03 (14.14,41.29) | | 25.93 (14.11,41.35) | -0.38 | 0.02 (0.01 to 0.03) | |
| Myanmar | 3028 (1628, 4886) | 4184 (2219, 6635) | 38.18 |  | 27.11 (14.65,43.63) | | 27.57 (14.61,43.75) | 1.70 | 0.15 (0.11 to 0.19) | |
| Namibia | 94 (50, 152) | 180 (96, 289) | 91.49 |  | 26.1 (13.91,41.88) | | 26.22 (14.02,42.1) | 0.46 | 0.03 (0.02 to 0.04) | |
| Nauru | 1 (0, 1) | 1 (0, 1) | 0.00 |  | 29.43 (15.44,47.31) | | 28.86 (15.16,46.24) | -1.94 | -0.04 (-0.09 to 0.01) | |
| Nepal | 1167 (611, 1861) | 2229 (1182, 3524) | 91.00 |  | 23.86 (12.48,38.15) | | 23.43 (12.42,37.16) | -1.80 | -0.09 (-0.1 to -0.08) | |
| Netherlands | 1486 (966, 2082) | 1323 (839, 1890) | -10.97 |  | 37.34 (24.19,52.45) | | 36.61 (22.99,52.58) | -1.96 | -0.08 (-0.1 to -0.06) | |
| New Zealand | 297 (173, 444) | 386 (224, 578) | 29.97 |  | 32.8 (19.21,49.02) | | 33.17 (19.34,49.53) | 1.13 | 0 (-0.02 to 0.02) | |
| Nicaragua | 210 (113, 339) | 398 (206, 642) | 89.52 |  | 21.52 (11.58,34.57) | | 21.47 (11.16,34.71) | -0.23 | -0.02 (-0.03 to -0.01) | |
| Niger | 531 (285, 860) | 1614 (863, 2574) | 203.95 |  | 29.15 (15.84,46.78) | | 28.9 (15.58,45.71) | -0.86 | -0.04 (-0.05 to -0.03) | |
| Nigeria | 7044 (4273, 10194) | 19506 (11757, 28368) | 176.92 |  | 32.57 (19.86,47.1) | | 32.38 (19.57,47.12) | -0.58 | -0.02 (-0.02 to -0.01) | |
| Niue | 0 (0, 0) | 0 (0, 0) |  |  | 29.17 (15.6,46.75) | | 29.5 (15.53,47.39) | 1.13 | 0.05 (0.04 to 0.07) | |
| North Macedonia | 116 (62, 189) | 113 (61, 180) | -2.59 |  | 22.78 (12.07,37.06) | | 22.74 (12.15,36.3) | -0.18 | -0.01 (-0.02 to 0) | |
| Northern Mariana Islands | 5 (2, 8) | 3 (2, 5) | -40.00 |  | 31.05 (16.04,50.55) | | 30.51 (15.85,48.98) | -1.74 | -0.08 (-0.11 to -0.05) | |
| Norway | 276 (165, 408) | 311 (187, 463) | 12.68 |  | 26.3 (15.7,38.88) | | 26.29 (15.76,39.09) | -0.04 | -0.01 (-0.01 to 0) | |
| Oman | 93 (48, 150) | 256 (134, 412) | 175.27 |  | 25.53 (13.42,41.23) | | 25.52 (13.44,41.23) | -0.04 | -0.02 (-0.03 to -0.01) | |
| Pakistan | 6413 (3753, 9662) | 15985 (9354, 23939) | 149.26 |  | 25.57 (14.89,38.62) | | 25.14 (14.67,37.81) | -1.68 | -0.08 (-0.09 to -0.08) | |
| Palau | 1 (1, 2) | 1 (1, 2) | 0.00 |  | 29.21 (15.65,46.96) | | 29.49 (15.52,47.43) | 0.96 | 0.03 (0.02 to 0.04) | |
| Palestine | 123 (65, 197) | 349 (179, 558) | 183.74 |  | 25.22 (13.41,40.42) | | 25.05 (12.91,40.03) | -0.67 | -0.06 (-0.08 to -0.04) | |
| Panama | 141 (75, 231) | 237 (123, 384) | 68.09 |  | 21.76 (11.55,35.54) | | 22.02 (11.46,35.79) | 1.19 | 0.03 (0.02 to 0.03) | |
| Papua New Guinea | 289 (152, 468) | 768 (405, 1211) | 165.74 |  | 28.02 (14.85,45.24) | | 28.36 (15,44.72) | 1.21 | 0.03 (0 to 0.05) | |
| Paraguay | 215 (114, 351) | 422 (222, 687) | 96.28 |  | 21.6 (11.46,35.3) | | 21.72 (11.45,35.37) | 0.56 | -0.01 (-0.02 to 0.01) | |
| Peru | 1241 (651, 1990) | 2091 (1094, 3389) | 68.49 |  | 21.48 (11.31,34.41) | | 21.61 (11.29,35.07) | 0.61 | 0.01 (0 to 0.01) | |
| Philippines | 4750 (2848, 7007) | 8643 (5170, 12756) | 81.96 |  | 28.39 (16.97,42.07) | | 28.54 (17.04,42.19) | 0.53 | 0.07 (0.04 to 0.11) | |
| Poland | 2097 (1239, 3093) | 1861 (1108, 2744) | -11.25 |  | 23.16 (13.75,34.07) | | 23.39 (13.93,34.36) | 0.99 | 0.03 (0.02 to 0.04) | |
| Portugal | 615 (328, 977) | 540 (292, 871) | -12.20 |  | 24.47 (13.05,38.92) | | 24.64 (13.06,40.14) | 0.69 | 0 (-0.01 to 0.01) | |
| Puerto Rico | 194 (101, 323) | 145 (76, 234) | -25.26 |  | 20.31 (10.51,33.71) | | 20.25 (10.62,32.82) | -0.30 | 0.02 (0.01 to 0.03) | |
| Qatar | 21 (11, 35) | 139 (72, 223) | 561.90 |  | 26.36 (13.66,42.74) | | 26.47 (13.78,42.67) | 0.42 | 0 (-0.01 to 0.01) | |
| Republic of Korea | 3592 (1891, 5843) | 3003 (1617, 4805) | -16.40 |  | 27.39 (14.54,44.45) | | 27.47 (14.56,44.35) | 0.29 | 0.01 (0 to 0.02) | |
| Republic of Moldova | 241 (128, 386) | 173 (92, 275) | -28.22 |  | 21.36 (11.35,34.39) | | 21.45 (11.43,34.38) | 0.42 | -0.01 (-0.02 to -0.01) | |
| Romania | 1256 (677, 2038) | 826 (439, 1346) | -34.24 |  | 22.67 (12.2,36.76) | | 22.83 (12.11,37.16) | 0.71 | 0.03 (0.02 to 0.04) | |
| Russian Federation | 7543 (4444, 11073) | 6570 (3834, 9745) | -12.90 |  | 20.47 (12.11,30.03) | | 21.84 (12.83,32.15) | 6.69 | 0.29 (0.24 to 0.34) | |
| Rwanda | 454 (243, 736) | 957 (509, 1554) | 110.79 |  | 26.51 (14.34,42.67) | | 26.31 (14.03,42.54) | -0.75 | 0.01 (0 to 0.02) | |
| Saint Kitts and Nevis | 2 (1, 3) | 3 (2, 5) | 50.00 |  | 19.8 (10.38,31.96) | | 20 (10.43,32.72) | 1.01 | 0.02 (0.01 to 0.03) | |
| Saint Lucia | 7 (4, 12) | 9 (5, 14) | 28.57 |  | 19.78 (10.37,32.19) | | 19.75 (10.4,32.58) | -0.15 | 0 (-0.01 to 0.01) | |
| Saint Vincent and the Grenadines | 6 (3, 9) | 5 (3, 9) | -16.67 |  | 19.47 (10.23,31.84) | | 19.49 (10.1,31.64) | 0.10 | 0.01 (0 to 0.02) | |
| Samoa | 11 (6, 18) | 15 (8, 24) | 36.36 |  | 28.52 (14.99,45.69) | | 28.74 (15.32,46.54) | 0.77 | 0.04 (0.03 to 0.06) | |
| San Marino | 2 (1, 3) | 2 (1, 3) | 0.00 |  | 25.19 (13.25,40.87) | | 24.98 (13.35,40.53) | -0.83 | -0.01 (-0.02 to -0.01) | |
| Sao Tome and Principe | 8 (4, 13) | 17 (9, 27) | 112.50 |  | 29.72 (16.08,47.64) | | 29.7 (16.03,47.17) | -0.07 | -0.02 (-0.02 to -0.01) | |
| Saudi Arabia | 905 (470, 1463) | 2510 (1320, 4040) | 177.35 |  | 25.49 (13.27,41.23) | | 25.45 (13.34,41.32) | -0.16 | -0.03 (-0.04 to -0.02) | |
| Senegal | 534 (285, 868) | 1201 (639, 1946) | 124.91 |  | 29.4 (15.82,47.33) | | 29.44 (15.8,47.33) | 0.14 | 0 (-0.01 to 0.01) | |
| Serbia | 523 (279, 850) | 432 (229, 704) | -17.40 |  | 22.84 (12.18,37.13) | | 22.82 (12.06,37.33) | -0.09 | 0 (-0.01 to 0.01) | |
| Seychelles | 6 (3, 9) | 7 (4, 11) | 16.67 |  | 29.02 (15.24,46.71) | | 29.36 (15.59,47) | 1.17 | 0.08 (0.05 to 0.12) | |
| Sierra Leone | 314 (168, 498) | 693 (367, 1119) | 120.70 |  | 29.19 (15.74,46.23) | | 28.8 (15.42,46.32) | -1.34 | -0.04 (-0.05 to -0.04) | |
| Singapore | 268 (143, 432) | 377 (201, 606) | 40.67 |  | 27.67 (14.76,44.73) | | 28 (14.69,45.33) | 1.19 | 0.03 (0.03 to 0.04) | |
| Slovakia | 296 (160, 478) | 263 (141, 423) | -11.15 |  | 22.85 (12.31,36.89) | | 23.09 (12.29,37.33) | 1.05 | 0.03 (0.01 to 0.04) | |
| Slovenia | 114 (60, 181) | 87 (46, 141) | -23.68 |  | 23.05 (12.03,36.92) | | 23.37 (12.24,37.97) | 1.39 | 0.01 (0 to 0.02) | |
| Solomon Islands | 22 (12, 36) | 49 (26, 79) | 122.73 |  | 27.7 (14.95,44.08) | | 27.81 (15,44.68) | 0.40 | 0.03 (0.01 to 0.06) | |
| Somalia | 451 (240, 718) | 1290 (690, 2080) | 186.03 |  | 26.29 (14.09,41.65) | | 26.03 (14.05,41.79) | -0.99 | -0.04 (-0.05 to -0.03) | |
| South Africa | 2702 (1629, 3930) | 4078 (2473, 5990) | 50.93 |  | 26.38 (15.9,38.45) | | 26.24 (15.94,38.58) | -0.53 | 0.01 (0 to 0.02) | |
| South Sudan | 370 (201, 588) | 615 (331, 993) | 66.22 |  | 26.65 (14.66,42.22) | | 26.35 (14.25,42.15) | -1.13 | -0.01 (-0.02 to 0.01) | |
| Spain | 2161 (1624, 2736) | 2110 (1502, 2751) | -2.36 |  | 22.38 (16.82,28.34) | | 22.58 (16.19,29.4) | 0.89 | 0.02 (0.01 to 0.03) | |
| Sri Lanka | 1372 (724, 2224) | 1589 (839, 2538) | 15.82 |  | 28.7 (15.17,46.49) | | 29.11 (15.33,46.49) | 1.43 | 0.11 (0.07 to 0.16) | |
| Sudan | 1220 (646, 1969) | 2880 (1527, 4677) | 136.07 |  | 24.24 (12.89,38.92) | | 24.21 (12.85,39.22) | -0.12 | -0.04 (-0.05 to -0.03) | |
| Suriname | 17 (12, 23) | 24 (17, 33) | 41.18 |  | 16.78 (11.97,22.53) | | 16.82 (12.01,22.83) | 0.24 | -0.01 (-0.01 to 0) | |
| Sweden | 506 (314, 739) | 554 (338, 822) | 9.49 |  | 24.5 (15.1,35.93) | | 24.88 (15.07,36.95) | 1.55 | 0.01 (-0.02 to 0.04) | |
| Switzerland | 442 (238, 710) | 474 (254, 761) | 7.24 |  | 25.22 (13.53,40.74) | | 25.05 (13.23,40.6) | -0.67 | 0 (-0.01 to 0.01) | |
| Syrian Arab Republic | 750 (395, 1212) | 926 (495, 1498) | 23.47 |  | 24.89 (13.17,40.1) | | 24.69 (13.11,39.67) | -0.80 | -0.05 (-0.06 to -0.04) | |
| Taiwan (Province of China) | 1595 (855, 2555) | 1533 (827, 2480) | -3.89 |  | 28 (15,44.91) | | 29.8 (15.83,48.42) | 6.43 | 0.17 (0.13 to 0.2) | |
| Tajikistan | 302 (162, 486) | 591 (315, 948) | 95.70 |  | 22.48 (12.08,36.25) | | 22.28 (11.92,35.79) | -0.89 | -0.04 (-0.06 to -0.03) | |
| Thailand | 4808 (2479, 7830) | 4306 (2299, 6933) | -10.44 |  | 28.5 (14.74,46.41) | | 28.89 (15.29,46.64) | 1.37 | 0.11 (0.07 to 0.14) | |
| Timor-Leste | 50 (27, 81) | 96 (51, 152) | 92.00 |  | 25.27 (13.56,40.36) | | 25.5 (13.63,40.55) | 0.91 | 0.12 (0.08 to 0.16) | |
| Togo | 263 (139, 431) | 639 (344, 1012) | 142.97 |  | 29.18 (15.61,47.54) | | 28.95 (15.69,45.78) | -0.79 | -0.03 (-0.03 to -0.02) | |
| Tokelau | 0 (0, 0) | 0 (0, 0) |  |  | 28.39 (15.07,45.36) | | 28.84 (15.01,47.18) | 1.59 | 0.05 (0.03 to 0.07) | |
| Tonga | 7 (4, 11) | 8 (4, 12) | 14.29 |  | 28.9 (15.27,46.21) | | 29.06 (15.34,46.64) | 0.55 | 0.02 (0 to 0.04) | |
| Trinidad and Tobago | 51 (38, 69) | 67 (39, 102) | 31.37 |  | 16.04 (12.02,21.65) | | 20.95 (11.97,31.91) | 30.61 | 0.62 (0.36 to 0.87) | |
| Tunisia | 563 (298, 904) | 730 (383, 1174) | 29.66 |  | 25.43 (13.51,40.74) | | 25.29 (13.23,40.75) | -0.55 | -0.04 (-0.05 to -0.03) | |
| Turkey | 3727 (2109, 5581) | 5144 (2946, 7701) | 38.02 |  | 24.57 (13.9,36.88) | | 24.67 (14.13,36.92) | 0.41 | -0.01 (-0.01 to 0) | |
| Turkmenistan | 220 (116, 350) | 291 (154, 467) | 32.27 |  | 22.64 (11.99,35.84) | | 22.91 (12.12,36.76) | 1.19 | 0.02 (0.01 to 0.04) | |
| Tuvalu | 1 (0, 1) | 1 (0, 1) | 0.00 |  | 27.84 (14.99,44.7) | | 28.24 (15.28,45.42) | 1.44 | 0.03 (0.01 to 0.04) | |
| Uganda | 1071 (586, 1698) | 2874 (1529, 4708) | 168.35 |  | 26.2 (14.5,41.33) | | 26.14 (14.07,42.45) | -0.23 | 0 (0 to 0.01) | |
| Ukraine | 2713 (1600, 4064) | 1949 (1110, 2975) | -28.16 |  | 21.89 (12.95,32.83) | | 21.66 (12.41,32.78) | -1.05 | -0.04 (-0.05 to -0.04) | |
| United Arab Emirates | 100 (52, 162) | 361 (193, 574) | 261.00 |  | 26.49 (13.81,43.33) | | 25.89 (13.43,41.51) | -2.27 | -0.11 (-0.12 to -0.09) | |
| United Kingdom | 2903 (1739, 4292) | 2746 (1630, 4105) | -5.41 |  | 20.57 (12.32,30.38) | | 18.33 (10.87,27.39) | -10.89 | -0.33 (-0.44 to -0.22) | |
| United Republic of Tanzania | 1675 (895, 2719) | 4084 (2176, 6575) | 143.82 |  | 26.37 (14.27,42.53) | | 26.31 (14.12,42.24) | -0.23 | 0.02 (0.01 to 0.03) | |
| United States Virgin Islands | 6 (3, 9) | 3 (2, 5) | -50.00 |  | 20.21 (10.55,32.91) | | 20.38 (10.65,33.28) | 0.84 | 0.02 (0.01 to 0.04) | |
| United States of America | 21149 (12640, 31221) | 23096 (13695, 34203) | 9.21 |  | 32.57 (19.56,47.86) | | 32.22 (19.19,47.55) | -1.07 | -0.05 (-0.07 to -0.03) | |
| Uruguay | 204 (108, 328) | 222 (118, 361) | 8.82 |  | 27.2 (14.44,43.75) | | 27.36 (14.43,44.49) | 0.59 | -0.01 (-0.03 to 0.01) | |
| Uzbekistan | 1216 (646, 1969) | 2005 (1045, 3214) | 64.88 |  | 22.53 (12.04,36.39) | | 22.51 (11.75,36.16) | -0.09 | -0.01 (-0.02 to 0) | |
| Vanuatu | 11 (6, 17) | 23 (12, 37) | 109.09 |  | 28.13 (14.79,45.21) | | 28.24 (15.11,45.22) | 0.39 | 0.02 (0 to 0.04) | |
| Venezuela (Bolivarian Republic of) | 1122 (581, 1804) | 1402 (734, 2266) | 24.96 |  | 21.79 (11.36,35.04) | | 21.64 (11.34,34.85) | -0.69 | -0.04 (-0.05 to -0.03) | |
| Viet Nam | 6001 (3180, 9578) | 7675 (3999, 12056) | 27.90 |  | 31.7 (16.84,50.64) | | 32.17 (16.86,50.34) | 1.48 | 0.11 (0.08 to 0.14) | |
| Yemen | 713 (375, 1152) | 2076 (1092, 3355) | 191.16 |  | 24.32 (12.86,39.18) | | 23.84 (12.6,38.44) | -1.97 | -0.08 (-0.08 to -0.07) | |
| Zambia | 509 (272, 817) | 1366 (722, 2184) | 168.37 |  | 26.22 (14.18,41.84) | | 26.22 (14,41.67) | 0.00 | 0.01 (0 to 0.02) | |
| Zimbabwe | 652 (344, 1048) | 1060 (571, 1698) | 62.58 |  | 25.61 (13.66,40.83) | | 25.27 (13.67,40.37) | -1.33 | -0.06 (-0.07 to -0.04) | |

**Table S4: Country-specific Prevalence of Schizophrenia among women of reproductive age in 1990 and 2021, with trends from 1990 to 2021 across 204 countries.**

| Location | Prevalence cases | | |  | Prevalence rates | | |  |
| --- | --- | --- | --- | --- | --- | --- | --- | --- |
|  | 1990_numbers(95% UI) | 2021_numbers(95% UI) | Percentage change in case(100%) | | 1990_per 100 000(95% UI) | 2021_per 100 000(95% UI) | Percentage change in ASRs(100%) | EAPC(95% CI) |
| Afghanistan | 5776 (3989, 8111) | 19477 (13277, 27718) | 237.21 | | 314.47 (220.7,434.76) | 309.89 (216.66,432.09) | -1.46 | -0.04 (-0.08 to -0.01) |
| Albania | 2426 (1682, 3416) | 2027 (1410, 2793) | -16.45 | | 311.53 (220.1,431.74) | 318.08 (220.23,439.91) | 2.10 | 0.04 (0.03 to 0.05) |
| Algeria | 17965 (12191, 25227) | 41209 (28939, 56985) | 129.38 | | 352.09 (245.61,482.69) | 348.75 (243.12,485.7) | -0.95 | -0.04 (-0.04 to -0.03) |
| American Samoa | 45 (31, 64) | 45 (32, 62) | 0.00 | | 404.68 (282.52,562.14) | 397.54 (275.31,544.97) | -1.76 | -0.04 (-0.06 to -0.03) |
| Andorra | 55 (39, 76) | 83 (60, 112) | 50.91 | | 354.83 (249.19,489.74) | 354.65 (248.52,490) | -0.05 | -0.01 (-0.02 to 0) |
| Angola | 6958 (4776, 9869) | 23415 (16064, 33276) | 236.52 | | 336.66 (236.13,468.56) | 338.6 (236.52,474.18) | 0.58 | 0.07 (0.05 to 0.1) |
| Antigua and Barbuda | 46 (31, 66) | 74 (52, 103) | 60.87 | | 293.99 (201.95,413.43) | 291.59 (203.05,407.24) | -0.82 | 0.01 (-0.01 to 0.02) |
| Argentina | 30400 (21124, 42311) | 47456 (33328, 64972) | 56.11 | | 384.78 (267.61,535.02) | 386.56 (270.18,531.36) | 0.46 | 0 (-0.01 to 0.02) |
| Armenia | 2755 (1904, 3865) | 2628 (1854, 3590) | -4.61 | | 319.44 (223.89,444.26) | 321 (223.51,443.85) | 0.49 | 0.01 (0.01 to 0.02) |
| Australia | 21153 (17679, 25189) | 30008 (25271, 35713) | 41.86 | | 462.88 (385.98,551.78) | 461.82 (386.08,552.68) | -0.23 | 0.01 (0 to 0.02) |
| Austria | 7133 (5014, 9786) | 7638 (5406, 10464) | 7.08 | | 347.72 (244.08,478.19) | 348.54 (243.38,484.46) | 0.24 | 0 (-0.01 to 0.01) |
| Azerbaijan | 5572 (3789, 7795) | 9448 (6606, 13007) | 69.56 | | 315.47 (219.97,433.52) | 318.81 (221.65,441.38) | 1.06 | 0.05 (0.03 to 0.07) |
| Bahamas | 199 (136, 284) | 317 (221, 439) | 59.30 | | 289.36 (200.14,406.85) | 287.4 (199.79,400.74) | -0.68 | -0.01 (-0.02 to -0.01) |
| Bahrain | 410 (278, 579) | 1236 (869, 1698) | 201.46 | | 365.09 (253.49,507.51) | 366.05 (256.5,504.98) | 0.26 | -0.01 (-0.02 to 0) |
| Bangladesh | 78232 (53261, 110492) | 162919 (113786, 224152) | 108.25 | | 365.2 (255.05,505.06) | 360.12 (252.81,493.19) | -1.39 | -0.06 (-0.07 to -0.05) |
| Barbados | 222 (167, 288) | 235 (165, 321) | 5.86 | | 327.44 (246.42,421.67) | 310.2 (215.64,429.22) | -5.27 | -0.05 (-0.09 to -0.02) |
| Belarus | 7841 (5415, 10893) | 7250 (5145, 9900) | -7.54 | | 298.68 (206.46,415.53) | 302.05 (209.77,419.51) | 1.13 | 0.02 (0.01 to 0.03) |
| Belgium | 8817 (6240, 12201) | 9341 (6623, 12755) | 5.94 | | 346.26 (244.21,480.94) | 345.84 (241.82,478.31) | -0.12 | 0 (-0.01 to 0) |
| Belize | 101 (69, 144) | 321 (221, 446) | 217.82 | | 277.29 (193.9,385.54) | 277.4 (192.96,382.41) | 0.04 | 0.01 (0 to 0.02) |
| Benin | 3669 (2497, 5168) | 10781 (7370, 15280) | 193.84 | | 370.36 (258.92,511.71) | 372.98 (260.8,517.51) | 0.71 | 0.01 (0 to 0.02) |
| Bermuda | 55 (38, 76) | 45 (32, 63) | -18.18 | | 303.57 (209.56,419.56) | 303.72 (211.65,425.8) | 0.05 | 0.03 (0.02 to 0.04) |
| Bhutan | 451 (306, 629) | 760 (529, 1047) | 68.51 | | 367.24 (255.71,502.23) | 368.4 (257.52,505.21) | 0.32 | -0.01 (-0.01 to 0) |
| Bolivia (Plurinational State of) | 4121 (2846, 5768) | 9130 (6326, 12800) | 121.55 | | 293.65 (205.94,405.81) | 296.74 (206.61,414.22) | 1.05 | 0.02 (0.01 to 0.03) |
| Bosnia and Herzegovina | 3624 (2492, 5035) | 2544 (1812, 3447) | -29.80 | | 310.24 (214.16,429.94) | 318.63 (222.98,438.36) | 2.70 | 0.08 (0.06 to 0.09) |
| Botswana | 932 (635, 1314) | 2366 (1651, 3292) | 153.86 | | 332.09 (232.95,459.05) | 343.63 (240.31,477.86) | 3.47 | 0.12 (0.11 to 0.14) |
| Brazil | 115126 (86959, 147690) | 195596 (149708, 247199) | 69.90 | | 314 (239.53,399.4) | 316.23 (240.75,401.57) | 0.71 | 0.02 (0 to 0.03) |
| Brunei Darussalam | 246 (167, 347) | 498 (349, 684) | 102.44 | | 386.43 (266.98,534.71) | 377.5 (262.98,520.34) | -2.31 | -0.06 (-0.07 to -0.05) |
| Bulgaria | 6674 (4705, 9120) | 4966 (3565, 6718) | -25.59 | | 307.82 (214.6,424.44) | 309.12 (216.65,425.67) | 0.42 | 0.01 (0 to 0.01) |
| Burkina Faso | 6759 (4632, 9575) | 17669 (12017, 25176) | 161.41 | | 359.73 (251.69,500.63) | 360.94 (251.46,503.58) | 0.34 | 0 (-0.01 to 0.01) |
| Burundi | 3709 (2530, 5319) | 8877 (6101, 12699) | 139.34 | | 325.79 (227.56,458.6) | 318.52 (223.82,447.46) | -2.23 | -0.06 (-0.07 to -0.05) |
| Cabo Verde | 268 (180, 377) | 593 (410, 823) | 121.27 | | 389.62 (271.51,535.34) | 395.65 (275.67,546.58) | 1.55 | 0.06 (0.05 to 0.07) |
| Cambodia | 8161 (5577, 11399) | 16390 (11363, 22998) | 100.83 | | 349.77 (243.12,482.49) | 362.06 (252.13,506.24) | 3.51 | 0.18 (0.13 to 0.22) |
| Cameroon | 7806 (5329, 11124) | 25940 (17723, 36571) | 232.31 | | 369.66 (258.65,515.47) | 364.76 (254.2,506.1) | -1.33 | -0.03 (-0.04 to -0.03) |
| Canada | 30114 (29264, 30968) | 34616 (33663, 35578) | 14.95 | | 386.5 (375.31,397.83) | 386.82 (375.68,398.12) | 0.08 | 0 (0 to 0) |
| Central African Republic | 1811 (1235, 2595) | 3826 (2615, 5356) | 111.26 | | 313.5 (218.77,441.22) | 305.92 (212.63,422.34) | -2.42 | -0.07 (-0.08 to -0.06) |
| Chad | 4289 (2947, 6122) | 12084 (8205, 17260) | 181.74 | | 361.41 (253.25,507.03) | 363.73 (253.64,507.37) | 0.64 | 0.03 (0.02 to 0.04) |
| Chile | 13571 (9409, 18696) | 19679 (13811, 27138) | 45.01 | | 390.59 (273.63,534.02) | 395.3 (275.76,548.4) | 1.21 | 0.05 (0.03 to 0.07) |
| China | 1321380 (1053588, 1620305) | 1576939 (1284051, 1898760) | 19.34 | | 432.46 (348.44,526.31) | 451.15 (362.5,548.44) | 4.32 | 0.07 (0.05 to 0.1) |
| Colombia | 25121 (17006, 35666) | 41676 (29216, 57998) | 65.90 | | 311.66 (214.98,435.7) | 312.57 (219.12,435.15) | 0.29 | 0.01 (0 to 0.02) |
| Comoros | 317 (216, 445) | 647 (448, 915) | 104.10 | | 346.37 (242.1,475.55) | 345.3 (241.21,485.15) | -0.31 | -0.01 (-0.02 to 0) |
| Congo | 1625 (1113, 2299) | 4575 (3197, 6356) | 181.54 | | 333.12 (234.08,460.4) | 333.47 (234.58,461) | 0.11 | 0.03 (0.02 to 0.05) |
| Cook Islands | 17 (12, 24) | 18 (13, 25) | 5.88 | | 408.58 (284.76,564.33) | 419.73 (290.03,587.96) | 2.73 | 0.08 (0.06 to 0.1) |
| Costa Rica | 2309 (1598, 3257) | 4281 (2970, 5973) | 85.40 | | 314.76 (221.55,437.87) | 317.44 (219.51,444.4) | 0.85 | 0.04 (0.03 to 0.05) |
| Coted'Ivoire | 8847 (6015, 12636) | 22842 (15692, 32486) | 158.19 | | 367.68 (257.75,512.93) | 367.45 (256.71,515.03) | -0.06 | -0.03 (-0.04 to -0.01) |
| Croatia | 4063 (2865, 5562) | 3195 (2258, 4348) | -21.36 | | 321.92 (225.43,443.42) | 324.89 (225.5,449.15) | 0.92 | 0.02 (0.01 to 0.03) |
| Cuba | 8443 (5774, 11859) | 7646 (5394, 10458) | -9.44 | | 288.31 (199.53,401.42) | 286.69 (199.7,397) | -0.56 | 0 (-0.03 to 0.02) |
| Cyprus | 700 (497, 954) | 1430 (1023, 1929) | 104.29 | | 346.68 (245.7,472.9) | 347.37 (243.7,476.21) | 0.20 | -0.01 (-0.02 to 0) |
| Czechia | 8781 (6202, 12055) | 8535 (6113, 11538) | -2.80 | | 327.11 (228.09,453.57) | 329.03 (229.4,455) | 0.59 | -0.01 (-0.02 to 0) |
| Democratic People's Republic of Korea | 20261 (14110, 28205) | 24617 (17413, 33688) | 21.50 | | 370.61 (260.73,511.82) | 358.91 (252.08,494.28) | -3.16 | -0.14 (-0.15 to -0.12) |
| Democratic Republic of the Congo | 24990 (16996, 35314) | 61039 (42080, 85705) | 144.25 | | 329.18 (229.15,456.34) | 319.73 (224.82,441.35) | -2.87 | -0.08 (-0.11 to -0.06) |
| Denmark | 4595 (3494, 5823) | 6186 (5589, 6909) | 34.62 | | 332.46 (250.96,423.54) | 461.26 (416.96,514.13) | 38.74 | 1.51 (1.23 to 1.79) |
| Djibouti | 293 (201, 415) | 1115 (775, 1533) | 280.55 | | 343.78 (241.56,477.78) | 342.18 (238.23,470.17) | -0.47 | 0.01 (-0.01 to 0.02) |
| Dominica | 44 (30, 62) | 46 (32, 64) | 4.55 | | 282.39 (197.28,392.97) | 280.52 (194.6,386) | -0.66 | 0 (-0.01 to 0.01) |
| Dominican Republic | 4737 (3233, 6677) | 8170 (5647, 11403) | 72.47 | | 279.35 (195.4,385.75) | 283.75 (196.9,394.91) | 1.58 | 0.06 (0.05 to 0.07) |
| Ecuador | 6990 (4769, 9883) | 14299 (9867, 19932) | 104.56 | | 305.14 (212.28,425.11) | 304.89 (211.09,423.88) | -0.08 | 0 (0 to 0.01) |
| Egypt | 41920 (28838, 58503) | 88169 (61085, 121780) | 110.33 | | 343.7 (239.54,474.95) | 347.12 (241.89,477.3) | 1.00 | 0 (-0.01 to 0.01) |
| El Salvador | 3513 (2398, 4947) | 5374 (3741, 7430) | 52.97 | | 301.42 (210.39,416.32) | 304.05 (212.54,419.08) | 0.87 | 0.03 (0.02 to 0.04) |
| Equatorial Guinea | 289 (200, 414) | 1185 (821, 1657) | 310.03 | | 325.52 (229.17,457.84) | 353.19 (248.73,486.58) | 8.50 | 0.38 (0.31 to 0.45) |
| Eritrea | 2324 (1590, 3280) | 5173 (3549, 7271) | 122.59 | | 330.79 (230.94,458.5) | 333.98 (232.42,463.8) | 0.96 | 0.03 (0.01 to 0.05) |
| Estonia | 1215 (859, 1665) | 954 (676, 1297) | -21.48 | | 302.82 (212.26,418.29) | 308.28 (214.35,426.73) | 1.80 | 0.04 (0.04 to 0.05) |
| Eswatini | 557 (373, 796) | 995 (680, 1407) | 78.64 | | 329.7 (227.05,460.23) | 332.75 (231.08,465.31) | 0.93 | 0.06 (0.04 to 0.07) |
| Ethiopia | 34115 (25458, 44015) | 84507 (63527, 108690) | 147.71 | | 339.49 (257.9,432.2) | 342.18 (261.22,434.14) | 0.79 | 0.06 (0.05 to 0.08) |
| Fiji | 713 (486, 997) | 893 (625, 1240) | 25.25 | | 383.02 (264.87,530.21) | 390.11 (272.97,542.31) | 1.85 | 0.06 (0.04 to 0.07) |
| Finland | 5118 (3924, 6384) | 4158 (2990, 5574) | -18.76 | | 369.63 (281.16,464.55) | 337.5 (240.06,456.69) | -8.69 | -0.32 (-0.41 to -0.24) |
| France | 50906 (35692, 69898) | 51966 (37098, 71270) | 2.08 | | 341.71 (238.88,470.55) | 341.16 (240.61,473.49) | -0.16 | -0.01 (-0.02 to 0.01) |
| Gabon | 678 (461, 965) | 1594 (1103, 2232) | 135.10 | | 351.56 (246.06,487.96) | 350.27 (245.97,484.58) | -0.37 | -0.02 (-0.03 to -0.01) |
| Gambia | 742 (502, 1061) | 2006 (1370, 2813) | 170.35 | | 371.99 (259.46,519.28) | 365.91 (255.95,504.03) | -1.63 | -0.04 (-0.05 to -0.03) |
| Georgia | 14660 (11025, 18988) | 16760 (12787, 21246) | 14.32 | | 526.58 (396.73,681.02) | 985.54 (742.04,1262.9) | 87.16 | 2.11 (2.06 to 2.15) |
| Germany | 70002 (49595, 96763) | 64432 (45890, 87954) | -7.96 | | 339.01 (239.54,470.29) | 341.84 (240.22,472.63) | 0.83 | 0.01 (-0.03 to 0.04) |
| Ghana | 11780 (7963, 16734) | 32573 (22497, 45330) | 176.51 | | 369.9 (256.04,516.01) | 374.15 (261.83,515.37) | 1.15 | 0.06 (0.05 to 0.06) |
| Greece | 8963 (6323, 12291) | 8564 (6149, 11614) | -4.45 | | 346.47 (243.34,477.1) | 346.34 (242.93,480.13) | -0.04 | -0.01 (-0.02 to 0) |
| Greenland | 69 (48, 93) | 61 (44, 82) | -11.59 | | 459.96 (325.27,619.45) | 466.58 (331.27,625.92) | 1.44 | 0.08 (0.05 to 0.11) |
| Grenada | 49 (33, 70) | 73 (51, 100) | 48.98 | | 278.05 (193.05,387.32) | 282.51 (198.36,388.65) | 1.60 | 0.04 (0.04 to 0.05) |
| Guam | 145 (99, 205) | 155 (108, 215) | 6.90 | | 423.45 (292.71,591.47) | 424.81 (295.58,591.95) | 0.32 | 0.03 (0.01 to 0.04) |
| Guatemala | 4808 (3289, 6846) | 12278 (8523, 17043) | 155.37 | | 293.95 (205.07,411.28) | 296.03 (208.06,406.86) | 0.71 | 0.01 (0 to 0.02) |
| Guinea | 4676 (3209, 6563) | 10943 (7502, 15302) | 134.02 | | 365.57 (254.81,507.17) | 363.53 (254.59,499.74) | -0.56 | -0.04 (-0.05 to -0.03) |
| Guinea-Bissau | 746 (513, 1064) | 1731 (1191, 2447) | 132.04 | | 359.12 (252.28,502.06) | 357.84 (251.03,498.28) | -0.36 | -0.03 (-0.04 to -0.02) |
| Guyana | 498 (340, 710) | 544 (376, 759) | 9.24 | | 268.78 (187.88,375.39) | 273.19 (190.19,378.74) | 1.64 | 0.05 (0.04 to 0.06) |
| Haiti | 3640 (2498, 5086) | 8747 (6118, 12286) | 140.30 | | 256.13 (178.77,353.02) | 251.72 (176.96,351.84) | -1.72 | -0.06 (-0.06 to -0.05) |
| Honduras | 2735 (1875, 3851) | 7902 (5449, 11104) | 188.92 | | 293.21 (205.3,405.29) | 293.28 (204.82,407.94) | 0.02 | -0.02 (-0.03 to -0.01) |
| Hungary | 8451 (5922, 11536) | 7641 (5495, 10333) | -9.58 | | 315.75 (218.22,435.54) | 320.61 (225.03,443.03) | 1.54 | 0.02 (0.02 to 0.03) |
| Iceland | 226 (157, 312) | 296 (211, 409) | 30.97 | | 349.83 (244.72,481.14) | 350.31 (247.57,487.52) | 0.14 | -0.01 (-0.01 to 0) |
| India | 682791 (518819, 863814) | 1394215 (1062756, 1768270) | 104.19 | | 360.27 (276.04,452.67) | 373.27 (285.24,472.34) | 3.61 | 0.14 (0.1 to 0.18) |
| Indonesia | 168096 (126626, 216052) | 296331 (227694, 376547) | 76.29 | | 375.27 (286.12,478) | 383.56 (293.69,488.82) | 2.21 | 0.14 (0.1 to 0.19) |
| Iran (Islamic Republic of) | 39904 (30039, 51125) | 91405 (70958, 114393) | 129.06 | | 355.99 (272.68,449.4) | 358.63 (275.53,452.94) | 0.74 | 0.01 (0 to 0.02) |
| Iraq | 12361 (8418, 17257) | 34955 (24148, 48279) | 182.78 | | 346.46 (242.01,473.4) | 346.31 (241.43,475.25) | -0.04 | 0.01 (0 to 0.02) |
| Ireland | 3769 (2808, 4928) | 5542 (3926, 7531) | 47.04 | | 436.59 (325.57,570.09) | 437.41 (303.5,605.1) | 0.19 | 0.14 (0.06 to 0.23) |
| Israel | 3908 (2747, 5412) | 7445 (5227, 10315) | 90.51 | | 329.05 (231.91,454.15) | 327.48 (228.54,456.16) | -0.48 | -0.01 (-0.03 to 0) |
| Italy | 44391 (34145, 56182) | 41991 (32769, 52408) | -5.41 | | 303.3 (232.77,384.7) | 304.8 (233.66,386.32) | 0.49 | 0 (0 to 0.01) |
| Jamaica | 1519 (1029, 2156) | 2202 (1540, 3064) | 44.96 | | 282.2 (195.42,392.96) | 281.9 (197.7,391.63) | -0.11 | -0.01 (-0.01 to 0) |
| Japan | 126896 (98058, 158291) | 107192 (83225, 133575) | -15.53 | | 376.29 (288.04,473.64) | 384.98 (294.28,486.63) | 2.31 | 0.2 (0.13 to 0.27) |
| Jordan | 2479 (1673, 3519) | 10398 (7224, 14494) | 319.44 | | 352.38 (245.47,487.95) | 352.55 (246.85,488.02) | 0.05 | -0.02 (-0.03 to -0.02) |
| Kazakhstan | 12699 (8824, 17694) | 16046 (11250, 22080) | 26.36 | | 312.76 (219.43,432.72) | 317.24 (220.52,439.95) | 1.43 | 0.07 (0.06 to 0.08) |
| Kenya | 14838 (11071, 19121) | 41074 (30872, 52630) | 176.82 | | 340.54 (260.08,430.95) | 341.78 (260.38,433.03) | 0.36 | 0.01 (0 to 0.02) |
| Kiribati | 63 (43, 90) | 111 (77, 155) | 76.19 | | 357.86 (247.71,500.95) | 355.47 (247.83,493.89) | -0.67 | -0.02 (-0.04 to 0) |
| Kuwait | 1514 (1027, 2116) | 6292 (4505, 8461) | 315.59 | | 374.54 (258.74,516.23) | 374.7 (263.29,512.76) | 0.04 | -0.02 (-0.04 to -0.01) |
| Kyrgyzstan | 3004 (2048, 4233) | 5329 (3674, 7341) | 77.40 | | 308.79 (215.24,428.12) | 306.23 (211.83,420.9) | -0.83 | -0.03 (-0.05 to -0.02) |
| Lao People's Democratic Republic | 3106 (2101, 4391) | 7137 (4910, 9984) | 129.78 | | 350.08 (241.69,487.55) | 365.74 (253.74,508.14) | 4.47 | 0.23 (0.19 to 0.28) |
| Latvia | 2044 (1425, 2805) | 1320 (936, 1792) | -35.42 | | 300.43 (207.84,415.06) | 303.4 (210.44,420.03) | 0.99 | 0.04 (0.03 to 0.05) |
| Lebanon | 2559 (1779, 3541) | 5728 (4031, 7896) | 123.84 | | 357.22 (251.04,490.23) | 358.33 (250.34,498.29) | 0.31 | 0 (-0.01 to 0) |
| Lesotho | 1085 (751, 1516) | 1491 (1025, 2126) | 37.42 | | 309.97 (217.35,429.32) | 317.62 (221.99,446.47) | 2.47 | 0.1 (0.09 to 0.12) |
| Liberia | 1823 (1227, 2614) | 4595 (3178, 6474) | 152.06 | | 365.2 (252.92,511.87) | 357.13 (250.1,497.89) | -2.21 | -0.03 (-0.04 to -0.01) |
| Libya | 2755 (1874, 3883) | 7223 (5070, 9890) | 162.18 | | 360.29 (252.93,494.96) | 350.38 (243.9,483.13) | -2.75 | -0.12 (-0.14 to -0.1) |
| Lithuania | 2853 (2008, 3901) | 1919 (1356, 2643) | -32.74 | | 300.85 (211.16,412.74) | 306.42 (213.11,427.61) | 1.85 | 0.04 (0.04 to 0.05) |
| Luxembourg | 363 (256, 502) | 617 (435, 840) | 69.97 | | 349.39 (244.74,484.78) | 353.24 (245.42,488.29) | 1.10 | 0.04 (0.02 to 0.05) |
| Madagascar | 7930 (5393, 11298) | 21713 (14823, 30844) | 173.81 | | 335.78 (234.3,467.53) | 333.57 (232.17,465.08) | -0.66 | -0.03 (-0.04 to -0.01) |
| Malawi | 6280 (4254, 8957) | 13784 (9328, 19648) | 119.49 | | 318.5 (221.53,445.02) | 317.56 (219.93,443.69) | -0.30 | 0 (-0.01 to 0.01) |
| Malaysia | 17011 (11629, 23783) | 34878 (24009, 48846) | 105.03 | | 398.28 (276.61,550.26) | 409.23 (282.61,571.67) | 2.75 | 0.16 (0.11 to 0.2) |
| Maldives | 160 (108, 227) | 490 (344, 674) | 206.25 | | 384.97 (268.25,533.07) | 397.28 (276.52,550.05) | 3.20 | 0.18 (0.14 to 0.21) |
| Mali | 6272 (4287, 8856) | 17192 (11638, 24487) | 174.11 | | 359.17 (250.11,499.05) | 364.57 (253.25,507.75) | 1.50 | 0.03 (0.02 to 0.05) |
| Malta | 352 (249, 481) | 372 (265, 506) | 5.68 | | 345.93 (241.71,477.38) | 349.41 (244.84,483.49) | 1.01 | 0.02 (0.01 to 0.03) |
| Marshall Islands | 32 (21, 45) | 53 (36, 72) | 65.63 | | 362.38 (251.18,507.07) | 363.26 (253.02,497.38) | 0.24 | 0.02 (0 to 0.03) |
| Mauritania | 1588 (1069, 2243) | 3664 (2513, 5182) | 130.73 | | 377.16 (260.12,522.96) | 380.25 (265.98,528.12) | 0.82 | 0.01 (0.01 to 0.02) |
| Mauritius | 1164 (795, 1633) | 1321 (940, 1797) | 13.49 | | 393.75 (272.38,547.73) | 402.74 (284.16,551.36) | 2.28 | 0.15 (0.1 to 0.19) |
| Mexico | 62742 (47109, 80585) | 114717 (87413, 145626) | 82.84 | | 321.75 (245.3,407.82) | 322.45 (245.24,410.09) | 0.22 | 0.01 (0 to 0.02) |
| Micronesia (Federated States of) | 75 (51, 107) | 90 (62, 127) | 20.00 | | 361.99 (251.91,505.87) | 364.01 (253.16,509.64) | 0.56 | 0.01 (-0.01 to 0.02) |
| Monaco | 29 (20, 40) | 29 (21, 40) | 0.00 | | 363.46 (252.31,506.68) | 366.22 (253.77,513.54) | 0.76 | 0.01 (0 to 0.02) |
| Mongolia | 1357 (925, 1908) | 2757 (1948, 3774) | 103.17 | | 300.47 (210.77,412.68) | 307.63 (215.8,423.73) | 2.38 | 0.08 (0.07 to 0.1) |
| Montenegro | 495 (342, 690) | 489 (347, 665) | -1.21 | | 316.29 (219,440.49) | 316.89 (221.8,436.44) | 0.19 | 0.01 (0 to 0.02) |
| Morocco | 19666 (13510, 27690) | 33553 (23472, 46597) | 70.61 | | 340.75 (238.59,471.4) | 341.17 (238.03,474.95) | 0.12 | -0.02 (-0.03 to -0.01) |
| Mozambique | 8845 (6068, 12467) | 21010 (14346, 29924) | 137.54 | | 310.12 (216.09,431.1) | 318.15 (222.86,444.78) | 2.59 | 0.13 (0.11 to 0.15) |
| Myanmar | 33678 (23113, 47647) | 54812 (38281, 75894) | 62.75 | | 347.69 (243.48,483.17) | 365.22 (255.18,505.48) | 5.04 | 0.27 (0.23 to 0.32) |
| Namibia | 995 (678, 1427) | 2167 (1501, 3044) | 117.79 | | 337.65 (236.21,473.16) | 342.5 (239.83,477.06) | 1.44 | 0.07 (0.06 to 0.09) |
| Nauru | 9 (6, 13) | 10 (7, 15) | 11.11 | | 392.93 (274.22,545.37) | 382.01 (265.15,530.05) | -2.78 | -0.07 (-0.14 to 0) |
| Nepal | 15012 (10208, 20802) | 30975 (21300, 42375) | 106.33 | | 359.12 (248.38,490.93) | 354.87 (246.31,482.08) | -1.18 | -0.06 (-0.07 to -0.05) |
| Netherlands | 20155 (15824, 25285) | 18861 (14279, 24177) | -6.42 | | 490.2 (383.76,616.99) | 481.79 (361.2,622.89) | -1.72 | -0.09 (-0.12 to -0.06) |
| New Zealand | 4279 (3250, 5451) | 5991 (4595, 7566) | 40.01 | | 470.13 (357.12,598.8) | 474.52 (362.03,601.79) | 0.93 | 0 (-0.02 to 0.01) |
| Nicaragua | 2411 (1638, 3412) | 5477 (3767, 7635) | 127.17 | | 306.02 (213.42,423.97) | 305.01 (210.72,423.69) | -0.33 | 0 (-0.02 to 0.01) |
| Niger | 5572 (3814, 7919) | 15971 (10884, 22411) | 186.63 | | 363.67 (254.79,506.7) | 360.18 (252.66,493.35) | -0.96 | -0.04 (-0.05 to -0.03) |
| Nigeria | 72161 (54356, 92788) | 210415 (158855, 269966) | 191.59 | | 415.87 (319.91,525.85) | 417.33 (320.03,528.57) | 0.35 | 0.03 (0.02 to 0.04) |
| Niue | 2 (1, 3) | 2 (1, 2) | 0.00 | | 392.21 (275.6,539.2) | 398.34 (278.04,554.36) | 1.56 | 0.07 (0.06 to 0.09) |
| North Macedonia | 1612 (1121, 2246) | 1851 (1309, 2531) | 14.83 | | 315.19 (219.18,439.17) | 316.36 (220.1,438.37) | 0.37 | 0.01 (0 to 0.02) |
| Northern Mariana Islands | 58 (40, 81) | 49 (35, 67) | -15.52 | | 427.5 (298.74,592.23) | 418.19 (290.73,578.89) | -2.18 | -0.11 (-0.13 to -0.08) |
| Norway | 3945 (3026, 4979) | 4713 (3633, 5937) | 19.47 | | 361.31 (276.11,457.23) | 360.61 (275.69,457.34) | -0.19 | 0 (-0.01 to 0.01) |
| Oman | 1125 (768, 1575) | 3902 (2741, 5371) | 246.84 | | 358.99 (249.86,494.56) | 360.37 (252.08,498.7) | 0.38 | 0 (0 to 0.01) |
| Pakistan | 78460 (58879, 101730) | 212573 (160278, 270935) | 170.93 | | 373.03 (283.55,478.56) | 367.38 (279.07,465.14) | -1.51 | -0.07 (-0.08 to -0.06) |
| Palau | 15 (11, 22) | 16 (11, 21) | 6.67 | | 391.2 (275.52,541.69) | 397.1 (277.55,553.52) | 1.51 | 0.04 (0.03 to 0.06) |
| Palestine | 1336 (903, 1900) | 4260 (2905, 5958) | 218.86 | | 350.87 (244.5,488.27) | 350.47 (243.24,483.41) | -0.11 | -0.04 (-0.06 to -0.02) |
| Panama | 1759 (1219, 2470) | 3386 (2340, 4682) | 92.50 | | 310.83 (218.81,430.49) | 316.98 (219.17,438.03) | 1.98 | 0.06 (0.05 to 0.07) |
| Papua New Guinea | 3227 (2197, 4584) | 9431 (6510, 13119) | 192.25 | | 364.17 (253.3,508.37) | 369.6 (257.3,510.52) | 1.49 | 0.03 (0.01 to 0.05) |
| Paraguay | 2661 (1824, 3714) | 5806 (4008, 8104) | 118.19 | | 307.81 (214.45,423.29) | 310.74 (215.71,431.71) | 0.95 | 0 (-0.01 to 0.02) |
| Peru | 15006 (10308, 21020) | 30341 (21170, 41699) | 102.19 | | 304.06 (212.84,419.34) | 308.81 (215.44,424.68) | 1.56 | 0.03 (0.02 to 0.04) |
| Philippines | 54816 (41345, 70462) | 111316 (84733, 141177) | 103.07 | | 384.16 (293.84,488.46) | 389.24 (297.83,491.79) | 1.32 | 0.11 (0.07 to 0.15) |
| Poland | 31838 (24428, 40113) | 32667 (25284, 40899) | 2.60 | | 321.34 (245.28,406.94) | 326.71 (249.22,414.36) | 1.67 | 0.05 (0.04 to 0.06) |
| Portugal | 8668 (6129, 11843) | 9095 (6505, 12375) | 4.93 | | 340.32 (240.05,465.98) | 344.39 (240.34,479.66) | 1.20 | 0.01 (0 to 0.02) |
| Puerto Rico | 2824 (1982, 3954) | 2346 (1662, 3216) | -16.93 | | 296.23 (207.92,414.79) | 296.97 (207.9,411.22) | 0.25 | 0.02 (0.01 to 0.03) |
| Qatar | 305 (211, 422) | 2398 (1676, 3278) | 686.23 | | 375.78 (260.88,519.3) | 378.94 (261.8,523.88) | 0.84 | 0.01 (0 to 0.03) |
| Republic of Korea | 45854 (31667, 64018) | 49665 (35537, 67858) | 8.31 | | 377.16 (263.75,521.56) | 379.19 (265.84,527.16) | 0.54 | 0.01 (0 to 0.02) |
| Republic of Moldova | 3412 (2370, 4690) | 2952 (2105, 4015) | -13.48 | | 293.09 (203.39,404.2) | 294.99 (205.77,408.34) | 0.65 | 0 (-0.01 to 0.01) |
| Romania | 17660 (12308, 24386) | 14167 (10142, 19152) | -19.78 | | 313.83 (217.99,433.93) | 317.72 (222.01,437.99) | 1.24 | 0.05 (0.04 to 0.06) |
| Russian Federation | 110701 (84583, 139783) | 115948 (89576, 145066) | 4.74 | | 284.07 (216.46,359.5) | 303.77 (231.12,385.96) | 6.93 | 0.3 (0.25 to 0.36) |
| Rwanda | 4729 (3200, 6755) | 10818 (7470, 15263) | 128.76 | | 329.82 (229.09,460.65) | 331.44 (232.02,461.6) | 0.49 | 0.06 (0.04 to 0.08) |
| Saint Kitts and Nevis | 26 (18, 37) | 47 (33, 66) | 80.77 | | 284.84 (198.67,396.52) | 289.31 (202.44,404.49) | 1.57 | 0.04 (0.04 to 0.05) |
| Saint Lucia | 87 (59, 123) | 137 (97, 191) | 57.47 | | 284.19 (198.32,394.06) | 284.49 (197.84,399.34) | 0.11 | 0 (-0.01 to 0.01) |
| Saint Vincent and the Grenadines | 64 (44, 91) | 79 (56, 110) | 23.44 | | 276.22 (194.36,383.21) | 278.58 (194.56,388.17) | 0.85 | 0.04 (0.03 to 0.05) |
| Samoa | 122 (82, 171) | 174 (120, 242) | 42.62 | | 376.44 (261.66,518.46) | 381.03 (266.53,524.11) | 1.22 | 0.06 (0.05 to 0.08) |
| San Marino | 22 (15, 31) | 28 (20, 38) | 27.27 | | 354.14 (247.62,490.43) | 351.89 (245.64,486.26) | -0.64 | -0.02 (-0.03 to -0.01) |
| Sao Tome and Principe | 84 (57, 118) | 200 (138, 276) | 138.10 | | 382.62 (267.24,523.91) | 384.17 (269.14,525.21) | 0.41 | 0.01 (0 to 0.02) |
| Saudi Arabia | 10352 (7017, 14575) | 39345 (27592, 54195) | 280.07 | | 357.07 (248.06,493.72) | 358.14 (249.28,497.78) | 0.30 | -0.03 (-0.04 to -0.01) |
| Senegal | 5643 (3847, 8004) | 13468 (9234, 19095) | 138.67 | | 375.19 (262.64,520.54) | 377.64 (263.73,527) | 0.65 | 0.01 (0.01 to 0.02) |
| Serbia | 7606 (5271, 10466) | 6946 (4883, 9525) | -8.68 | | 316.23 (218.15,436.88) | 316.62 (219.01,440.34) | 0.12 | 0.01 (0 to 0.02) |
| Seychelles | 67 (45, 94) | 102 (72, 139) | 52.24 | | 397.77 (274.88,551.41) | 404.91 (281,558.03) | 1.80 | 0.1 (0.07 to 0.14) |
| Sierra Leone | 3362 (2308, 4748) | 7356 (5033, 10444) | 118.80 | | 365.67 (257.21,507.5) | 360.73 (252.67,502.12) | -1.35 | -0.05 (-0.06 to -0.04) |
| Singapore | 3652 (2531, 5082) | 6750 (4806, 9134) | 84.83 | | 384.8 (268.23,532.99) | 390.99 (271.95,540.34) | 1.61 | 0.05 (0.04 to 0.06) |
| Slovakia | 4351 (3064, 5974) | 4595 (3285, 6288) | 5.61 | | 318.41 (223.07,439.2) | 322.85 (225.54,449.5) | 1.39 | 0.04 (0.03 to 0.05) |
| Slovenia | 1667 (1162, 2296) | 1551 (1100, 2121) | -6.96 | | 322.38 (223.73,445.51) | 328.21 (227,457.93) | 1.81 | 0.02 (0.01 to 0.03) |
| Solomon Islands | 230 (157, 324) | 584 (404, 821) | 153.91 | | 355.39 (248.78,491) | 357.36 (249.03,498.08) | 0.55 | 0.02 (-0.01 to 0.04) |
| Somalia | 4895 (3332, 6918) | 13055 (8895, 18580) | 166.70 | | 313.49 (216.13,438.8) | 307.23 (213.65,430.11) | -2.00 | -0.08 (-0.1 to -0.06) |
| South Africa | 30375 (22852, 38938) | 55109 (42274, 70075) | 81.43 | | 345.18 (263.32,437.34) | 345.45 (264.63,439.56) | 0.08 | 0.04 (0.03 to 0.05) |
| South Sudan | 3832 (2627, 5374) | 6899 (4771, 9694) | 80.04 | | 343.72 (242.44,471.14) | 334.04 (233.76,463.92) | -2.82 | -0.06 (-0.08 to -0.03) |
| Spain | 29739 (26528, 32971) | 35566 (32007, 39238) | 19.59 | | 310.68 (277.24,344.27) | 311.47 (277.35,348.05) | 0.25 | 0.01 (0 to 0.01) |
| Sri Lanka | 17300 (12024, 24089) | 23015 (16095, 31638) | 33.03 | | 388.57 (272.63,537.1) | 399.74 (277.4,552.75) | 2.87 | 0.16 (0.12 to 0.21) |
| Sudan | 13751 (9367, 19303) | 34997 (24103, 49206) | 154.51 | | 326.89 (227.63,449.7) | 330.33 (230.86,458.82) | 1.05 | 0.01 (-0.01 to 0.02) |
| Suriname | 220 (174, 271) | 361 (289, 445) | 64.09 | | 243.68 (193.89,298.59) | 244.54 (195.15,301.33) | 0.35 | 0.01 (0.01 to 0.02) |
| Sweden | 6994 (5450, 8845) | 7524 (5830, 9558) | 7.58 | | 312.79 (241.14,399.33) | 311.41 (238.87,398.69) | -0.44 | -0.04 (-0.05 to -0.03) |
| Switzerland | 6686 (4716, 9195) | 7750 (5533, 10548) | 15.91 | | 353.72 (247.7,489.96) | 352.38 (247.48,487.24) | -0.38 | -0.01 (-0.02 to 0) |
| Syrian Arab Republic | 8147 (5553, 11514) | 12309 (8678, 17097) | 51.09 | | 343.46 (240.89,474.61) | 342.73 (240.51,475.22) | -0.21 | -0.03 (-0.05 to -0.02) |
| Taiwan (Province of China) | 20591 (14502, 28295) | 25172 (17827, 34122) | 22.25 | | 375.62 (267.06,512.45) | 403.13 (279.38,556.91) | 7.32 | 0.19 (0.15 to 0.22) |
| Tajikistan | 3243 (2201, 4579) | 7578 (5249, 10640) | 133.67 | | 306.34 (214.96,421.45) | 302.05 (211.17,421.75) | -1.40 | -0.05 (-0.07 to -0.03) |
| Thailand | 59427 (40157, 83721) | 69062 (48881, 94649) | 16.21 | | 385.28 (264.25,537.16) | 395.16 (274.47,549.82) | 2.56 | 0.15 (0.11 to 0.19) |
| Timor-Leste | 584 (397, 824) | 1042 (706, 1465) | 78.42 | | 328.84 (228.02,456.56) | 335.75 (232.95,463.27) | 2.10 | 0.18 (0.14 to 0.22) |
| Togo | 2762 (1881, 3959) | 7459 (5157, 10367) | 170.06 | | 364.85 (254.28,511.08) | 362.24 (253.06,499.59) | -0.72 | -0.03 (-0.03 to -0.03) |
| Tokelau | 1 (1, 2) | 1 (1, 2) | 0.00 | | 373.16 (261.82,518.21) | 383.18 (265.69,538.42) | 2.69 | 0.08 (0.07 to 0.1) |
| Tonga | 77 (52, 108) | 94 (64, 131) | 22.08 | | 386.92 (269.74,535.17) | 389.76 (269,540.05) | 0.73 | 0.03 (0.01 to 0.04) |
| Trinidad and Tobago | 699 (563, 886) | 1099 (794, 1468) | 57.22 | | 235.17 (190.01,297.03) | 301.57 (215.43,406.79) | 28.23 | 0.59 (0.37 to 0.82) |
| Tunisia | 6631 (4538, 9318) | 11636 (8212, 15883) | 75.48 | | 354.19 (247.37,489.5) | 354.79 (247.18,489.96) | 0.17 | -0.02 (-0.03 to -0.01) |
| Turkey | 45829 (34311, 59071) | 79012 (60416, 100367) | 72.41 | | 347.45 (263.83,442.56) | 350.85 (266.59,448.08) | 0.98 | 0.01 (0 to 0.01) |
| Turkmenistan | 2482 (1687, 3484) | 3953 (2748, 5436) | 59.27 | | 309.68 (216.43,426.07) | 315.5 (219.75,433.25) | 1.88 | 0.06 (0.03 to 0.08) |
| Tuvalu | 9 (6, 12) | 10 (7, 14) | 11.11 | | 360.36 (252.76,499.29) | 368.96 (258.11,511.81) | 2.39 | 0.04 (0.03 to 0.06) |
| Uganda | 10396 (7044, 14863) | 29390 (19910, 42478) | 182.70 | | 319.89 (224.14,445.46) | 325.76 (226.74,460.29) | 1.84 | 0.08 (0.06 to 0.09) |
| Ukraine | 40097 (30597, 51140) | 34450 (26402, 43433) | -14.08 | | 302.48 (229.72,387.56) | 299.77 (225.84,383.19) | -0.90 | -0.03 (-0.04 to -0.03) |
| United Arab Emirates | 1280 (880, 1800) | 7299 (5209, 9802) | 470.23 | | 376.32 (263.33,521.9) | 367.09 (253.13,506.65) | -2.45 | -0.12 (-0.13 to -0.1) |
| United Kingdom | 45806 (35710, 57297) | 43283 (33572, 54618) | -5.51 | | 310.23 (241.26,388.93) | 260.61 (200.39,331.32) | -15.99 | -0.48 (-0.64 to -0.32) |
| United Republic of Tanzania | 16919 (11424, 24206) | 44538 (30499, 62535) | 163.24 | | 328.1 (228.17,457.91) | 332.74 (232.44,460.13) | 1.41 | 0.08 (0.06 to 0.09) |
| United States Virgin Islands | 87 (61, 119) | 55 (39, 75) | -36.78 | | 295.12 (205.98,409.93) | 298.13 (207.37,413.62) | 1.02 | 0.03 (0.01 to 0.05) |
| United States of America | 372212 (290276, 461110) | 412495 (322294, 511118) | 10.82 | | 528.18 (410.6,656.11) | 521.39 (405.31,648.71) | -1.29 | -0.05 (-0.07 to -0.02) |
| Uruguay | 2905 (2022, 4009) | 3365 (2368, 4625) | 15.83 | | 386.99 (269.23,534.37) | 387.91 (270.9,536.91) | 0.24 | 0 (-0.02 to 0.02) |
| Uzbekistan | 13511 (9234, 19213) | 28548 (19989, 39395) | 111.29 | | 305.95 (215.35,425.03) | 307.66 (215.01,425.78) | 0.56 | 0.02 (0 to 0.03) |
| Vanuatu | 118 (79, 167) | 275 (189, 383) | 133.05 | | 366.14 (250.94,511.08) | 366.78 (254.97,506.79) | 0.17 | 0.02 (0.01 to 0.04) |
| Venezuela (Bolivarian Republic of) | 14065 (9616, 19656) | 22232 (15651, 30269) | 58.07 | | 312.67 (217.22,430.77) | 309.47 (215.4,425.34) | -1.02 | -0.03 (-0.04 to -0.02) |
| Viet Nam | 69957 (47807, 96905) | 121813 (87076, 163045) | 74.13 | | 437.23 (305.93,595.43) | 450.79 (318.87,607.86) | 3.10 | 0.16 (0.13 to 0.19) |
| Yemen | 8170 (5623, 11472) | 25194 (17318, 35249) | 208.37 | | 328.76 (231.39,453.72) | 322.51 (224.47,446.91) | -1.90 | -0.07 (-0.08 to -0.05) |
| Zambia | 4980 (3348, 7100) | 14537 (9964, 20485) | 191.91 | | 326 (226.15,452.56) | 331.51 (232.57,457.73) | 1.69 | 0.08 (0.06 to 0.1) |
| Zimbabwe | 6643 (4452, 9426) | 11808 (8127, 16553) | 77.75 | | 323.63 (223.61,448.54) | 314.72 (219.71,435.78) | -2.75 | -0.13 (-0.15 to -0.1) |

**Table S5: Country-specific DALYs due to Schizophrenia among women of reproductive age in 1990 and 2021, with trends from 1990 to 2021 across 204 countries.**

| Location | DALY cases | | |  | DALY rates | | | |  |
| --- | --- | --- | --- | --- | --- | --- | --- | --- | --- |
|  | 1990_numbers(95% UI) | 2021_numbers(95% UI) | Percentage change in case(100%) | 1990_per 100 000(95% UI) | | 2021_per 100 000(95% UI) | Percentage change in ASRs(100%) | EAPC(95% CI) | |
| Afghanistan | 3607 (2084, 5615) | 12216 (6888, 19507) | 238.67 | 195.61 (116.65,297.34) | | 193.26 (112.8,301.37) | -1.20 | -0.01 (-0.05 to 0.02) | |
| Albania | 1575 (924, 2406) | 1309 (750, 1972) | -16.89 | 201.4 (120.54,303.05) | | 205.61 (117.2,311.12) | 2.09 | 0.06 (0.05 to 0.07) | |
| Algeria | 11435 (6724, 17868) | 26146 (15797, 39947) | 128.65 | 222.78 (135.18,338.89) | | 221.63 (132.62,341.14) | -0.52 | -0.03 (-0.04 to -0.02) | |
| American Samoa | 30 (17, 46) | 29 (17, 45) | -3.33 | 263.5 (157.34,401.61) | | 255.3 (150.71,394.16) | -3.11 | -0.08 (-0.09 to -0.06) | |
| Andorra | 35 (21, 53) | 53 (32, 78) | 51.43 | 227.3 (133.88,341.18) | | 225.4 (133.31,343.61) | -0.84 | -0.02 (-0.03 to -0.01) | |
| Angola | 4422 (2535, 6883) | 14819 (8590, 23285) | 235.12 | 213.3 (124.8,326.61) | | 213.61 (126.61,329.51) | 0.15 | 0.09 (0.06 to 0.13) | |
| Antigua and Barbuda | 30 (17, 47) | 47 (28, 73) | 56.67 | 189.71 (108.69,296.87) | | 186.65 (109.87,291.4) | -1.61 | 0 (-0.02 to 0.02) | |
| Argentina | 19532 (11643, 29561) | 30066 (17959, 45142) | 53.93 | 247.18 (147.6,373.73) | | 245.09 (145.67,369.61) | -0.85 | 0 (-0.02 to 0.02) | |
| Armenia | 1794 (1029, 2739) | 1704 (1004, 2573) | -5.02 | 207.53 (120.51,314.92) | | 208.47 (120.95,319.31) | 0.45 | 0.02 (0.01 to 0.03) | |
| Australia | 13458 (9007, 18450) | 18994 (12829, 25621) | 41.14 | 294.53 (196.67,404.61) | | 292.73 (196.35,397.1) | -0.61 | 0 (-0.01 to 0.01) | |
| Austria | 4555 (2706, 6956) | 4882 (2925, 7415) | 7.18 | 222.16 (131.38,340.43) | | 223.09 (131.4,343.03) | 0.42 | 0 (-0.01 to 0.02) | |
| Azerbaijan | 3624 (2045, 5552) | 6100 (3535, 9349) | 68.32 | 204.25 (117.68,307.87) | | 206.01 (118.26,318.05) | 0.86 | 0.07 (0.04 to 0.09) | |
| Bahamas | 128 (72, 203) | 202 (119, 310) | 57.81 | 186.15 (105.98,290.19) | | 184.02 (107.27,282.91) | -1.14 | -0.02 (-0.03 to -0.01) | |
| Bahrain | 261 (153, 398) | 779 (464, 1187) | 198.47 | 231.34 (138.79,347.66) | | 231.04 (136.89,353.75) | -0.13 | 0 (-0.01 to 0.01) | |
| Bangladesh | 50092 (28688, 77567) | 104011 (63016, 157048) | 107.64 | 232.47 (137.09,351.54) | | 229.66 (139.77,345.66) | -1.21 | -0.04 (-0.05 to -0.03) | |
| Barbados | 144 (88, 212) | 150 (88, 226) | 4.17 | 211.21 (129.52,311.08) | | 198.31 (115.34,302.81) | -6.11 | -0.06 (-0.1 to -0.02) | |
| Belarus | 5067 (2959, 7689) | 4645 (2804, 6840) | -8.33 | 192.94 (112.29,294.01) | | 194.21 (114.37,291.4) | 0.66 | 0.03 (0.02 to 0.05) | |
| Belgium | 5646 (3430, 8578) | 5918 (3581, 8843) | 4.82 | 221.84 (133.95,338.64) | | 219.6 (130.72,332.28) | -1.01 | -0.02 (-0.03 to -0.01) | |
| Belize | 66 (37, 104) | 206 (119, 321) | 212.12 | 179.11 (105.08,276.94) | | 177.8 (103.95,274.62) | -0.73 | 0 (-0.02 to 0.01) | |
| Benin | 2324 (1360, 3600) | 6886 (4117, 10556) | 196.30 | 233.33 (139.87,355.2) | | 237.22 (144.29,357.29) | 1.67 | 0.04 (0.03 to 0.05) | |
| Bermuda | 36 (21, 54) | 29 (17, 44) | -19.44 | 195.69 (111.99,299.4) | | 196.66 (113.95,303.61) | 0.50 | 0.04 (0.03 to 0.06) | |
| Bhutan | 289 (163, 443) | 489 (293, 748) | 69.20 | 234.34 (136.63,350.63) | | 236.54 (141.83,360.18) | 0.94 | 0.02 (0.01 to 0.03) | |
| Bolivia (Plurinational State of) | 2626 (1510, 4100) | 5825 (3420, 8905) | 121.82 | 186.6 (109.24,286.8) | | 189.15 (111.54,288.09) | 1.37 | 0.04 (0.03 to 0.06) | |
| Bosnia and Herzegovina | 2349 (1373, 3618) | 1637 (981, 2490) | -30.31 | 200.88 (117.89,308.83) | | 205.84 (121.49,317.8) | 2.47 | 0.09 (0.07 to 0.11) | |
| Botswana | 596 (344, 937) | 1484 (874, 2271) | 148.99 | 211.48 (125.29,326.09) | | 215.29 (126.92,329.44) | 1.80 | 0.1 (0.07 to 0.12) | |
| Brazil | 72943 (49140, 101045) | 123006 (83483, 167046) | 68.63 | 198.48 (134.5,272.85) | | 199.13 (134.82,271.58) | 0.33 | 0.02 (0 to 0.04) | |
| Brunei Darussalam | 161 (93, 249) | 325 (195, 489) | 101.86 | 251.95 (149.29,383.8) | | 246.37 (146.66,372.75) | -2.21 | -0.07 (-0.08 to -0.05) | |
| Bulgaria | 4319 (2558, 6505) | 3192 (1944, 4768) | -26.09 | 199.42 (116.92,302.66) | | 199.66 (119,303.61) | 0.12 | 0.01 (0 to 0.02) | |
| Burkina Faso | 4289 (2525, 6719) | 11361 (6588, 17509) | 164.89 | 227.5 (136.74,349.66) | | 231.02 (137.21,349.58) | 1.55 | 0.05 (0.04 to 0.06) | |
| Burundi | 2364 (1374, 3750) | 5698 (3283, 8845) | 141.03 | 206.97 (122.96,323.54) | | 203.31 (119.57,309.58) | -1.77 | -0.03 (-0.05 to -0.01) | |
| Cabo Verde | 172 (99, 265) | 380 (235, 577) | 120.93 | 249.19 (147.88,376.51) | | 253.17 (156.83,382.96) | 1.60 | 0.07 (0.05 to 0.08) | |
| Cambodia | 5252 (3050, 8106) | 10670 (6262, 16529) | 103.16 | 224.51 (132.27,343.12) | | 235.42 (138.75,363.69) | 4.86 | 0.23 (0.19 to 0.28) | |
| Cameroon | 4936 (2860, 7713) | 16569 (9495, 25179) | 235.68 | 232.75 (138.3,356.44) | | 232.04 (135.82,347.21) | -0.31 | 0 (-0.01 to 0.01) | |
| Canada | 19280 (13502, 25634) | 22178 (15748, 29020) | 15.03 | 247.56 (172.6,330.07) | | 248.14 (175.12,326.34) | 0.23 | -0.01 (-0.01 to 0) | |
| Central African Republic | 1129 (648, 1760) | 2413 (1384, 3788) | 113.73 | 194.84 (114.77,298.34) | | 192.15 (112.17,297.06) | -1.38 | -0.03 (-0.04 to -0.01) | |
| Chad | 2730 (1588, 4281) | 7701 (4408, 11916) | 182.09 | 229.19 (136.52,352.46) | | 230.63 (135.85,348.29) | 0.63 | 0.05 (0.04 to 0.07) | |
| Chile | 8627 (5138, 12833) | 12482 (7448, 18691) | 44.69 | 247.91 (148.95,366.14) | | 250.97 (148.71,377.97) | 1.23 | 0.05 (0.03 to 0.07) | |
| China | 866426 (604065, 1158773) | 1037160 (732216, 1362681) | 19.71 | 282.8 (198.39,375.5) | | 297.69 (208.54,394.88) | 5.27 | 0.11 (0.08 to 0.13) | |
| Colombia | 16171 (9244, 25133) | 26768 (15972, 40884) | 65.53 | 200.11 (116.35,306.38) | | 200.73 (119.7,306.78) | 0.31 | 0.02 (0.01 to 0.03) | |
| Comoros | 203 (117, 320) | 415 (244, 647) | 104.43 | 220.98 (130.88,339.9) | | 221.14 (131.16,342.71) | 0.07 | 0.03 (0.02 to 0.05) | |
| Congo | 1027 (585, 1637) | 2881 (1670, 4406) | 180.53 | 209.57 (123.1,326.38) | | 209.68 (122.3,319.13) | 0.05 | 0.05 (0.02 to 0.07) | |
| Cook Islands | 11 (7, 17) | 12 (7, 18) | 9.09 | 265.46 (160.03,403.66) | | 270.66 (157.82,417.33) | 1.96 | 0.07 (0.05 to 0.1) | |
| Costa Rica | 1497 (866, 2325) | 2746 (1595, 4181) | 83.43 | 203.5 (119.26,312.62) | | 203.69 (117.69,311.19) | 0.09 | 0.03 (0.02 to 0.04) | |
| Coted'Ivoire | 5586 (3235, 8786) | 14507 (8525, 22388) | 159.70 | 230.93 (138.22,355.36) | | 232.65 (138.92,354.07) | 0.74 | 0.01 (0 to 0.03) | |
| Croatia | 2635 (1572, 4027) | 2058 (1219, 3089) | -21.90 | 208.92 (123.57,321.09) | | 209.86 (122.25,319.27) | 0.45 | 0.04 (0.03 to 0.05) | |
| Cuba | 5443 (3113, 8477) | 4913 (2911, 7422) | -9.74 | 185.59 (107.53,286.03) | | 184.66 (107.72,282.57) | -0.50 | 0.02 (0 to 0.04) | |
| Cyprus | 449 (263, 674) | 914 (552, 1351) | 103.56 | 222.57 (130.12,334.2) | | 222.66 (131.48,334.27) | 0.04 | 0 (-0.02 to 0.01) | |
| Czechia | 5633 (3314, 8472) | 5465 (3323, 8107) | -2.98 | 210.21 (122.16,319.63) | | 211.81 (125.33,321.37) | 0.76 | 0.01 (-0.01 to 0.02) | |
| Democratic People's Republic of Korea | 13271 (7777, 20834) | 16133 (9715, 24497) | 21.57 | 242.64 (143.64,376.92) | | 235.64 (140.64,360.05) | -2.88 | -0.13 (-0.14 to -0.11) | |
| Democratic Republic of the Congo | 15555 (8904, 24005) | 38578 (22696, 59403) | 148.01 | 204.13 (120.09,309.24) | | 201.34 (120.6,304.77) | -1.37 | -0.02 (-0.05 to 0.01) | |
| Denmark | 2922 (1842, 4234) | 3938 (2766, 5166) | 34.77 | 211.75 (132.57,308.87) | | 294.2 (206.39,387.01) | 38.94 | 1.53 (1.25 to 1.8) | |
| Djibouti | 189 (108, 298) | 717 (425, 1101) | 279.37 | 221.03 (130.74,341.03) | | 219.95 (130.31,338.53) | -0.49 | 0.02 (0 to 0.04) | |
| Dominica | 28 (16, 44) | 30 (17, 45) | 7.14 | 181.65 (105.55,280.23) | | 179.73 (103.55,274.98) | -1.06 | 0 (-0.02 to 0.01) | |
| Dominican Republic | 3060 (1720, 4765) | 5230 (3046, 8020) | 70.92 | 179.66 (104.08,274.22) | | 181.57 (106.04,277.82) | 1.06 | 0.06 (0.05 to 0.07) | |
| Ecuador | 4520 (2591, 7024) | 9207 (5328, 14331) | 103.69 | 196.53 (115.03,300.74) | | 196.21 (113.92,304.66) | -0.16 | 0.01 (0 to 0.02) | |
| Egypt | 26670 (15655, 41196) | 56165 (33680, 85776) | 110.59 | 218.19 (129.9,333.18) | | 220.84 (133.07,335.81) | 1.21 | 0.01 (0 to 0.03) | |
| El Salvador | 2266 (1300, 3565) | 3454 (2007, 5340) | 52.43 | 193.78 (113.95,299.11) | | 195.36 (113.71,301.1) | 0.82 | 0.05 (0.03 to 0.06) | |
| Equatorial Guinea | 181 (104, 278) | 747 (428, 1156) | 312.71 | 203.06 (119.59,305.67) | | 221.66 (129.58,337.97) | 9.16 | 0.41 (0.34 to 0.49) | |
| Eritrea | 1474 (826, 2303) | 3321 (1929, 5210) | 125.31 | 209.38 (120.24,320.8) | | 213.8 (125.82,331.17) | 2.11 | 0.08 (0.05 to 0.1) | |
| Estonia | 780 (462, 1184) | 613 (363, 917) | -21.41 | 194.58 (114.13,297.18) | | 198.92 (115.21,302.03) | 2.23 | 0.06 (0.05 to 0.07) | |
| Eswatini | 359 (205, 558) | 619 (361, 967) | 72.42 | 211.61 (124.27,322.17) | | 206.18 (122.17,317.79) | -2.57 | -0.05 (-0.08 to -0.02) | |
| Ethiopia | 21772 (14516, 30672) | 54210 (35816, 75568) | 148.99 | 215.73 (145.66,300.26) | | 218.64 (146.15,301.49) | 1.35 | 0.11 (0.09 to 0.13) | |
| Fiji | 463 (270, 718) | 576 (344, 876) | 24.41 | 248.31 (146.36,381.57) | | 251.74 (150.21,382.77) | 1.38 | 0.06 (0.04 to 0.07) | |
| Finland | 3262 (2134, 4640) | 2648 (1627, 3924) | -18.82 | 236.07 (152.75,338.99) | | 215.35 (130.71,321.39) | -8.78 | -0.33 (-0.41 to -0.25) | |
| France | 32409 (19087, 49127) | 32964 (19862, 49379) | 1.71 | 217.59 (127.63,330.93) | | 216.77 (128.68,328.75) | -0.38 | 0 (-0.01 to 0.01) | |
| Gabon | 429 (252, 671) | 1002 (576, 1534) | 133.57 | 221.33 (133.5,338.72) | | 219.47 (128.37,331.76) | -0.84 | 0 (-0.02 to 0.01) | |
| Gambia | 472 (276, 739) | 1270 (749, 1980) | 169.07 | 235.71 (141.52,360.33) | | 230.64 (139.62,352.03) | -2.15 | -0.04 (-0.06 to -0.03) | |
| Georgia | 9409 (6022, 13561) | 10541 (6928, 14847) | 12.03 | 337.68 (216.11,486.6) | | 621.48 (401.92,885.24) | 84.04 | 2.08 (2.03 to 2.13) | |
| Germany | 44587 (26738, 67469) | 40581 (24036, 60103) | -8.98 | 215.99 (128.97,328.2) | | 215.76 (125.83,323.86) | -0.11 | -0.01 (-0.04 to 0.02) | |
| Ghana | 7489 (4305, 11692) | 20768 (12229, 32046) | 177.31 | 234.28 (138.45,358.42) | | 237.98 (141.47,362.95) | 1.58 | 0.08 (0.07 to 0.09) | |
| Greece | 5726 (3408, 8718) | 5428 (3298, 8061) | -5.20 | 221.47 (131.19,338.6) | | 220.14 (130.61,333.92) | -0.60 | -0.02 (-0.04 to -0.01) | |
| Greenland | 43 (27, 65) | 39 (24, 57) | -9.30 | 289.99 (181.39,430.33) | | 293.9 (183.57,437) | 1.35 | 0.11 (0.08 to 0.15) | |
| Grenada | 32 (18, 50) | 46 (27, 71) | 43.75 | 177.93 (103.3,275.27) | | 180.12 (103.62,277.3) | 1.23 | 0.05 (0.03 to 0.06) | |
| Guam | 95 (56, 146) | 100 (60, 153) | 5.26 | 276.3 (165.33,421.62) | | 275.22 (164.96,420.68) | -0.39 | 0.03 (0.01 to 0.05) | |
| Guatemala | 3043 (1721, 4809) | 7804 (4456, 12040) | 156.46 | 185.59 (107.37,287.68) | | 187.73 (108.68,286.28) | 1.15 | 0.04 (0.02 to 0.05) | |
| Guinea | 2984 (1739, 4587) | 6982 (4079, 10736) | 133.98 | 232.72 (137.57,353.73) | | 230.94 (137.43,349.32) | -0.76 | -0.03 (-0.04 to -0.01) | |
| Guinea-Bissau | 475 (277, 736) | 1105 (647, 1711) | 132.63 | 227.49 (135.94,346.15) | | 227.65 (135.45,347.87) | 0.07 | -0.01 (-0.03 to 0) | |
| Guyana | 317 (177, 508) | 344 (199, 538) | 8.52 | 170.43 (97.68,267.19) | | 172.52 (100.27,268.04) | 1.23 | 0.04 (0.02 to 0.05) | |
| Haiti | 2294 (1302, 3589) | 5496 (3227, 8662) | 139.58 | 160.94 (93.02,248.28) | | 157.98 (93.26,247.85) | -1.84 | -0.04 (-0.05 to -0.03) | |
| Honduras | 1761 (993, 2780) | 5042 (2916, 7902) | 186.31 | 188.21 (108.68,290.7) | | 186.75 (109.49,289.59) | -0.78 | -0.02 (-0.03 to -0.01) | |
| Hungary | 5423 (3208, 8093) | 4913 (2944, 7316) | -9.40 | 203.08 (118.31,306.41) | | 207.36 (121.32,315.7) | 2.11 | 0.05 (0.04 to 0.06) | |
| Iceland | 145 (85, 220) | 189 (112, 285) | 30.34 | 223.84 (132.37,339.14) | | 224.16 (131.22,339.57) | 0.14 | -0.01 (-0.01 to 0) | |
| India | 431303 (295391, 588290) | 884468 (604782, 1209754) | 105.07 | 226.98 (156.18,307.11) | | 236.61 (162.04,322.83) | 4.24 | 0.17 (0.14 to 0.21) | |
| Indonesia | 109737 (74376, 151422) | 193156 (132503, 265695) | 76.02 | 244.33 (166.72,334.37) | | 250.23 (171.35,345.13) | 2.41 | 0.16 (0.12 to 0.21) | |
| Iran (Islamic Republic of) | 25316 (17180, 34994) | 57427 (39485, 76973) | 126.84 | 224.76 (153.97,306.72) | | 225.89 (154.37,305.8) | 0.50 | 0.01 (-0.01 to 0.02) | |
| Iraq | 7838 (4696, 12259) | 22006 (13165, 33756) | 180.76 | 218.84 (134.47,334.09) | | 217.51 (131.38,331.18) | -0.61 | 0.01 (0 to 0.03) | |
| Ireland | 2401 (1540, 3429) | 3508 (2125, 5178) | 46.11 | 278.13 (178.66,396.61) | | 277.58 (165.06,417.55) | -0.20 | 0.13 (0.05 to 0.22) | |
| Israel | 2515 (1498, 3846) | 4763 (2787, 7208) | 89.38 | 211.62 (126.78,322.58) | | 209.72 (122.03,318.47) | -0.90 | -0.01 (-0.03 to 0) | |
| Italy | 28265 (19367, 38709) | 26758 (18487, 36214) | -5.33 | 193.23 (132.23,265.18) | | 194.84 (133.18,267.67) | 0.83 | 0.02 (0.02 to 0.03) | |
| Jamaica | 983 (538, 1567) | 1414 (814, 2159) | 43.85 | 181.84 (102.53,284) | | 180.92 (104.24,275.9) | -0.51 | -0.01 (-0.02 to 0) | |
| Japan | 82318 (56432, 111309) | 69539 (48070, 93335) | -15.52 | 244.57 (166.67,333.5) | | 250.49 (171.18,341.03) | 2.42 | 0.21 (0.14 to 0.28) | |
| Jordan | 1579 (895, 2479) | 6602 (3911, 10236) | 318.11 | 222.86 (130.97,339.98) | | 223.48 (133.62,343.88) | 0.28 | -0.03 (-0.04 to -0.02) | |
| Kazakhstan | 8244 (4855, 12616) | 10335 (6127, 15535) | 25.36 | 202.54 (119.92,308.49) | | 204.61 (119.87,310.71) | 1.02 | 0.08 (0.06 to 0.1) | |
| Kenya | 9525 (6425, 13259) | 26283 (17831, 36022) | 175.94 | 217.34 (148.46,297.64) | | 217.81 (148.9,295.21) | 0.22 | 0.04 (0.02 to 0.05) | |
| Kiribati | 41 (23, 64) | 71 (42, 110) | 73.17 | 229 (130.91,353.2) | | 228.04 (134.65,348.1) | -0.42 | -0.01 (-0.03 to 0.01) | |
| Kuwait | 965 (570, 1491) | 3982 (2460, 5871) | 312.64 | 237.88 (142.54,362.84) | | 237.93 (143.31,357.09) | 0.02 | -0.03 (-0.04 to -0.02) | |
| Kyrgyzstan | 1941 (1125, 3024) | 3455 (2045, 5329) | 78.00 | 198.8 (117.92,304.77) | | 198.45 (117.41,305.59) | -0.18 | -0.01 (-0.02 to 0.01) | |
| Lao People's Democratic Republic | 2014 (1183, 3180) | 4662 (2737, 7175) | 131.48 | 226.33 (135.41,351.7) | | 238.55 (141.23,364.71) | 5.40 | 0.27 (0.23 to 0.31) | |
| Latvia | 1313 (785, 1987) | 848 (510, 1274) | -35.42 | 193.35 (114.58,294.52) | | 195.69 (115.25,299.62) | 1.21 | 0.06 (0.04 to 0.07) | |
| Lebanon | 1615 (943, 2459) | 3599 (2161, 5454) | 122.85 | 225.06 (133.28,339.85) | | 225.39 (133.96,344.96) | 0.15 | 0.01 (0 to 0.02) | |
| Lesotho | 693 (393, 1096) | 922 (527, 1448) | 33.04 | 197.55 (113.57,308.86) | | 195.61 (113.72,302.37) | -0.98 | 0 (-0.02 to 0.01) | |
| Liberia | 1127 (653, 1772) | 2859 (1656, 4399) | 153.68 | 224.75 (134.59,345.04) | | 221.57 (130.57,337.2) | -1.41 | 0.01 (-0.01 to 0.03) | |
| Libya | 1758 (1003, 2735) | 4547 (2739, 6886) | 158.65 | 228.65 (134.86,346.98) | | 220.99 (132.12,336.67) | -3.35 | -0.13 (-0.16 to -0.11) | |
| Lithuania | 1839 (1098, 2825) | 1223 (738, 1861) | -33.50 | 194.02 (115.4,298.81) | | 195.84 (115.94,302.35) | 0.94 | 0.05 (0.04 to 0.06) | |
| Luxembourg | 232 (138, 354) | 392 (235, 585) | 68.97 | 223.5 (132.1,342.4) | | 225.18 (132.58,340.44) | 0.75 | 0.04 (0.03 to 0.06) | |
| Madagascar | 5070 (2849, 7952) | 13855 (7964, 21725) | 173.27 | 213.89 (123.55,328.91) | | 212.2 (124.98,326.22) | -0.79 | 0 (-0.02 to 0.01) | |
| Malawi | 3937 (2244, 6185) | 8801 (5006, 13980) | 123.55 | 198.85 (116.6,305.29) | | 201.93 (118.6,313.86) | 1.55 | 0.09 (0.07 to 0.11) | |
| Malaysia | 11040 (6466, 17196) | 22669 (13608, 34915) | 105.34 | 257.9 (152.93,396.8) | | 265.78 (159.68,408.75) | 3.06 | 0.16 (0.12 to 0.2) | |
| Maldives | 103 (59, 161) | 316 (192, 480) | 206.80 | 246.89 (146.92,376.55) | | 256.44 (154.17,392.33) | 3.87 | 0.21 (0.17 to 0.24) | |
| Mali | 3989 (2296, 6271) | 10969 (6197, 17276) | 174.98 | 227.68 (133.61,351.58) | | 231.38 (134.79,356.75) | 1.63 | 0.06 (0.04 to 0.07) | |
| Malta | 225 (138, 338) | 237 (144, 352) | 5.33 | 221.84 (133.75,337.19) | | 223.03 (133.08,337.5) | 0.54 | 0.01 (0 to 0.02) | |
| Marshall Islands | 21 (12, 32) | 34 (20, 51) | 61.90 | 234.02 (138.31,359.13) | | 233.1 (137.97,353.89) | -0.39 | -0.01 (-0.03 to 0.01) | |
| Mauritania | 1020 (600, 1600) | 2351 (1359, 3659) | 130.49 | 241.27 (145.27,371.44) | | 243.16 (143.61,371.71) | 0.78 | 0.02 (0.02 to 0.03) | |
| Mauritius | 751 (441, 1157) | 852 (519, 1274) | 13.45 | 253.76 (150.2,387.86) | | 260.07 (157.24,391.33) | 2.49 | 0.15 (0.11 to 0.19) | |
| Mexico | 40314 (27220, 55777) | 72933 (50010, 99003) | 80.91 | 205.88 (140.48,281.23) | | 205.16 (140.53,278.94) | -0.35 | -0.01 (-0.02 to 0) | |
| Micronesia (Federated States of) | 49 (28, 76) | 58 (34, 91) | 18.37 | 234.45 (136.66,357.14) | | 235.01 (137.7,362.32) | 0.24 | 0.01 (-0.01 to 0.03) | |
| Monaco | 18 (11, 28) | 19 (11, 28) | 5.56 | 232.29 (139.54,352.76) | | 232.74 (137.93,357.69) | 0.19 | 0 (-0.02 to 0.01) | |
| Mongolia | 878 (500, 1373) | 1780 (1063, 2746) | 102.73 | 193.67 (114.24,296.21) | | 198.84 (117.43,308.94) | 2.67 | 0.11 (0.09 to 0.12) | |
| Montenegro | 322 (188, 497) | 314 (190, 471) | -2.48 | 205.69 (120.09,317.3) | | 204.46 (121.86,309.65) | -0.60 | 0.01 (0 to 0.02) | |
| Morocco | 12461 (7214, 19380) | 21139 (12518, 32066) | 69.64 | 215.21 (127.12,329.16) | | 215.08 (127.06,326.96) | -0.06 | -0.02 (-0.03 to -0.01) | |
| Mozambique | 5540 (3171, 8547) | 12964 (7310, 20384) | 134.01 | 193.78 (112.99,295.05) | | 195.4 (113.18,300.76) | 0.84 | 0.1 (0.07 to 0.13) | |
| Myanmar | 21748 (12658, 34182) | 35554 (21392, 54491) | 63.48 | 223.89 (132.37,346.28) | | 236.93 (142.76,362.93) | 5.82 | 0.3 (0.25 to 0.35) | |
| Namibia | 635 (364, 986) | 1368 (816, 2104) | 115.43 | 214.68 (126.26,326.08) | | 215.74 (129.89,328.85) | 0.49 | 0.07 (0.05 to 0.09) | |
| Nauru | 6 (3, 9) | 7 (4, 10) | 16.67 | 253.93 (149.97,386.13) | | 246.11 (145.43,372.53) | -3.08 | -0.08 (-0.15 to 0) | |
| Nepal | 9500 (5646, 14498) | 19804 (11720, 29938) | 108.46 | 226.67 (137.42,341.26) | | 226.41 (135.08,339.84) | -0.11 | -0.03 (-0.04 to -0.01) | |
| Netherlands | 12972 (8579, 18406) | 12025 (7835, 17008) | -7.30 | 315.6 (207.83,449.29) | | 307.59 (198.64,438.82) | -2.54 | -0.1 (-0.12 to -0.07) | |
| New Zealand | 2693 (1759, 3805) | 3792 (2514, 5331) | 40.81 | 295.94 (193.05,418.15) | | 300.57 (198.33,424.7) | 1.56 | 0.02 (0 to 0.04) | |
| Nicaragua | 1550 (896, 2421) | 3513 (2072, 5437) | 126.65 | 196.29 (117.19,300.52) | | 195.46 (115.63,301.45) | -0.42 | 0 (-0.01 to 0.01) | |
| Niger | 3536 (2059, 5526) | 10282 (5905, 16114) | 190.78 | 229.98 (137.42,353) | | 230.42 (137.5,351.79) | 0.19 | 0 (-0.01 to 0.02) | |
| Nigeria | 45709 (30963, 63027) | 134169 (90841, 184316) | 193.53 | 262.03 (179.62,355.12) | | 265.07 (181.15,359.58) | 1.16 | 0.07 (0.06 to 0.08) | |
| Niue | 1 (1, 2) | 1 (1, 2) | 0.00 | 254.13 (151.61,386.79) | | 257.4 (155.11,391.9) | 1.29 | 0.06 (0.05 to 0.08) | |
| North Macedonia | 1047 (615, 1609) | 1189 (718, 1808) | 13.56 | 204.65 (120.16,314.52) | | 203.64 (121.01,313.36) | -0.49 | 0.01 (-0.01 to 0.02) | |
| Northern Mariana Islands | 38 (22, 58) | 32 (19, 47) | -15.79 | 278.55 (167.93,424.01) | | 271.88 (162.73,408.26) | -2.39 | -0.1 (-0.13 to -0.08) | |
| Norway | 2517 (1727, 3408) | 2998 (2046, 4107) | 19.11 | 230.67 (157.81,313.2) | | 229.73 (155.8,316.83) | -0.41 | 0.02 (0 to 0.03) | |
| Oman | 716 (416, 1108) | 2480 (1464, 3789) | 246.37 | 227.47 (134.6,347.07) | | 229.1 (133.96,352.46) | 0.72 | 0.01 (-0.01 to 0.02) | |
| Pakistan | 49934 (33091, 70437) | 135136 (89475, 189846) | 170.63 | 236.74 (158.63,330.25) | | 233.01 (155.09,325.3) | -1.58 | -0.06 (-0.07 to -0.05) | |
| Palau | 10 (6, 15) | 10 (6, 15) | 0.00 | 253.78 (149.65,387.58) | | 255.96 (153.76,387.97) | 0.86 | 0.03 (0.01 to 0.05) | |
| Palestine | 851 (489, 1330) | 2697 (1578, 4142) | 216.92 | 222.47 (132.29,338.91) | | 221.03 (131.66,335.31) | -0.65 | -0.04 (-0.05 to -0.02) | |
| Panama | 1135 (664, 1761) | 2170 (1295, 3361) | 91.19 | 200.05 (118.9,305.97) | | 203.19 (121.32,314.28) | 1.57 | 0.05 (0.03 to 0.06) | |
| Papua New Guinea | 2074 (1221, 3177) | 6068 (3626, 9285) | 192.57 | 233.56 (140.41,353.1) | | 237.54 (142.94,361.07) | 1.70 | 0.05 (0.03 to 0.07) | |
| Paraguay | 1703 (973, 2656) | 3678 (2161, 5548) | 115.97 | 196.4 (114.7,301.3) | | 196.67 (116.11,295.69) | 0.14 | 0 (-0.02 to 0.01) | |
| Peru | 9714 (5539, 15233) | 19480 (11383, 29691) | 100.54 | 196.37 (114.31,302.44) | | 198.27 (115.65,302.63) | 0.97 | 0.05 (0.04 to 0.07) | |
| Philippines | 35571 (24122, 48981) | 72071 (49326, 98497) | 102.61 | 248.52 (169.86,338.36) | | 251.74 (172.74,342.56) | 1.30 | 0.12 (0.08 to 0.16) | |
| Poland | 20512 (13934, 28041) | 21075 (14427, 28200) | 2.74 | 207.12 (140.21,284.6) | | 211.55 (143.64,286.4) | 2.14 | 0.08 (0.06 to 0.09) | |
| Portugal | 5519 (3307, 8358) | 5761 (3465, 8436) | 4.38 | 216.76 (129.64,328.91) | | 218.82 (128.28,327.71) | 0.95 | 0.01 (0 to 0.03) | |
| Puerto Rico | 1825 (1049, 2808) | 1507 (893, 2298) | -17.42 | 191.48 (110.13,294.52) | | 191.2 (112.02,293.7) | -0.15 | 0.01 (0 to 0.03) | |
| Qatar | 194 (118, 293) | 1517 (913, 2301) | 681.96 | 238.24 (144.92,361.21) | | 240.11 (142.95,369.86) | 0.78 | 0.02 (0.01 to 0.03) | |
| Republic of Korea | 29857 (17613, 46021) | 32204 (19598, 48415) | 7.86 | 245.1 (146.28,374.21) | | 246.72 (147.46,376.75) | 0.66 | 0.01 (0 to 0.03) | |
| Republic of Moldova | 2208 (1299, 3360) | 1895 (1134, 2832) | -14.18 | 189.6 (110.85,290.1) | | 189.96 (111.07,289.68) | 0.19 | 0 (-0.01 to 0.02) | |
| Romania | 11351 (6509, 17281) | 9098 (5418, 13752) | -19.85 | 201.86 (115.84,307.96) | | 204.91 (118.91,316.22) | 1.51 | 0.08 (0.07 to 0.1) | |
| Russian Federation | 71086 (49043, 96323) | 73929 (50956, 98890) | 4.00 | 182.37 (125.57,248.11) | | 194.48 (132.69,263.78) | 6.64 | 0.31 (0.25 to 0.38) | |
| Rwanda | 3031 (1758, 4745) | 6919 (4067, 10717) | 128.27 | 210.59 (125.74,323.23) | | 211.32 (126.04,323.39) | 0.35 | 0.09 (0.05 to 0.12) | |
| Saint Kitts and Nevis | 17 (10, 26) | 30 (18, 46) | 76.47 | 182.04 (109.54,279.8) | | 185.45 (107.5,283.89) | 1.87 | 0.07 (0.06 to 0.08) | |
| Saint Lucia | 56 (31, 86) | 88 (50, 134) | 57.14 | 181.39 (104.38,276.67) | | 181.76 (103.45,281.65) | 0.20 | 0 (-0.01 to 0.02) | |
| Saint Vincent and the Grenadines | 41 (23, 66) | 51 (29, 78) | 24.39 | 176.92 (102.18,274.53) | | 178.26 (101.85,275.02) | 0.76 | 0.04 (0.02 to 0.05) | |
| Samoa | 79 (46, 124) | 113 (67, 173) | 43.04 | 244.15 (144.9,375.19) | | 246.62 (146.87,374.75) | 1.01 | 0.05 (0.04 to 0.07) | |
| San Marino | 14 (8, 22) | 18 (11, 27) | 28.57 | 227.23 (133.08,343.48) | | 224.21 (130.37,343.53) | -1.33 | -0.03 (-0.04 to -0.02) | |
| Sao Tome and Principe | 54 (31, 84) | 129 (77, 196) | 138.89 | 245.64 (145.81,371.01) | | 247.25 (149.24,372.56) | 0.66 | 0.02 (0.01 to 0.03) | |
| Saudi Arabia | 6607 (3824, 10187) | 24898 (14950, 37695) | 276.84 | 226.72 (134.24,342.73) | | 226.97 (134.69,346.74) | 0.11 | -0.02 (-0.03 to -0.01) | |
| Senegal | 3600 (2112, 5590) | 8565 (5038, 13211) | 137.92 | 238.48 (143.76,363.24) | | 239.44 (143.4,363.99) | 0.40 | 0.04 (0.03 to 0.05) | |
| Serbia | 4924 (2922, 7540) | 4493 (2689, 6923) | -8.75 | 204.86 (120.92,315.02) | | 205.28 (121.08,320.94) | 0.21 | 0.03 (0.01 to 0.04) | |
| Seychelles | 44 (26, 68) | 66 (40, 100) | 50.00 | 259.73 (156.24,400.1) | | 263.44 (159.4,401.17) | 1.43 | 0.1 (0.07 to 0.14) | |
| Sierra Leone | 2139 (1258, 3289) | 4702 (2700, 7379) | 119.82 | 231.53 (139.4,349.69) | | 229.52 (135.12,353.73) | -0.87 | -0.03 (-0.05 to -0.01) | |
| Singapore | 2393 (1428, 3703) | 4417 (2728, 6463) | 84.58 | 251.9 (150.97,388.59) | | 256.38 (154.55,384.61) | 1.78 | 0.07 (0.06 to 0.08) | |
| Slovakia | 2825 (1648, 4287) | 2952 (1775, 4397) | 4.50 | 206.76 (119.94,315.34) | | 208.32 (121.78,315.55) | 0.75 | 0.05 (0.03 to 0.06) | |
| Slovenia | 1075 (627, 1643) | 1001 (609, 1518) | -6.88 | 207.93 (120.6,319.51) | | 212.52 (126.43,329.52) | 2.21 | 0.04 (0.03 to 0.05) | |
| Solomon Islands | 149 (85, 236) | 376 (221, 577) | 152.35 | 229.23 (135.14,353.13) | | 229.6 (136.1,349.73) | 0.16 | 0.02 (0 to 0.04) | |
| Somalia | 3091 (1795, 4782) | 8331 (4694, 12877) | 169.52 | 197.6 (116.4,302.71) | | 195.4 (112.68,297.36) | -1.11 | -0.04 (-0.06 to -0.03) | |
| South Africa | 19275 (12961, 26684) | 34091 (23206, 46374) | 76.87 | 218.21 (147.75,298.68) | | 213.72 (145.18,291.2) | -2.06 | -0.03 (-0.05 to -0.01) | |
| South Sudan | 2439 (1388, 3758) | 4372 (2499, 6752) | 79.25 | 217.59 (127.22,328.62) | | 211.25 (122.69,321.97) | -2.91 | -0.04 (-0.07 to -0.01) | |
| Spain | 19096 (13003, 25887) | 22605 (15538, 30477) | 18.38 | 199.49 (135.98,270.26) | | 198.67 (133.97,271.41) | -0.41 | -0.01 (-0.02 to -0.01) | |
| Sri Lanka | 11173 (6483, 17213) | 14928 (8862, 22642) | 33.61 | 250.54 (146.74,383.14) | | 259.7 (153.04,395.83) | 3.66 | 0.2 (0.15 to 0.24) | |
| Sudan | 8723 (5086, 13545) | 22160 (12833, 34785) | 154.04 | 206.39 (123.33,313.9) | | 208.59 (122.76,323.27) | 1.07 | 0.02 (0.01 to 0.04) | |
| Suriname | 141 (85, 208) | 229 (144, 333) | 62.41 | 155.92 (95.73,228.29) | | 155.09 (97.57,225.89) | -0.53 | 0.01 (0 to 0.02) | |
| Sweden | 4468 (2924, 6355) | 4793 (3095, 6815) | 7.27 | 200.28 (129.52,288.03) | | 198.67 (126.76,285.14) | -0.80 | -0.04 (-0.06 to -0.03) | |
| Switzerland | 4248 (2498, 6350) | 4921 (2981, 7311) | 15.84 | 224.93 (131.06,338.86) | | 224.35 (133.46,338.73) | -0.26 | 0.01 (-0.01 to 0.02) | |
| Syrian Arab Republic | 5193 (2968, 8123) | 7726 (4553, 11795) | 48.78 | 217.7 (128.66,331.77) | | 215.31 (127.94,328.01) | -1.10 | -0.04 (-0.06 to -0.02) | |
| Taiwan (Province of China) | 13527 (8158, 20662) | 16480 (10064, 24435) | 21.83 | 246.38 (149.52,374.44) | | 265.04 (158.72,399.68) | 7.57 | 0.2 (0.16 to 0.23) | |
| Tajikistan | 2111 (1176, 3345) | 4898 (2831, 7622) | 132.02 | 198.5 (115.13,307.07) | | 194.87 (113.34,301.39) | -1.83 | -0.04 (-0.06 to -0.02) | |
| Thailand | 38592 (22629, 59986) | 44890 (26646, 68592) | 16.32 | 249.82 (148.32,384.64) | | 257.68 (150.68,399.11) | 3.15 | 0.17 (0.13 to 0.21) | |
| Timor-Leste | 378 (214, 587) | 679 (388, 1051) | 79.63 | 212.06 (122.53,325.74) | | 217.96 (127.42,331.37) | 2.78 | 0.23 (0.19 to 0.28) | |
| Togo | 1754 (1015, 2765) | 4756 (2826, 7264) | 171.15 | 230.92 (136.76,356.07) | | 230.69 (138.22,349.49) | -0.10 | 0 (-0.01 to 0.01) | |
| Tokelau | 1 (0, 1) | 1 (0, 1) | 0.00 | 241.48 (144.9,367.84) | | 247.47 (147.46,378.85) | 2.48 | 0.09 (0.07 to 0.11) | |
| Tonga | 50 (29, 77) | 61 (36, 92) | 22.00 | 250.97 (150.27,382.44) | | 252.01 (150.29,379.74) | 0.41 | 0.02 (0 to 0.04) | |
| Trinidad and Tobago | 449 (277, 655) | 701 (416, 1041) | 56.12 | 150.7 (93.88,218.55) | | 192.43 (112.73,289.24) | 27.69 | 0.59 (0.37 to 0.82) | |
| Tunisia | 4233 (2434, 6516) | 7329 (4471, 10968) | 73.14 | 225.2 (132.68,340.72) | | 224.03 (134.81,338.69) | -0.52 | -0.03 (-0.04 to -0.02) | |
| Turkey | 29333 (18610, 43538) | 50067 (32046, 72647) | 70.68 | 221.66 (142.69,324.52) | | 222.72 (141.48,324.99) | 0.48 | 0 (-0.01 to 0.02) | |
| Turkmenistan | 1608 (914, 2515) | 2555 (1509, 3944) | 58.89 | 199.57 (116.24,306.69) | | 203.87 (120.65,314.14) | 2.15 | 0.08 (0.05 to 0.1) | |
| Tuvalu | 6 (3, 9) | 7 (4, 10) | 16.67 | 232.69 (138.74,355.76) | | 239.76 (141.31,360.61) | 3.04 | 0.1 (0.09 to 0.12) | |
| Uganda | 6531 (3719, 10188) | 18783 (10713, 29953) | 187.60 | 200.08 (117.99,304.54) | | 207.27 (121.91,322.23) | 3.59 | 0.14 (0.12 to 0.16) | |
| Ukraine | 25693 (15970, 37378) | 21961 (14119, 31237) | -14.53 | 193.95 (119.79,283.72) | | 192.02 (120.91,277.91) | -1.00 | -0.02 (-0.03 to -0.01) | |
| United Arab Emirates | 816 (484, 1263) | 4591 (2838, 6784) | 462.62 | 238.82 (143.64,364.91) | | 232.65 (138.88,354.74) | -2.58 | -0.11 (-0.13 to -0.1) | |
| United Kingdom | 29084 (20175, 39159) | 27422 (18879, 36969) | -5.71 | 197.12 (136.46,266.08) | | 165.34 (113.28,224.55) | -16.12 | -0.48 (-0.64 to -0.32) | |
| United Republic of Tanzania | 10557 (6108, 16551) | 28259 (16033, 43929) | 167.68 | 203.92 (121.81,311.74) | | 210.39 (121.96,321.37) | 3.17 | 0.17 (0.15 to 0.19) | |
| United States Virgin Islands | 56 (33, 85) | 35 (21, 53) | -37.50 | 190.16 (113.04,293.31) | | 190.86 (112.55,295.15) | 0.37 | 0.01 (-0.01 to 0.03) | |
| United States of America | 236872 (166111, 317747) | 257774 (180323, 343996) | 8.82 | 336.23 (235.1,452.16) | | 326.19 (227.52,437.25) | -2.99 | -0.08 (-0.11 to -0.05) | |
| Uruguay | 1866 (1109, 2800) | 2138 (1302, 3244) | 14.58 | 248.67 (147.76,373.22) | | 246.76 (149.19,377.07) | -0.77 | -0.02 (-0.04 to 0.01) | |
| Uzbekistan | 8709 (4984, 13805) | 18318 (10978, 27782) | 110.33 | 196.39 (116.26,303.49) | | 197.41 (117.68,300.84) | 0.52 | 0.04 (0.02 to 0.05) | |
| Vanuatu | 77 (45, 118) | 178 (103, 276) | 131.17 | 237.17 (141.29,358.85) | | 237.14 (139.1,364.21) | -0.01 | 0.01 (-0.01 to 0.03) | |
| Venezuela (Bolivarian Republic of) | 9057 (5173, 14040) | 14256 (8524, 21430) | 57.40 | 200.82 (116.68,307.39) | | 198.82 (117.17,301) | -1.00 | -0.03 (-0.04 to -0.01) | |
| Viet Nam | 45852 (27303, 70249) | 79731 (48678, 118545) | 73.89 | 285.76 (174.36,430.96) | | 295.46 (178.72,442.95) | 3.39 | 0.18 (0.15 to 0.21) | |
| Yemen | 5063 (2971, 7783) | 15734 (9335, 24180) | 210.76 | 202.92 (121.71,307.1) | | 200.77 (120.75,305.44) | -1.06 | -0.04 (-0.06 to -0.02) | |
| Zambia | 3150 (1774, 5013) | 9208 (5376, 14344) | 192.32 | 205.45 (119.8,317.75) | | 209.05 (125.17,319.64) | 1.75 | 0.11 (0.09 to 0.14) | |
| Zimbabwe | 4227 (2363, 6679) | 7491 (4310, 11672) | 77.22 | 205.06 (118.42,316.24) | | 198.9 (116.2,305.91) | -3.00 | -0.11 (-0.14 to -0.08) | |

**Table S6： Age-specific Prevalence of Schizophrenia among women of reproductive age in 1990 and 2021, with trends in age patterns from 1990 to 2021.**

| Location | Age(years) | Prevalence cases | | |  | | Prevalence rates | | |  |
| --- | --- | --- | --- | --- | --- | --- | --- | --- | --- | --- |
|  |  | 1990_thousands(95% UI) | 2021_thousands(95% UI) | Percentage change in case(100%) | | 1990_per 100 000(95% UI) | | 2021_per 100 000(95% UI) | Percentage change in ASRs(100%) | EAPC(95% CI) |
| Global | 45 to 49 | 568.88(466.62-675.27) | 1191.08(972.35-1412.18) | 109.37 | | 499.9(410.04-593.38) | | 505.46(412.64-599.29) | 1.11 | 0.03 (0.02 to 0.05) |
| Global | 40 to 44 | 735.51(601.29-880.6) | 1305.08(1061.78-1571.98) | 77.44 | | 524.52(428.81-627.99) | | 526.05(427.98-633.63) | 0.29 | 0.02 (0.01 to 0.03) |
| Global | 35 to 39 | 915.37(728.83-1121.44) | 1477.3(1164.82-1819.04) | 61.39 | | 527.73(420.19-646.54) | | 531.78(419.3-654.8) | 0.77 | 0.02 (0.01 to 0.03) |
| Global | 30 to 34 | 940.46(730.03-1182.03) | 1497.28(1158.81-1891.21) | 59.21 | | 494.7(384.01-621.77) | | 500.88(387.65-632.66) | 1.25 | 0.02 (0 to 0.04) |
| Global | 25 to 29 | 905.97(660.96-1185.5) | 1182.35(846.16-1571.69) | 30.51 | | 411.62(300.3-538.62) | | 406.32(290.79-540.12) | -1.29 | -0.03 (-0.06 to -0.01) |
| Global | 20 to 24 | 599.39(406.26-851.58) | 689.01(445.94-1016.03) | 14.95 | | 245.52(166.41-348.82) | | 234.56(151.81-345.88) | -4.46 | -0.1 (-0.13 to -0.07) |
| Global | 15 to 19 | 174.53(113.82-256.86) | 199.89(126.02-298.3) | 14.53 | | 68.3(44.54-100.52) | | 65.83(41.5-98.24) | -3.62 | -0.11 (-0.14 to -0.08) |
| High SDI | 45 to 49 | 145.82(120.1-173.08) | 206.54(169.32-245.45) | 41.65 | | 577.11(475.34-685.03) | | 575.23(471.57-683.59) | -0.33 | 0.02 (-0.01 to 0.04) |
| High SDI | 40 to 44 | 184.35(151.4-221.15) | 216.48(176.81-260.29) | 17.43 | | 590.42(484.9-708.28) | | 589.67(481.61-709.02) | -0.13 | 0 (-0.02 to 0.02) |
| High SDI | 35 to 39 | 193.91(155.17-237.51) | 219.86(174.96-270.8) | 13.38 | | 579.89(464.05-710.27) | | 579.61(461.25-713.9) | -0.05 | -0.01 (-0.03 to 0.01) |
| High SDI | 30 to 34 | 190.79(150.11-239.11) | 201.03(156.64-251.85) | 5.37 | | 537.66(423.03-673.84) | | 537.03(418.46-672.8) | -0.12 | 0.01 (-0.01 to 0.04) |
| High SDI | 25 to 29 | 157.16(115.19-204.26) | 152.61(113.33-198.31) | -2.89 | | 438.42(321.35-569.83) | | 441.58(327.92-573.82) | 0.72 | 0.07 (0.04 to 0.09) |
| High SDI | 20 to 24 | 84.49(56.7-121.44) | 80.42(54.34-115.61) | -4.82 | | 251.63(168.85-361.65) | | 255.1(172.39-366.73) | 1.38 | 0.08 (0.03 to 0.12) |
| High SDI | 15 to 19 | 19.67(12.31-29.64) | 18.46(11.67-27.7) | -6.13 | | 61.71(38.64-93) | | 63.46(40.13-95.21) | 2.83 | 0.1 (0.06 to 0.14) |
| High-middle SDI | 45 to 49 | 118.76(99.36-139.41) | 252.47(213.54-292.75) | 112.59 | | 484.38(405.28-568.64) | | 523.49(442.77-607.01) | 8.07 | 0.24 (0.2 to 0.28) |
| High-middle SDI | 40 to 44 | 156.82(131.96-183.76) | 246.91(208.63-289.15) | 57.45 | | 510.08(429.23-597.72) | | 542.43(458.34-635.22) | 6.34 | 0.22 (0.18 to 0.25) |
| High-middle SDI | 35 to 39 | 205.31(167.09-247.4) | 275.74(225.82-331.5) | 34.31 | | 519.81(423.05-626.37) | | 556.48(455.74-669.01) | 7.06 | 0.18 (0.14 to 0.22) |
| High-middle SDI | 30 to 34 | 204.19(164.59-250.71) | 275.61(224.32-334.63) | 34.98 | | 489.1(394.24-600.52) | | 535.26(435.66-649.88) | 9.44 | 0.2 (0.15 to 0.25) |
| High-middle SDI | 25 to 29 | 191.22(146.71-240.65) | 177.02(134.06-222.87) | -7.43 | | 418.01(320.71-526.06) | | 439.15(332.58-552.91) | 5.06 | 0.11 (0.07 to 0.15) |
| High-middle SDI | 20 to 24 | 123.97(85.84-168.67) | 93.42(63.41-130.7) | -24.64 | | 257.51(178.3-350.37) | | 262.44(178.13-367.18) | 1.91 | 0.09 (0.05 to 0.13) |
| High-middle SDI | 15 to 19 | 34.1(23.4-47.89) | 25.38(17.12-35.97) | -25.58 | | 72.01(49.41-101.12) | | 73.7(49.71-104.45) | 2.35 | 0.08 (0.05 to 0.11) |
| Low SDI | 45 to 49 | 38.56(30.9-47.11) | 97.27(77.91-119.57) | 152.26 | | 464.49(372.17-567.44) | | 468.39(375.14-575.76) | 0.84 | 0.05 (0.04 to 0.07) |
| Low SDI | 40 to 44 | 48.65(38.53-60.6) | 129.12(102.25-161.52) | 165.38 | | 490.7(388.63-611.16) | | 494.32(391.45-618.39) | 0.74 | 0.04 (0.03 to 0.06) |
| Low SDI | 35 to 39 | 63.8(48.74-81.34) | 158.85(121.12-203.75) | 148.99 | | 495.14(378.25-631.27) | | 498.49(380.09-639.39) | 0.68 | 0.04 (0.03 to 0.05) |
| Low SDI | 30 to 34 | 70.17(51.9-91.14) | 171.88(127.42-224.94) | 144.95 | | 461.31(341.19-599.15) | | 463.06(343.28-606.01) | 0.38 | 0.03 (0.02 to 0.04) |
| Low SDI | 25 to 29 | 68.68(45.98-94.56) | 163.89(109.58-225.79) | 138.65 | | 370.75(248.22-510.5) | | 372.07(248.76-512.58) | 0.36 | 0.01 (0.01 to 0.02) |
| Low SDI | 20 to 24 | 45.63(28.55-70.04) | 110.82(69.28-172.88) | 142.85 | | 210.09(131.45-322.47) | | 210.15(131.38-327.84) | 0.03 | 0.01 (0 to 0.01) |
| Low SDI | 15 to 19 | 14.72(8.64-23.43) | 36.41(21.43-57.72) | 147.33 | | 58.57(34.38-93.23) | | 59.04(34.75-93.62) | 0.80 | 0.02 (0.01 to 0.03) |
| Low-middle SDI | 45 to 49 | 101.24(80.72-122.55) | 236.34(188.76-286.71) | 133.45 | | 466.43(371.91-564.63) | | 478.07(381.81-579.95) | 2.50 | 0.1 (0.07 to 0.13) |
| Low-middle SDI | 40 to 44 | 127.81(101.48-156.87) | 290.41(231.72-355.49) | 127.21 | | 492.65(391.16-604.63) | | 503.46(401.72-616.3) | 2.20 | 0.09 (0.06 to 0.12) |
| Low-middle SDI | 35 to 39 | 160.02(123.57-199.76) | 339.9(261.55-427.66) | 112.41 | | 499.53(385.76-623.58) | | 510.47(392.8-642.26) | 2.19 | 0.08 (0.06 to 0.11) |
| Low-middle SDI | 30 to 34 | 175.43(131.54-224.32) | 352.81(268.75-451.79) | 101.11 | | 468.53(351.31-599.09) | | 477.37(363.62-611.28) | 1.89 | 0.07 (0.05 to 0.09) |
| Low-middle SDI | 25 to 29 | 171.54(119.32-230.59) | 314.17(219.95-423.53) | 83.14 | | 382.2(265.84-513.76) | | 386.69(270.72-521.29) | 1.17 | 0.04 (0.02 to 0.06) |
| Low-middle SDI | 20 to 24 | 115.24(73.85-170.17) | 192.24(122.33-289.03) | 66.82 | | 220.96(141.61-326.29) | | 220.94(140.59-332.18) | -0.01 | 0.02 (0 to 0.03) |
| Low-middle SDI | 15 to 19 | 36.53(22.49-55.9) | 56.47(34.26-85.52) | 54.59 | | 62.18(38.28-95.15) | | 62.47(37.89-94.61) | 0.47 | 0.02 (0 to 0.04) |
| Middle SDI | 45 to 49 | 164(134.85-195.86) | 397.6(323.16-472.4) | 142.43 | | 483.89(397.87-577.89) | | 490.2(398.41-582.41) | 1.30 | 0.03 (0.01 to 0.05) |
| Middle SDI | 40 to 44 | 217.23(177.72-261.07) | 421.22(341.67-508.91) | 93.91 | | 514.07(420.57-617.82) | | 514.62(417.43-621.75) | 0.11 | 0 (-0.01 to 0.02) |
| Middle SDI | 35 to 39 | 291.57(232.01-357.7) | 481.94(377.2-597.26) | 65.29 | | 526.01(418.56-645.31) | | 525.86(411.57-651.69) | -0.03 | 0 (-0.02 to 0.02) |
| Middle SDI | 30 to 34 | 299.11(231.95-377.08) | 494.97(382.04-625.73) | 65.48 | | 498.19(386.33-628.06) | | 501.22(386.86-633.63) | 0.61 | 0 (-0.02 to 0.03) |
| Middle SDI | 25 to 29 | 316.7(231.86-411.6) | 373.86(268.26-494.53) | 18.05 | | 422.78(309.52-549.48) | | 412.66(296.09-545.85) | -2.40 | -0.07 (-0.09 to -0.04) |
| Middle SDI | 20 to 24 | 229.63(154.69-325.82) | 211.65(137.69-305.22) | -7.83 | | 259.97(175.13-368.86) | | 244.21(158.88-352.17) | -6.06 | -0.14 (-0.17 to -0.11) |
| Middle SDI | 15 to 19 | 69.37(46.36-99.24) | 63.02(40.51-92.11) | -9.15 | | 75.24(50.28-107.63) | | 71.75(46.12-104.86) | -4.63 | -0.14 (-0.18 to -0.1) |

**Table S7： Age-specific DALYs due to Schizophrenia among women of reproductive age in 1990 and 2021, with trends in age patterns from 1990 to 2021.**

| Location | Age(years) | DALY cases | | |  | | DALY rates | | |  |
| --- | --- | --- | --- | --- | --- | --- | --- | --- | --- | --- |
|  |  | 1990_thousands(95% UI) | 2021_thousands(95% UI) | Percentage change in case(100%) | | 1990_per 100 000(95% UI) | | 2021_per 100 000(95% UI) | Percentage change in ASRs(100%) | EAPC(95% CI) |
| Global | 45 to 49 | 356.88(253.07-457.25) | 747.43(532.29-962.45) | 109.44 | | 313.6(222.38-401.8) | | 317.19(225.89-408.43) | 1.144054259 | 0.04 (0.03 to 0.06) |
| Global | 40 to 44 | 465.3(331.84-598.5) | 825.47(585.85-1066.97) | 77.41 | | 331.82(236.64-426.81) | | 332.73(236.14-430.07) | 0.273688201 | 0.03 (0.01 to 0.04) |
| Global | 35 to 39 | 585.45(408.26-764.75) | 943.41(656.07-1246.16) | 61.14 | | 337.53(235.37-440.9) | | 339.6(236.16-448.58) | 0.613518398 | 0.02 (0.02 to 0.03) |
| Global | 30 to 34 | 606.24(422.02-813.32) | 965.84(668.39-1300.43) | 59.32 | | 318.89(221.99-427.82) | | 323.1(223.59-435.03) | 1.318952674 | 0.03 (0.01 to 0.05) |
| Global | 25 to 29 | 589.72(404.75-830.59) | 768.76(511.63-1085.9) | 30.36 | | 267.93(183.9-377.37) | | 264.19(175.83-373.18) | -1.398064836 | -0.02 (-0.05 to 0) |
| Global | 20 to 24 | 394.81(248.07-595.04) | 452.76(283.85-699.36) | 14.68 | | 161.72(101.61-243.73) | | 154.13(96.63-238.08) | -4.690882821 | -0.09 (-0.13 to -0.06) |
| Global | 15 to 19 | 116.58(70.66-178.83) | 133.03(80.38-206.01) | 14.11 | | 45.62(27.65-69.98) | | 43.81(26.47-67.85) | -3.969265733 | -0.11 (-0.14 to -0.08) |
| High SDI | 45 to 49 | 91.75(65.01-118.58) | 129.21(91.86-165.31) | 40.83 | | 363.13(257.28-469.32) | | 359.86(255.83-460.4) | -0.899846857 | 0.01 (-0.02 to 0.04) |
| High SDI | 40 to 44 | 116.75(83.73-149.27) | 136.54(97.43-173.96) | 16.95 | | 373.93(268.17-478.09) | | 371.93(265.39-473.85) | -0.535625637 | -0.01 (-0.03 to 0.02) |
| High SDI | 35 to 39 | 123.96(86.43-162.79) | 139.64(98-182.8) | 12.65 | | 370.7(258.46-486.81) | | 368.13(258.37-481.91) | -0.695034821 | -0.02 (-0.04 to 0) |
| High SDI | 30 to 34 | 122.96(86.58-165.16) | 128.43(90.55-170.93) | 4.45 | | 346.52(244.01-465.43) | | 343.09(241.89-456.62) | -0.98866688 | 0 (-0.02 to 0.03) |
| High SDI | 25 to 29 | 101.86(70.8-145.72) | 98.08(67.82-137.06) | -3.71 | | 284.15(197.5-406.51) | | 283.79(196.25-396.6) | -0.127553485 | 0.06 (0.03 to 0.09) |
| High SDI | 20 to 24 | 55.36(34.14-84.75) | 52.15(32.55-79.19) | -5.79 | | 164.85(101.68-252.39) | | 165.43(103.24-251.21) | 0.351224221 | 0.06 (0.01 to 0.11) |
| High SDI | 15 to 19 | 13.05(7.75-20.73) | 12.15(7.23-18.76) | -6.92 | | 40.95(24.33-65.03) | | 41.76(24.85-64.5) | 1.96210291 | 0.07 (0.03 to 0.11) |
| High-middle SDI | 45 to 49 | 75.01(53.96-95.17) | 160.48(117.17-203.5) | 113.94 | | 305.95(220.08-388.16) | | 332.75(242.94-421.96) | 8.75943472 | 0.27 (0.23 to 0.31) |
| High-middle SDI | 40 to 44 | 100.17(70.77-129.96) | 158.38(113.53-200.86) | 58.11 | | 325.81(230.19-422.72) | | 347.93(249.42-441.26) | 6.790100362 | 0.24 (0.2 to 0.28) |
| High-middle SDI | 35 to 39 | 132.69(92.94-171.49) | 178.74(125.87-233.25) | 34.70 | | 335.96(235.31-434.19) | | 360.72(254.02-470.72) | 7.369585016 | 0.2 (0.16 to 0.25) |
| High-middle SDI | 30 to 34 | 133.03(93.68-176.64) | 180.54(126.86-236.84) | 35.71 | | 318.66(224.39-423.11) | | 350.63(246.38-459.96) | 10.03473028 | 0.21 (0.17 to 0.26) |
| High-middle SDI | 25 to 29 | 125.86(89.06-172.76) | 116.69(81.38-161.51) | -7.29 | | 275.13(194.69-377.66) | | 289.48(201.89-400.67) | 5.21685655 | 0.13 (0.09 to 0.17) |
| High-middle SDI | 20 to 24 | 82.6(52.91-117.82) | 62.22(39.42-92.7) | -24.68 | | 171.58(109.91-244.75) | | 174.79(110.73-260.42) | 1.868316558 | 0.1 (0.06 to 0.15) |
| High-middle SDI | 15 to 19 | 22.96(14.06-34.79) | 17.05(10.5-25.59) | -25.76 | | 48.48(29.68-73.45) | | 49.5(30.51-74.32) | 2.109207618 | 0.08 (0.04 to 0.12) |
| Low SDI | 45 to 49 | 23.75(16.85-31.15) | 60.14(42.09-79.16) | 153.24 | | 286.07(202.94-375.17) | | 289.59(202.69-381.16) | 1.232184095 | 0.09 (0.07 to 0.1) |
| Low SDI | 40 to 44 | 30.17(21.17-39.82) | 80.54(56.5-106.63) | 166.95 | | 304.3(213.48-401.62) | | 308.36(216.32-408.22) | 1.334457358 | 0.08 (0.06 to 0.09) |
| Low SDI | 35 to 39 | 39.96(27.25-53.69) | 99.95(67.98-133.95) | 150.15 | | 310.11(211.53-416.66) | | 313.66(213.32-420.35) | 1.144927942 | 0.07 (0.06 to 0.09) |
| Low SDI | 30 to 34 | 44.3(29.37-61.13) | 109.53(73.84-150.3) | 147.25 | | 291.22(193.08-401.9) | | 295.07(198.92-404.93) | 1.322846734 | 0.07 (0.06 to 0.08) |
| Low SDI | 25 to 29 | 43.79(27.66-63.33) | 105.49(67.59-154.28) | 140.90 | | 236.41(149.35-341.88) | | 239.49(153.45-350.26) | 1.304453823 | 0.06 (0.05 to 0.07) |
| Low SDI | 20 to 24 | 29.57(17.75-47.25) | 72.33(42.87-115.18) | 144.59 | | 136.15(81.73-217.54) | | 137.17(81.3-218.42) | 0.749748282 | 0.05 (0.04 to 0.06) |
| Low SDI | 15 to 19 | 9.66(5.07-16.09) | 24.18(13.16-40.71) | 150.31 | | 38.44(20.18-64.02) | | 39.21(21.35-66.03) | 2.013540186 | 0.06 (0.05 to 0.07) |
| Low-middle SDI | 45 to 49 | 62.57(44.09-80.29) | 146.31(103.02-190.04) | 133.83 | | 288.27(203.12-369.93) | | 295.95(208.39-384.41) | 2.663250762 | 0.12 (0.09 to 0.15) |
| Low-middle SDI | 40 to 44 | 79.69(56.69-102.99) | 181.49(126.78-237.17) | 127.75 | | 307.14(218.5-396.94) | | 314.64(219.8-411.16) | 2.440978428 | 0.11 (0.09 to 0.14) |
| Low-middle SDI | 35 to 39 | 100.88(69.58-132.03) | 215.02(149.06-290.46) | 113.15 | | 314.9(217.19-412.16) | | 322.92(223.86-436.21) | 2.545143499 | 0.11 (0.08 to 0.13) |
| Low-middle SDI | 30 to 34 | 111.48(76.62-151.28) | 225.4(155.5-309.07) | 102.18 | | 297.73(204.62-404.02) | | 304.97(210.4-418.19) | 2.432192909 | 0.1 (0.08 to 0.12) |
| Low-middle SDI | 25 to 29 | 110.37(72.91-159.15) | 203.19(134.83-295.16) | 84.09 | | 245.92(162.46-354.59) | | 250.09(165.95-363.29) | 1.697952359 | 0.08 (0.05 to 0.1) |
| Low-middle SDI | 20 to 24 | 75.03(46.16-117.62) | 125.75(75-202.53) | 67.59 | | 143.86(88.5-225.51) | | 144.52(86.2-232.77) | 0.456511851 | 0.04 (0.03 to 0.06) |
| Low-middle SDI | 15 to 19 | 24.2(14.16-39.18) | 37.37(21.16-59.33) | 54.42 | | 41.19(24.1-66.68) | | 41.34(23.41-65.64) | 0.357966968 | 0.04 (0.02 to 0.06) |
| Middle SDI | 45 to 49 | 103.48(73.19-133.38) | 250.76(178.74-326.27) | 142.32 | | 305.32(215.95-393.54) | | 309.15(220.37-402.25) | 1.255694485 | 0.04 (0.02 to 0.06) |
| Middle SDI | 40 to 44 | 138.11(98.79-179.71) | 267.92(190.5-349.87) | 93.99 | | 326.84(233.8-425.3) | | 327.32(232.75-427.45) | 0.147845774 | 0.01 (0 to 0.03) |
| Middle SDI | 35 to 39 | 187.48(131.93-243.64) | 309.42(214.67-408.29) | 65.04 | | 338.22(238.01-439.54) | | 337.61(234.23-445.5) | -0.177946439 | 0.01 (-0.01 to 0.02) |
| Middle SDI | 30 to 34 | 193.97(134.95-260.7) | 321.31(223.6-430.94) | 65.65 | | 323.08(224.77-434.22) | | 325.37(226.42-436.38) | 0.710295205 | 0.01 (-0.01 to 0.03) |
| Middle SDI | 25 to 29 | 207.4(142.59-291.36) | 244.79(164.5-346.61) | 18.03 | | 276.87(190.36-388.96) | | 270.19(181.57-382.57) | -2.414662536 | -0.06 (-0.09 to -0.03) |
| Middle SDI | 20 to 24 | 151.97(93.29-229.91) | 140.01(88.36-214.27) | -7.87 | | 172.04(105.62-260.28) | | 161.55(101.95-247.23) | -6.10093723 | -0.13 (-0.17 to -0.1) |
| Middle SDI | 15 to 19 | 46.62(28.31-71.8) | 42.19(25.33-65.15) | -9.49 | | 50.56(30.71-77.87) | | 48.03(28.83-74.17) | -4.994387298 | -0.14 (-0.18 to -0.1) |

**Table S8： Decomposition analysis of Schizophrenia burden among women of reproductive age from 1990 to 2021.**

| location | sex | cause | measure | Overll difference | Aging | Population | Epidemiological change |
| --- | --- | --- | --- | --- | --- | --- | --- |
| Global | Female | Schizophrenia | DALYs | 1721718.58 | 264006.97  (15.33%) | 1470356.55  (85.4%) | -12644.93  (-0.73%) |
| Global | Female | Schizophrenia | Prevalence | 2701874.84 | 426846.43  (15.8%) | 2288881.96  (84.71%) | -13853.55  (-0.51%) |
| Global | Female | Schizophrenia | Incidence | 134968.8 | -27222.4  (-20.17%) | 166933.45  (123.68%) | -4742.25  (-3.51%) |
| Middle SDI | Female | Schizophrenia | DALYs | 547363.52 | 145438.24  (26.57%) | 415368.39  (75.89%) | -13443.1  (-2.46%) |
| Middle SDI | Female | Schizophrenia | Prevalence | 856647.86 | 233664.92  (27.28%) | 642539.22  (75.01%) | -19556.28  (-2.28%) |
| Middle SDI | Female | Schizophrenia | Incidence | 27245.01 | -15617.99  (-57.32%) | 47436.89  (174.11%) | -4573.88  (-16.79%) |
| High-middle SDI | Female | Schizophrenia | DALYs | 201761.05 | 77654.59  (38.49%) | 72197.59  (35.78%) | 51908.86  (25.73%) |
| High-middle SDI | Female | Schizophrenia | Prevalence | 312176.09 | 124950.12  (40.03%) | 111167.42  (35.61%) | 76058.56  (24.36%) |
| High-middle SDI | Female | Schizophrenia | Incidence | 920.81 | -9675.38  (-1050.74%) | 7552.81  (820.23%) | 3043.38  (330.51%) |
| Low-middle SDI | Female | Schizophrenia | DALYs | 570309.24 | 50902.8  (8.93%) | 502521.04  (88.11%) | 16885.41  (2.96%) |
| Low-middle SDI | Female | Schizophrenia | Prevalence | 894527.46 | 82670.22  (9.24%) | 790087.13  (88.32%) | 21770.11  (2.43%) |
| Low-middle SDI | Female | Schizophrenia | Incidence | 59393.48 | -3507.47  (-5.91%) | 61461.31  (103.48%) | 1439.63  (2.42%) |
| High SDI | Female | Schizophrenia | DALYs | 70507.86 | 27787.99  (39.41%) | 46257.38  (65.61%) | -3537.51  (-5.02%) |
| High SDI | Female | Schizophrenia | Prevalence | 119217.82 | 45113.87  (37.84%) | 72479.57  (60.8%) | 1624.38  (1.36%) |
| High SDI | Female | Schizophrenia | Incidence | 2160.85 | -3268.75  (-151.27%) | 4427.25  (204.88%) | 1002.36  (46.39%) |
| Low SDI | Female | Schizophrenia | DALYs | 330967.42 | 1529.44  (0.46%) | 324714.14  (98.11%) | 4723.84  (1.43%) |
| Low SDI | Female | Schizophrenia | Prevalence | 518028.44 | 2634.22  (0.51%) | 512333  (98.9%) | 3061.22  (0.59%) |
| Low SDI | Female | Schizophrenia | Incidence | 45188.98 | -384.65  (-0.85%) | 45373.85  (100.41%) | 199.78  (0.44%) |

**Table S9： Projected global, Chinese, and Indian burden of Schizophrenia among women of reproductive age in 2040 using the BAPC model.**

| location | year | Incidence | |  | Prevalence | |  | DALYs | |
| --- | --- | --- | --- | --- | --- | --- | --- | --- | --- |
|  |  | Number of cases | ASR per 100,000 | | Number of cases | ASR per 100,000 | | Number of cases | ASR per 100,000 |
| Global | 2022 | 522207 | 26.57 | | 7706460 | 371.23 | | 4963876.845 | 243.02 |
| Global | 2023 | 524353 | 26.56 | | 7762459 | 371.02 | | 4993948.956 | 242.71 |
| Global | 2024 | 526551 | 26.55 | | 7818333 | 370.78 | | 5023539.556 | 242.41 |
| Global | 2025 | 528827 | 26.54 | | 7874612 | 370.55 | | 5052479.565 | 242.10 |
| Global | 2026 | 531221 | 26.53 | | 7932068 | 370.37 | | 5081292.124 | 241.82 |
| Global | 2027 | 533736 | 26.52 | | 7989847 | 370.21 | | 5109750.349 | 241.55 |
| Global | 2028 | 536426 | 26.52 | | 8048772 | 370.07 | | 5138258.563 | 241.29 |
| Global | 2029 | 539229 | 26.51 | | 8108852 | 369.94 | | 5166499.188 | 241.04 |
| Global | 2030 | 542066 | 26.50 | | 8168987 | 369.84 | | 5193507.738 | 240.78 |
| Global | 2031 | 544873 | 26.50 | | 8228623 | 369.81 | | 5219123.048 | 240.55 |
| Global | 2032 | 547651 | 26.49 | | 8285535 | 369.81 | | 5242364.46 | 240.31 |
| Global | 2033 | 550107 | 26.48 | | 8336679 | 369.80 | | 5261675.875 | 240.07 |
| Global | 2034 | 552470 | 26.47 | | 8383188 | 369.79 | | 5277467.656 | 239.83 |
| Global | 2035 | 554747 | 26.46 | | 8427311 | 369.78 | | 5290734.794 | 239.56 |
| Global | 2036 | 556948 | 26.46 | | 8472122 | 369.83 | | 5303348.115 | 239.31 |
| Global | 2037 | 559163 | 26.45 | | 8519837 | 369.91 | | 5316822.962 | 239.06 |
| Global | 2038 | 561110 | 26.44 | | 8566239 | 370.03 | | 5328860.121 | 238.84 |
| Global | 2039 | 562931 | 26.43 | | 8612175 | 370.19 | | 5339745.559 | 238.64 |
| Global | 2040 | 564707 | 26.42 | | 8660800 | 370.43 | | 5351596.195 | 238.46 |
| China | 2022 | 98006 | 32.72 | | 1631177 | 420.90 | | 1107742.142 | 300.34 |
| China | 2023 | 96828 | 33.01 | | 1615356 | 421.72 | | 1100982.765 | 302.83 |
| China | 2024 | 95732 | 33.30 | | 1599673 | 422.44 | | 1094276.932 | 305.38 |
| China | 2025 | 94739 | 33.60 | | 1584814 | 423.18 | | 1087721.243 | 307.98 |
| China | 2026 | 93884 | 33.91 | | 1571033 | 423.95 | | 1081404.644 | 310.62 |
| China | 2027 | 93229 | 34.21 | | 1558841 | 424.85 | | 1075851.565 | 313.34 |
| China | 2028 | 92750 | 34.53 | | 1548865 | 425.80 | | 1071798.757 | 316.15 |
| China | 2029 | 92424 | 34.84 | | 1541229 | 426.81 | | 1069060.361 | 319.02 |
| China | 2030 | 92284 | 35.16 | | 1534622 | 427.91 | | 1066599.221 | 321.96 |
| China | 2031 | 92379 | 35.49 | | 1527322 | 429.10 | | 1063307.532 | 324.95 |
| China | 2032 | 92868 | 35.82 | | 1518043 | 430.42 | | 1058646.427 | 328.04 |
| China | 2033 | 92929 | 36.15 | | 1502636 | 431.79 | | 1050073.806 | 331.24 |
| China | 2034 | 92876 | 36.49 | | 1483157 | 433.18 | | 1038745.601 | 334.54 |
| China | 2035 | 92791 | 36.84 | | 1462048 | 434.65 | | 1026163.877 | 337.95 |
| China | 2036 | 92765 | 37.18 | | 1441637 | 436.25 | | 1013777.861 | 341.44 |
| China | 2037 | 92971 | 37.54 | | 1425290 | 438.08 | | 1003917.715 | 345.05 |
| China | 2038 | 92835 | 37.90 | | 1405859 | 440.14 | | 991421.7117 | 348.79 |
| China | 2039 | 92538 | 38.26 | | 1384834 | 442.44 | | 976910.7286 | 352.64 |
| China | 2040 | 92098 | 38.63 | | 1365465 | 445.01 | | 962869.3639 | 356.62 |
| India | 2022 | 96661 | 24.74 | | 1426373 | 352.94 | | 905054.3608 | 235.89 |
| India | 2023 | 97355 | 24.71 | | 1447917 | 352.75 | | 916708.1914 | 235.40 |
| India | 2024 | 97964 | 24.68 | | 1469106 | 352.57 | | 927956.1126 | 234.91 |
| India | 2025 | 98488 | 24.64 | | 1489771 | 352.41 | | 938630.4426 | 234.41 |
| India | 2026 | 98944 | 24.61 | | 1510099 | 352.33 | | 948801.5972 | 233.93 |
| India | 2027 | 99351 | 24.57 | | 1530014 | 352.32 | | 958424.3697 | 233.48 |
| India | 2028 | 99727 | 24.53 | | 1549621 | 352.37 | | 967503.6107 | 233.03 |
| India | 2029 | 100052 | 24.49 | | 1568942 | 352.46 | | 976013.7304 | 232.58 |
| India | 2030 | 100281 | 24.45 | | 1587711 | 352.62 | | 983772.5059 | 232.14 |
| India | 2031 | 100384 | 24.41 | | 1606046 | 352.95 | | 990893.0587 | 231.73 |
| India | 2032 | 100312 | 24.37 | | 1623527 | 353.39 | | 997227.2329 | 231.34 |
| India | 2033 | 100189 | 24.32 | | 1640200 | 353.90 | | 1002952.957 | 230.97 |
| India | 2034 | 100024 | 24.28 | | 1656088 | 354.48 | | 1008015.492 | 230.61 |
| India | 2035 | 99776 | 24.23 | | 1671170 | 355.13 | | 1012288.466 | 230.26 |
| India | 2036 | 99419 | 24.18 | | 1685785 | 355.93 | | 1015983.511 | 229.96 |
| India | 2037 | 98918 | 24.14 | | 1699572 | 356.83 | | 1018942.085 | 229.69 |
| India | 2038 | 98371 | 24.09 | | 1713383 | 357.77 | | 1021854.902 | 229.45 |
| India | 2039 | 97787 | 24.04 | | 1726963 | 358.71 | | 1024609.135 | 229.23 |
| India | 2040 | 97164 | 23.99 | | 1739793 | 359.68 | | 1026836.557 | 229.03 |
